# Supplementary material for: Lysosomal TBK1 responds to amino acid availability to relieve Rab7-dependent mTORC1 inhibition
Source: EMBO J. 2024 Aug 5;43(18):7. doi: 10.1038/s44318-024-00180-8 (PMC11405869; doi:10.1038/s44318-024-00180-8)
Supplement: Supplementary file 1 — Appendix [file 44318_2024_180_MOESM1_ESM.pdf]

Appendix for:

Lysosomal TBK1 Responds to Amino Acid Availability to Relieve Rab7-Dependent mTORC1 Inhibition

Gabriel Talaia[1,2,3,4,6], Amanda Bentley-DeSousa[1,2,3,4,6] and Shawn M. Ferguson[1,2,3,4,5,6*]

Departments of Cell Biology[1] and Neuroscience[2], Program in Cellular Neuroscience, Neurodegeneration and Repair[3], Wu Tsai Institute[4], Kavli Institute for Neuroscience[5], Yale University School of Medicine, New Haven, Connecticut 06510, USA. Aligning Science Across Parkinson's (ASAP) Collaborative Research Network, Chevy Chase, MD, 20815, USA.[6]

*Correspondence: shawn.ferguson@yale.edu

Table of Contents

Appendix Tables: Pages 3-5

Appendix Table S1 - Summary of cell lines used in this study.

Cell Line	RRID	Genotype	Reference
HeLa M	CVCL_R965	WT	Provided by Pietro De Camilli, Yale University
HeLa M	CVCL_D7ES	TBK1 KO	This work
HeLa M	CVCL_D7EY	TBK1 KO + TBK1-GFP	This work
HeLa M	CVCL_D7EV	TBK1 KO + TBK1-E696K-GFP	This work
HeLa M	CVCL_D7ET	RAB7A KO	This work
HeLa M	CVCL_D7EW	RAB7A KO + mCherry-RAB7	This work
HeLa M	CVCL_D7EX	RAB7A KO + mCherry-RAB7-S72A	This work
HeLa M	CVCL_D7EU	TBK1 KO + RAB7A KO	This work
RAW 264.7	CVCL_0493	WT	ATCC
RAW 264.7	CVCL_D7F3	STING KO	Bentley-DeSousa and Ferguson, bioRxiv, 2023
RAW 264.7	CVCL_D7F7	TBK1 KO + IKKε KO	Bentley-DeSousa and Ferguson, bioRxiv, 2023

Appendix Table S2 - Summary of nutrients and drug treatments used in this study.

Compounds/Drugs	Company	Product No
DMEM	Thermo Fisher Scientific	11965-092
HI-FBS	Thermo Fisher Scientific	16140-071
Penicillin/Streptomycin (10,000 U/mL)	Thermo Fisher Scientific	15140122
Puromycin	Thermo Fisher Scientific	A11138-03
RPMI 1640 Medium Modified w/o Amino acids	USBiological	R9010-01
MEM Amino Acids	Gibco	11130-051
2',3'-cGAMP	Chemietek	CT-CGMAP
BX-795	Cayman Chemical	14932
Torin-1	Tocris	4247

Appendix Table S3 - Summary of plasmids used in this study.

Plasmid	Reference	RRID
pSpCas9-2A-Puro-TBK1-gRNA (PX459)	This paper	RRID:Addgene_221550
pSpCas9-2A-Puro-RAB7A-gRNA1 (PX459)	This paper	RRID:Addgene_221551
pSpCas9-2A-Puro-RAB7A-gRNA2 (PX459)	This paper	RRID:Addgene_221552

pEIF1A-piggyBac transposase	Michael Ward (NINDS) (Pantazis et al., 2022)	RRID:Addgene_ 172116
pPB-EF1A-Puro-hTBK1-EGFP	This paper	RRID:Addgene_221553
pPB-EF1A-Puro-hTBK1-E696K-EGFP	This paper	RRID:Addgene_221554
pPB-EF1A-Puro-mCherry-RAB7	This paper	RRID:Addgene_221555
pPB-EF1A-Puro-mCherry-RAB7-S72A	This paper	RRID:Addgene_221556
pCMV-mCherry-RAB7	Gia Voeltz (U. Colorado-Boulder) (Rowland et al, 2014)	RRID:Addgene_61804
pCMV-myc-RAB7	Christopher Burd, Yale University	N/A
pCMV-myc-RAB7-T22N	Christopher Burd, Yale University	N/A

Appendix Table S4 - Sequences of oligonucleotides used in this study.

**Primer**	**Sequence (5'-3')**
RAB7_F	CAAAAAAGCAGGCTGCCACCATGGTGAGCAAGGGCGAG
RAB7_R	TCAGCAACTGCAGCTTTCTG
PB-EF1_F	CAGAAAGCTGCAGTTGCTGAACCCAGCTTTCTTGTACAAAGTG
PB-EF1_R	GGTGGCAGCCTGCTTTTTTG
RAB7-S72A_F	ACGGTTCCAGGCTCTCGGTGT
RAB7-S72A_R	TCCTGTCCTGCTGTGTCC
TBK1-E699K_F	ATTAAAGGAAAAGATGGAAGG
TBK1-E699K_R	TTCTTCATACCAAGAGTC
TBK1 gRNA sense	CACCGCATAAGCTTCCTTCGTCCAG
TBK1 gRNA antisense	AAACCTGGACGAAGGAAGCTTATGC
RAB7A gRNA1 sense	CACCGGTCATCCACCATCACCTCCT
RAB7A gRNA1 antisense	AAACAGGAGGTGATGGTGGATGACC
RAB7A gRNA2 sense	CACCGCATTCAAAACCCTAGATAGC
RAB7A gRNA2 antisense	AAACGCTATCTAGGGTTTTGAATGC

Appendix Table S5 - Description of antibodies used in this study.

**Antibody**	**Concentration**	**Company**	**Product number**	**RRID**
S6K1	1:2000	Cell Signaling Technologies	9202L	AB_331676
P-S6K1 (T389)	1:1000	Cell Signaling Technologies	9234S	AB_2269803
ULK1	1:2000	Cell Signaling Technologies	8054S	AB_11178668
P-ULK1 (S757)	1:2000	Cell Signaling Technologies	6888S	AB_10829226
S6	1:6000	Cell Signaling Technologies	2217S	AB_331355

P-S6 (S235/236)	1:2000	Cell Signaling Technologies	4858S	AB_916156
mTOR	1:2000	Cell Signaling Technologies	2983	AB_2105622
Rab7 (E9O7E)	1:4000	Cell Signaling Technologies	95746	AB_2800252
P-Rab7 (S72)	1:1000	Abcam	ab302494	AB_2933985
STING	1:1000	Cell Signaling Technologies	13647S	AB_2732796
P-STING (S366)	1:1000	Cell Signaling Technologies	50907S	AB_2827656
TBK1	1:2000, 1:500	Cell Signaling Technologies	3504S	AB_2255663
P-TBK1 (S172)	1:1000	Cell Signaling Technologies	5483S	AB_10693472
LAMP1 (D2D11)	1:2000	Cell Signaling Technologies	9091S	AB_2687579
RAGC (D8H5)	1:1000	Cell Signaling Technologies	9480S	AB_10614716
LAMP1 (H4A3)	1:2000	DSHB	H4A3	AB_2296838
GFP	1:500	Invitrogen	A11120	AB_221568
PDI	1:1000	Cell Signaling Technologies	2446S	AB_2298935
GM130	1:2000	BD Biosciences	610822	AB_398141
Rabbit IgG (HRP)	1:2000	Cell Signaling Technologies	7074S	AB_2099233
Mouse IgG (HRP)	1:2000	Cell Signaling Technologies	7076S	AB_330924
Biotin (HRP)	1:4000	Cell Signaling Technologies	7075S	AB_10696897
Alexa 488-Rabbit	1:600	Invitrogen	A21206	AB_2535792
Alexa 568-Rabbit	1:600	Invitrogen	A10042	AB_2534017
Alexa 647-Mouse	1:600	Invitrogen	A21202	AB_141607
