# Supplementary material for: Lysosomal TBK1 responds to amino acid availability to relieve Rab7-dependent mTORC1 inhibition
Source: EMBO J. 2024 Aug 5;43(18):7. doi: 10.1038/s44318-024-00180-8 (PMC11405869; doi:10.1038/s44318-024-00180-8)

Figure 1 - Data Source

A)

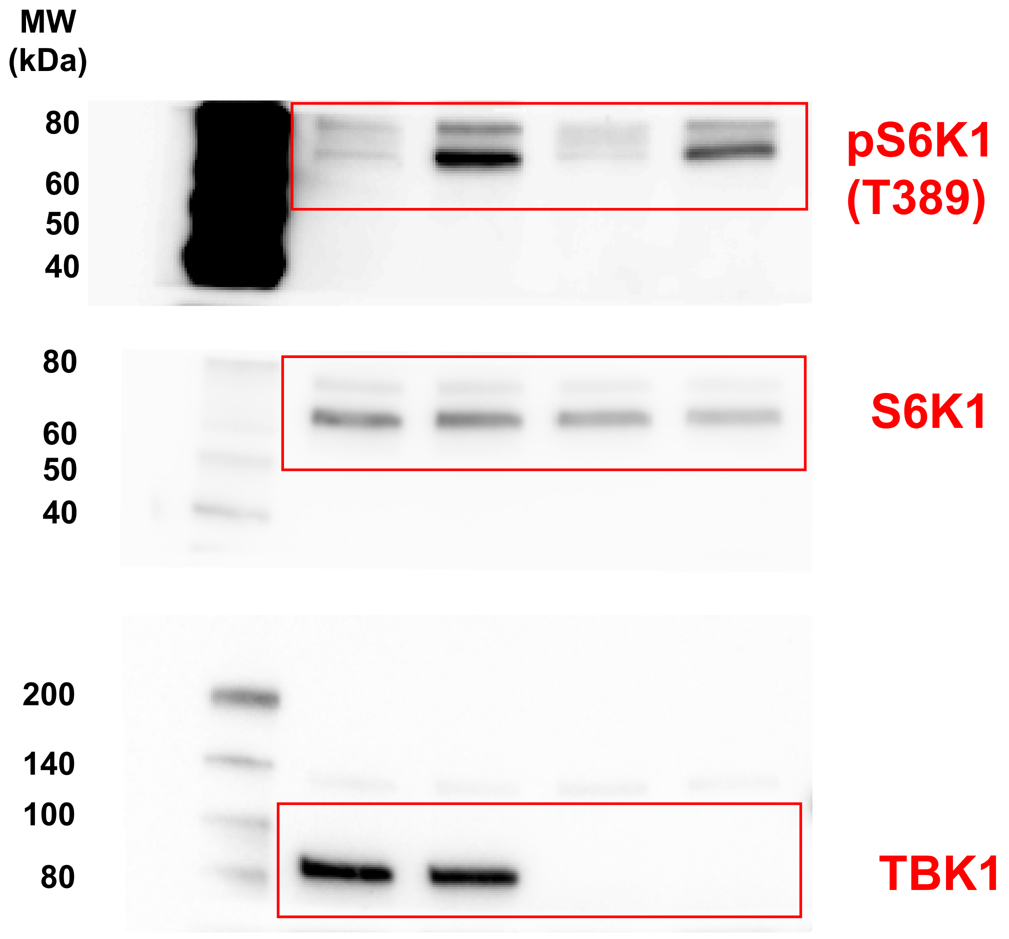

C)

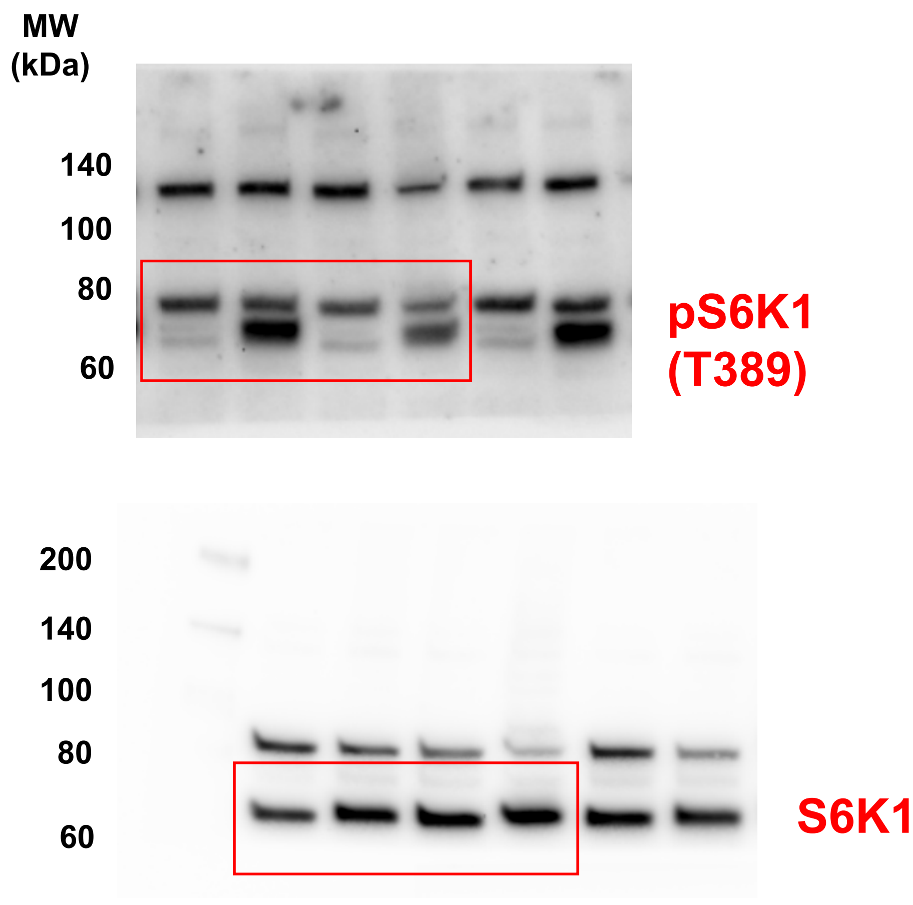

H)

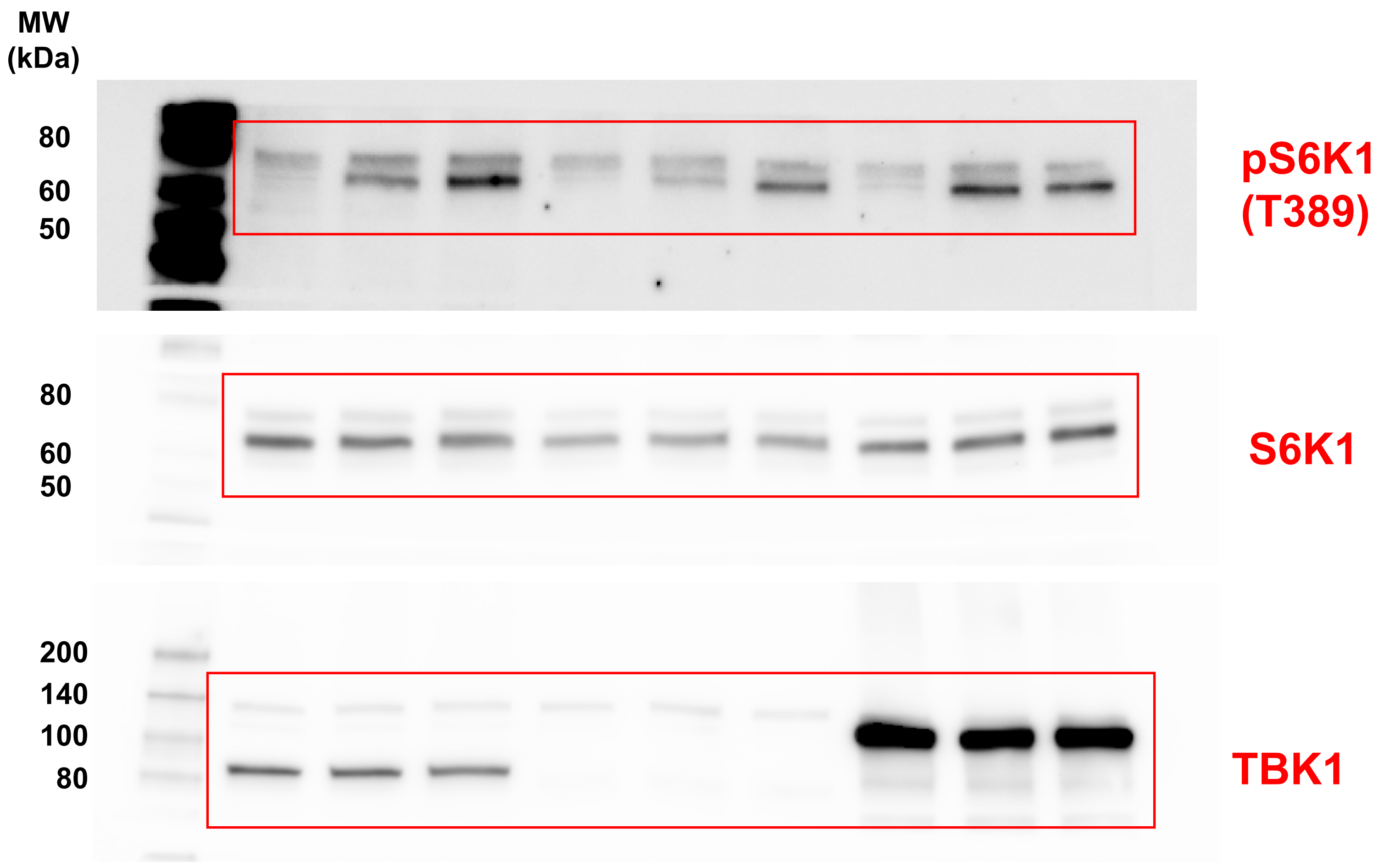

Figure 1 - Data Source

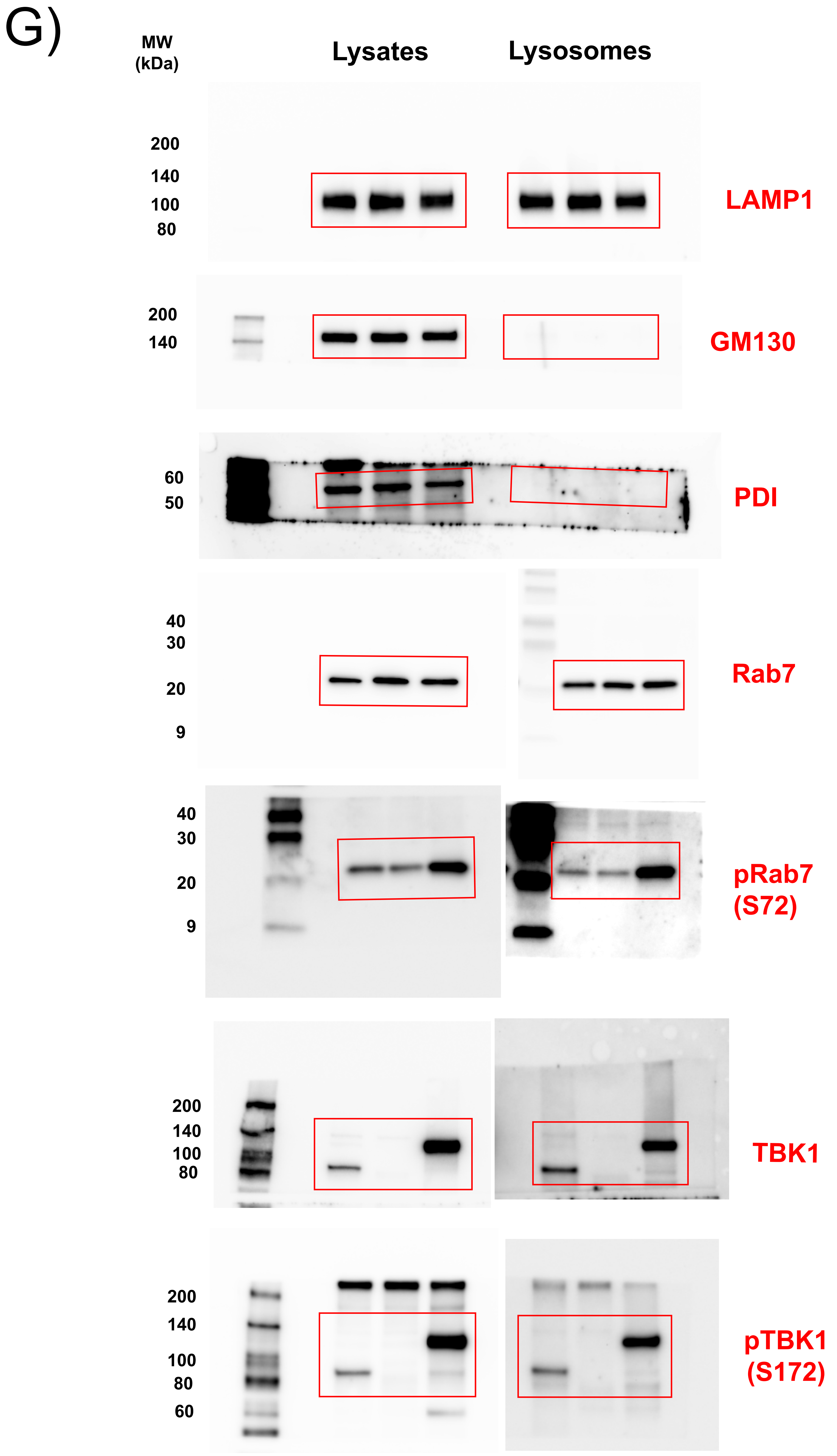

Figure 2 - Data Source

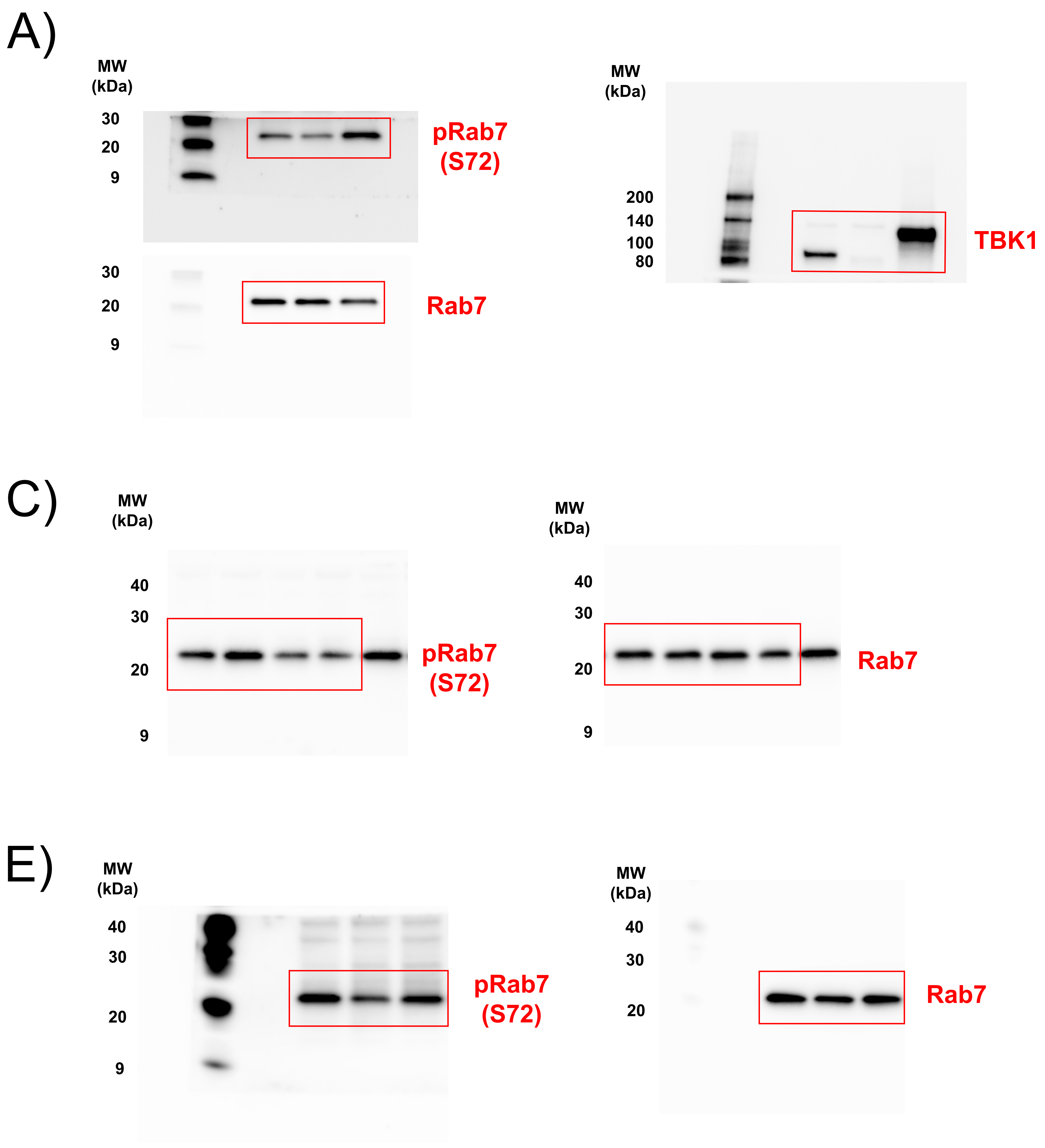

Figure 2 - Data Source

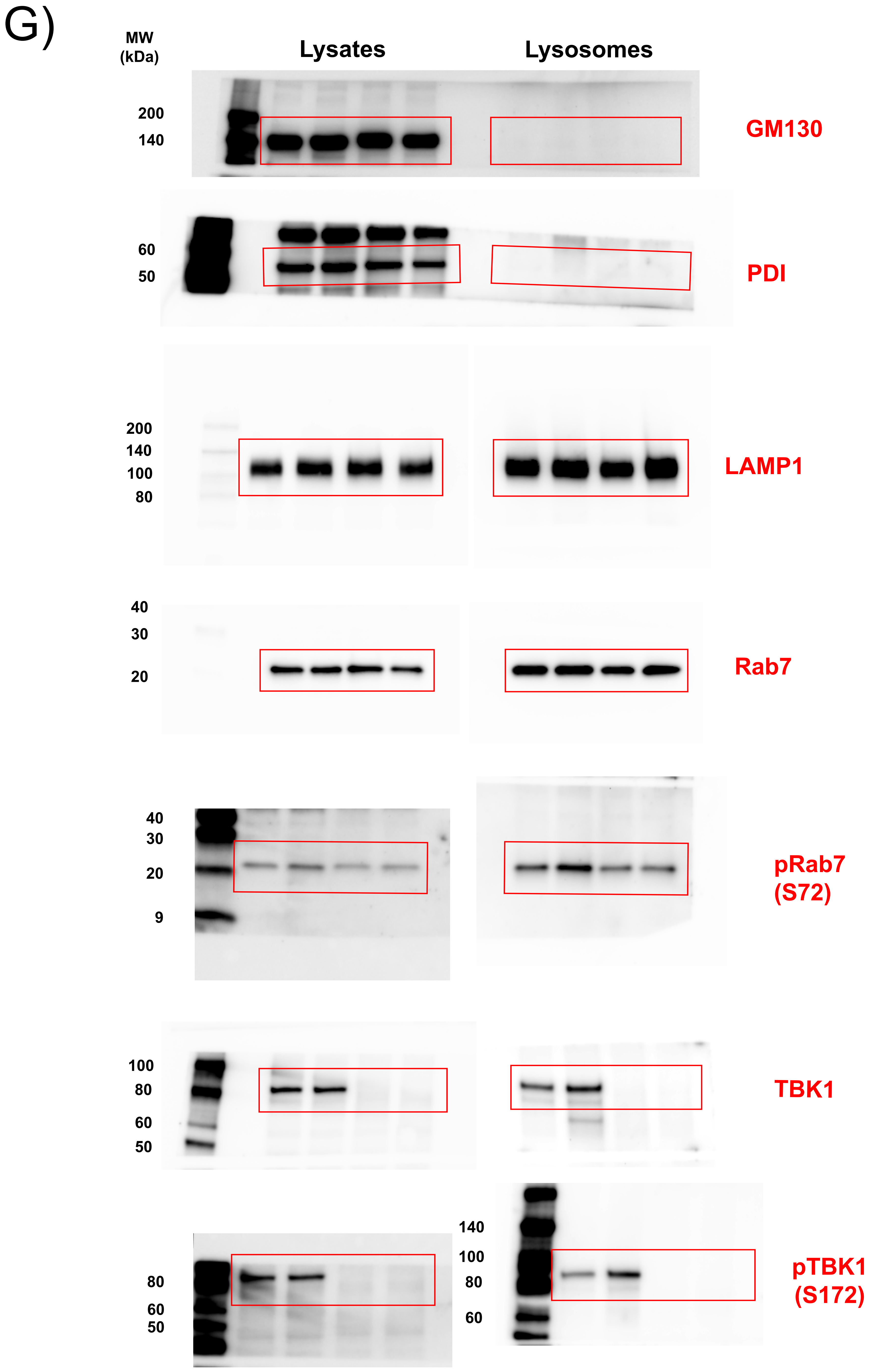

Figure 3 - Data Source

A)

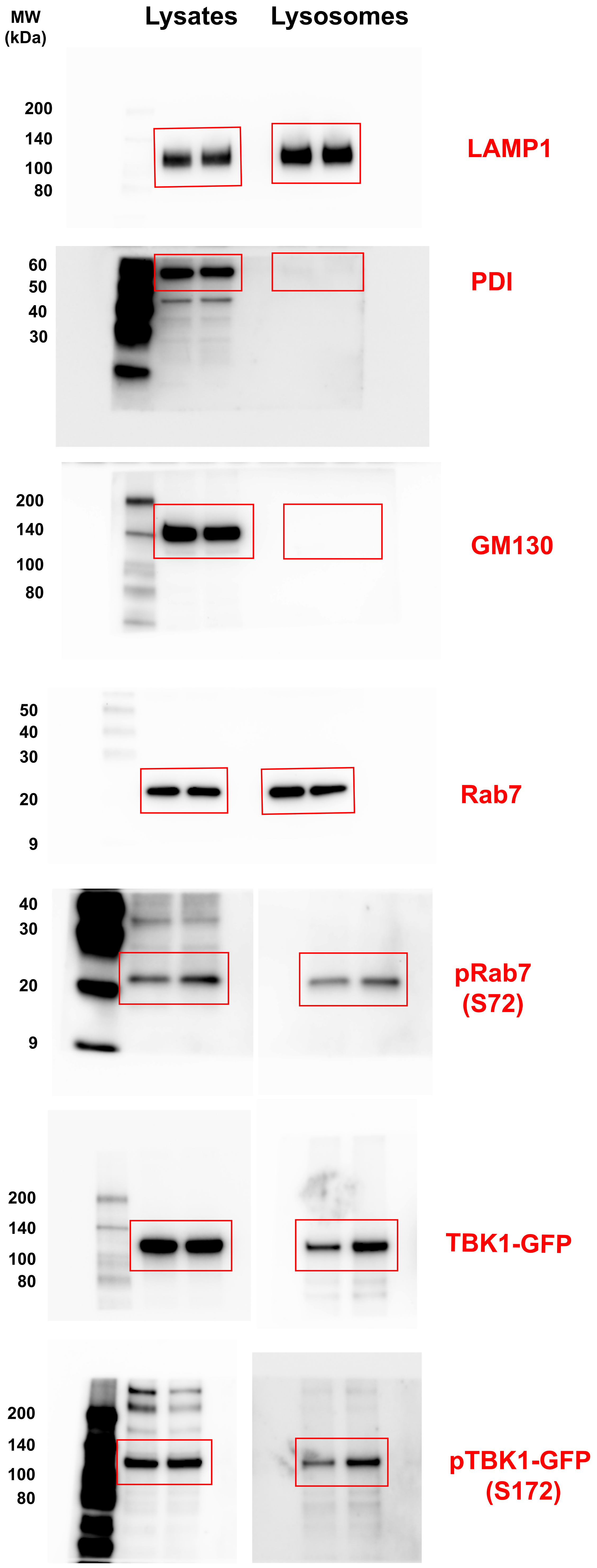

Figure 4 - Data Source

A)

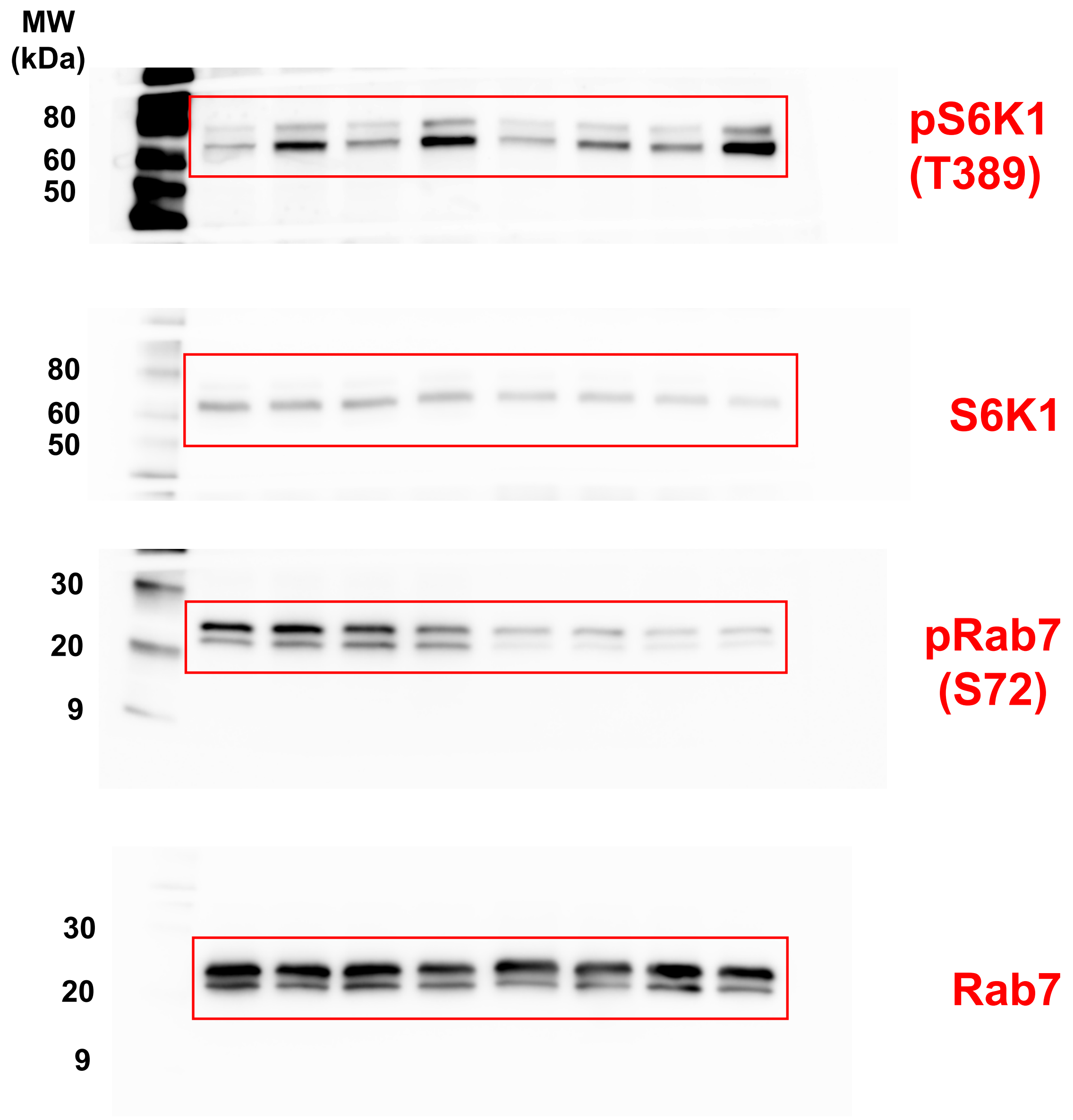

B)

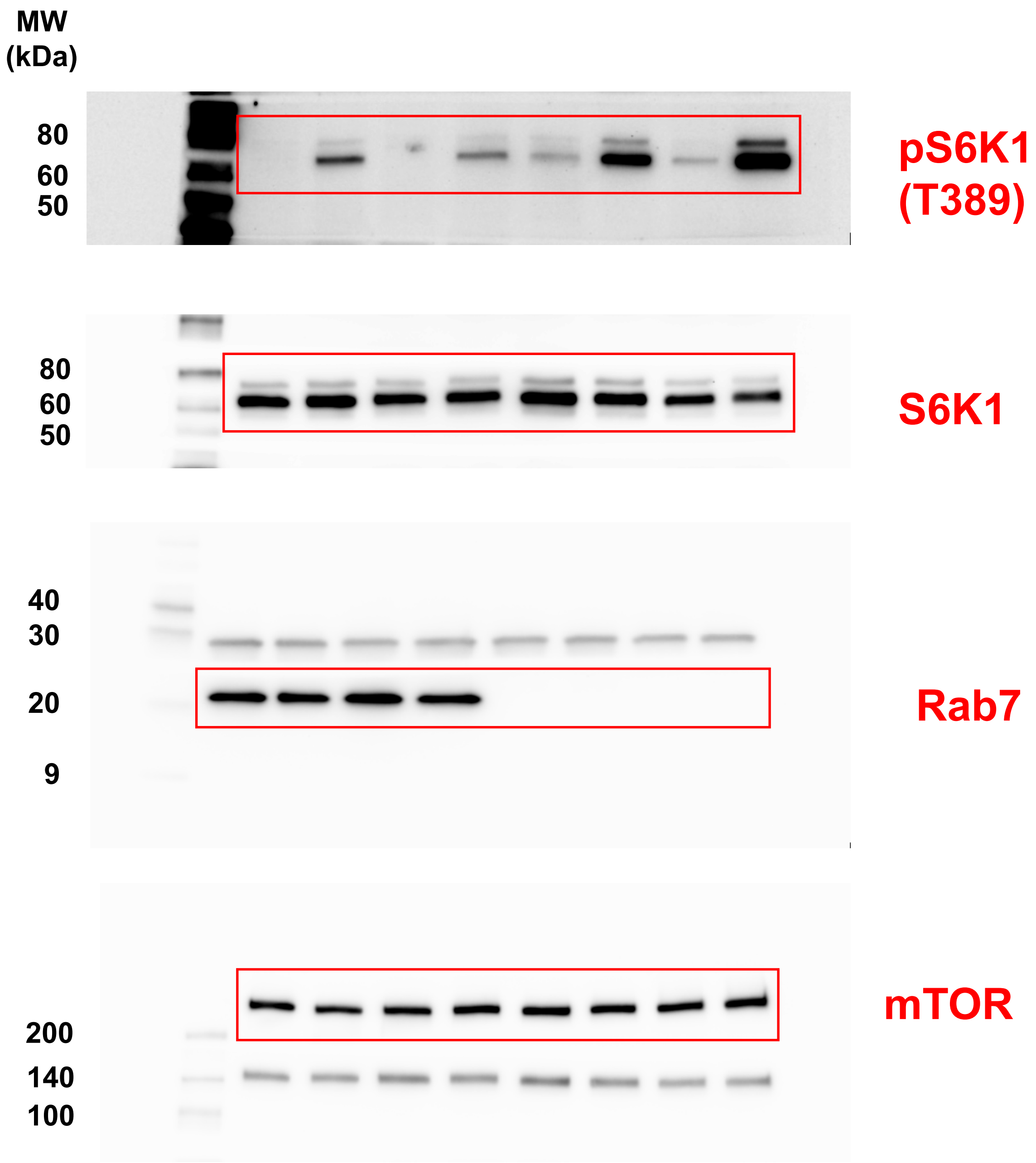

Figure 4 - Data Source

D)

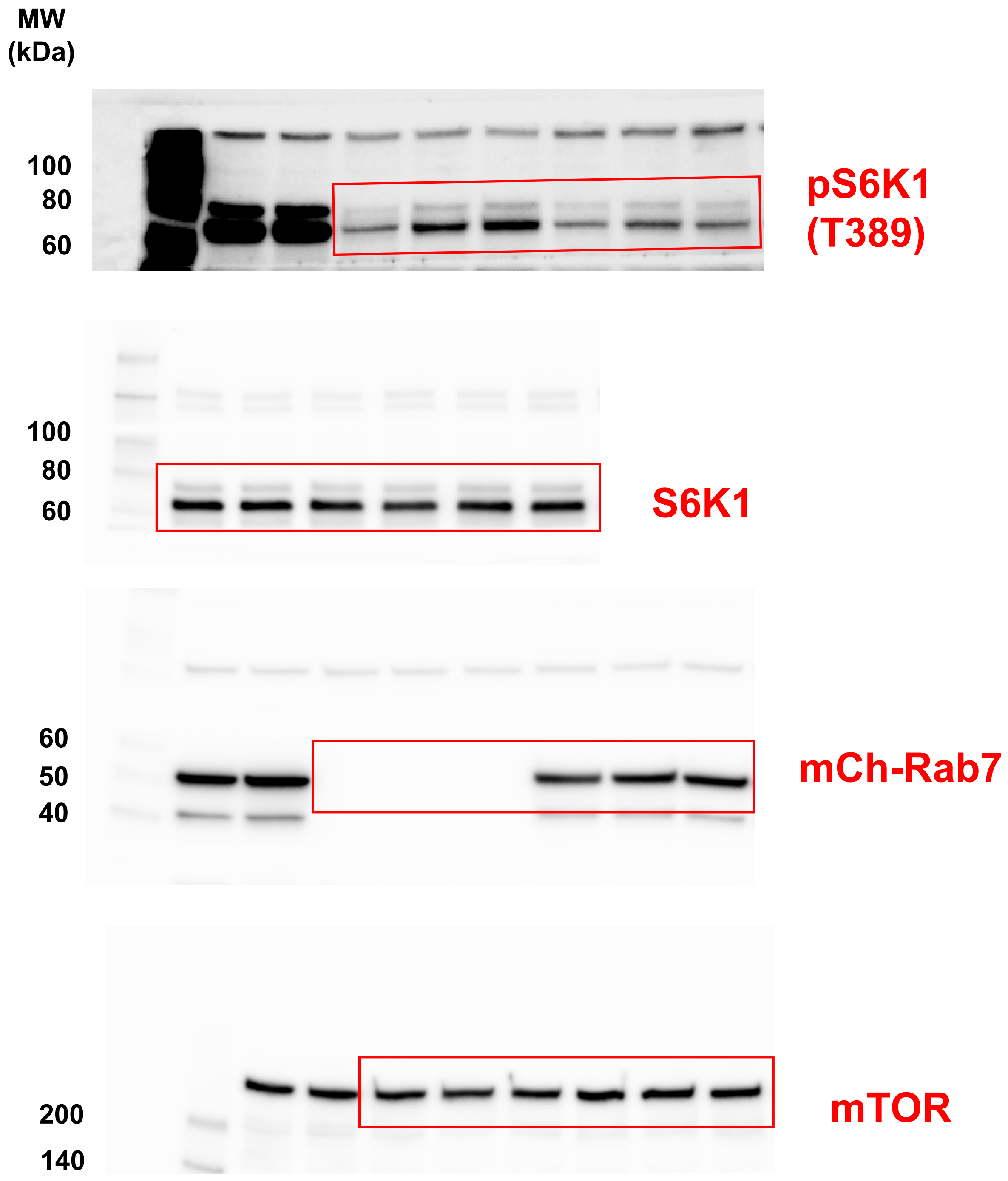

Figure 5 - Data Source

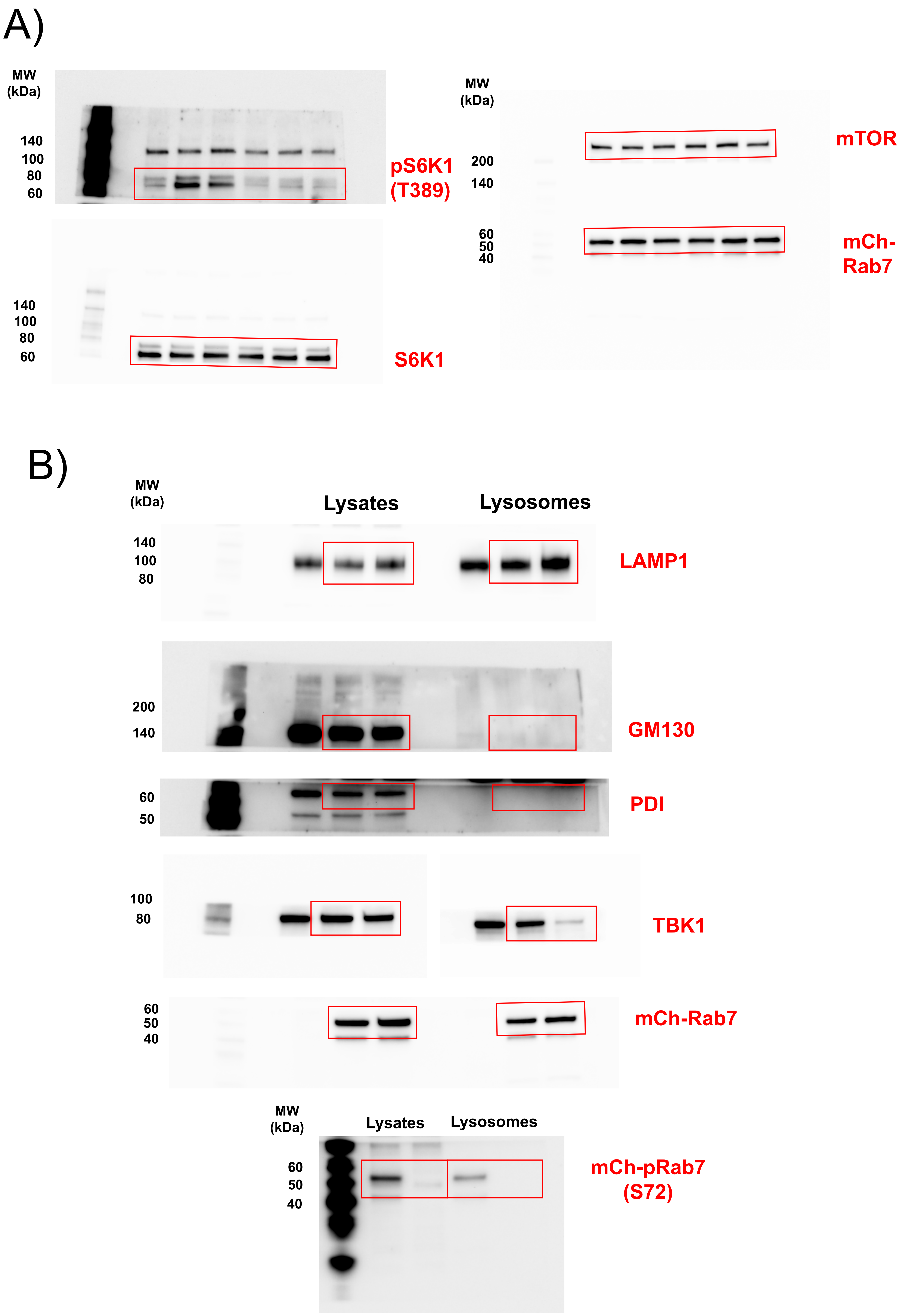

Figure 6 - Data Source

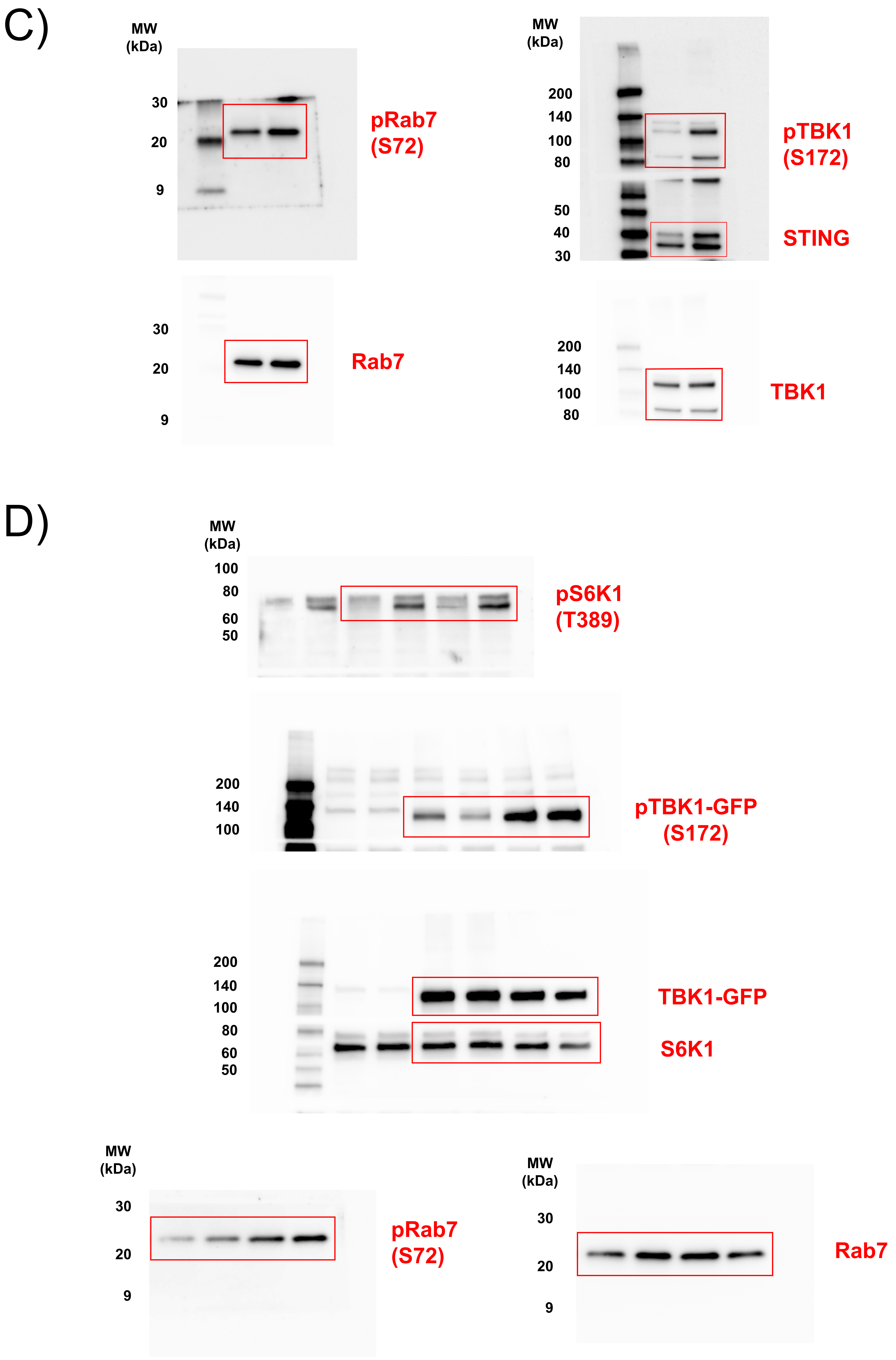

Supplement: Supplementary file 3 — Source data Fig. 1 [file 44318_2024_180_MOESM3_ESM.zip › Data Source Blots- Talaia et al, 2024.pdf]
