# Supplementary figures and images for: Lysosomal TBK1 responds to amino acid availability to relieve Rab7-dependent mTORC1 inhibition (part 1 of 3)
Source: EMBO J. 2024 Aug 5;43(18):7. doi: 10.1038/s44318-024-00180-8 (PMC11405869; doi:10.1038/s44318-024-00180-8)

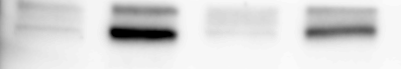

Supplement: Supplementary file 3 — Source data Fig. 1 [file 44318_2024_180_MOESM3_ESM.zip › 1A/pS6K1-T389 western cropped.tif]

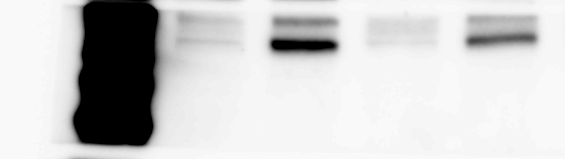

Supplement: Supplementary file 3 — Source data Fig. 1 [file 44318_2024_180_MOESM3_ESM.zip › 1A/pS6K1-T389 western.tif]

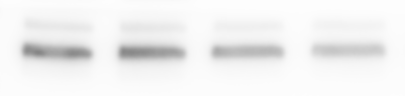

Supplement: Supplementary file 3 — Source data Fig. 1 [file 44318_2024_180_MOESM3_ESM.zip › 1A/S6K1 western cropped.tif]

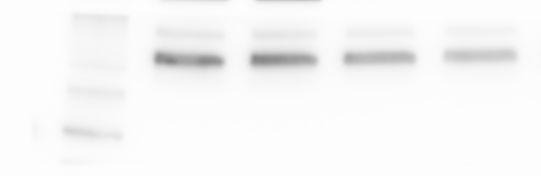

Supplement: Supplementary file 3 — Source data Fig. 1 [file 44318_2024_180_MOESM3_ESM.zip › 1A/S6K1 western raw.tif]

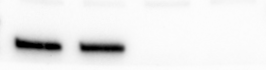

Supplement: Supplementary file 3 — Source data Fig. 1 [file 44318_2024_180_MOESM3_ESM.zip › 1A/TBK1 western cropped.tif]

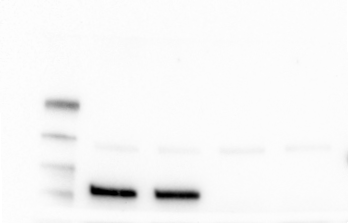

Supplement: Supplementary file 3 — Source data Fig. 1 [file 44318_2024_180_MOESM3_ESM.zip › 1A/TBK1 western.tif]

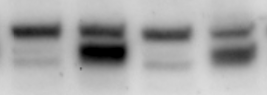

Supplement: Supplementary file 3 — Source data Fig. 1 [file 44318_2024_180_MOESM3_ESM.zip › 1C/pS6K1-T389 western cropped.tif]

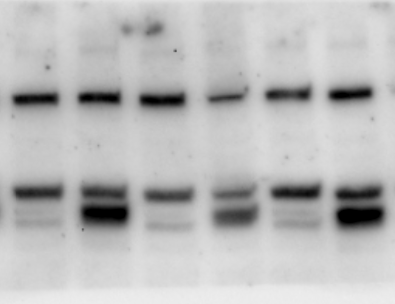

Supplement: Supplementary file 3 — Source data Fig. 1 [file 44318_2024_180_MOESM3_ESM.zip › 1C/pS6K1-T389 western.tif]

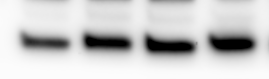

Supplement: Supplementary file 3 — Source data Fig. 1 [file 44318_2024_180_MOESM3_ESM.zip › 1C/S6K1 western cropped.tif]

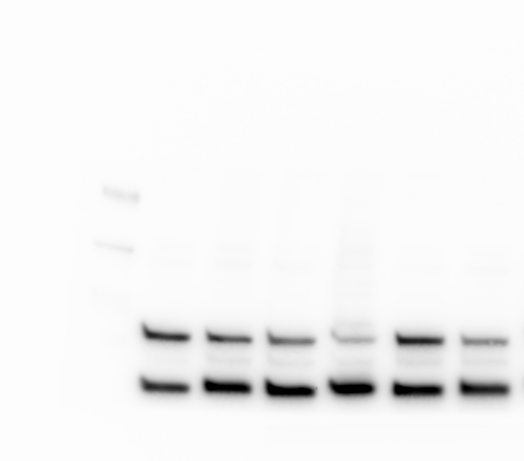

Supplement: Supplementary file 3 — Source data Fig. 1 [file 44318_2024_180_MOESM3_ESM.zip › 1C/S6K1 western.tif]

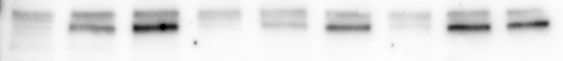

Supplement: Supplementary file 3 — Source data Fig. 1 [file 44318_2024_180_MOESM3_ESM.zip › 1E/pS6K1-T389 western cropped.tif]

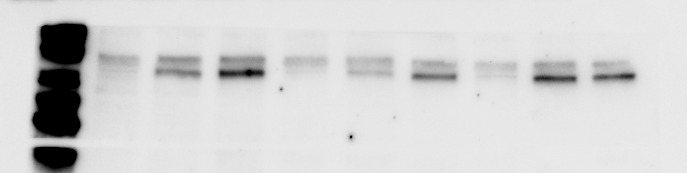

Supplement: Supplementary file 3 — Source data Fig. 1 [file 44318_2024_180_MOESM3_ESM.zip › 1E/pS6K1-T389 western.tif]

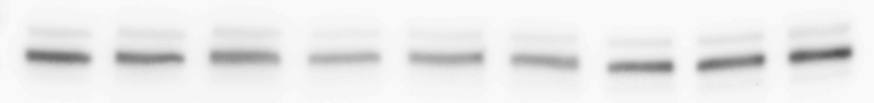

Supplement: Supplementary file 3 — Source data Fig. 1 [file 44318_2024_180_MOESM3_ESM.zip › 1E/S6K1 western cropped.tif]

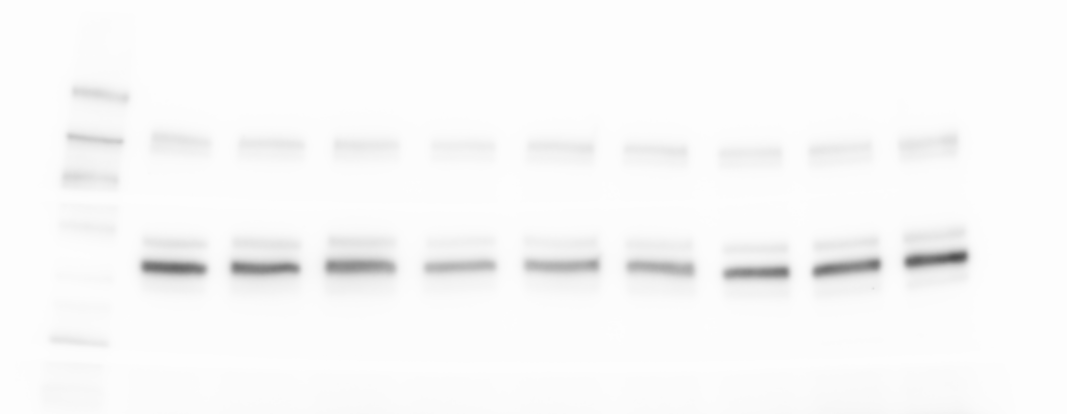

Supplement: Supplementary file 3 — Source data Fig. 1 [file 44318_2024_180_MOESM3_ESM.zip › 1E/S6K1 western.tif]

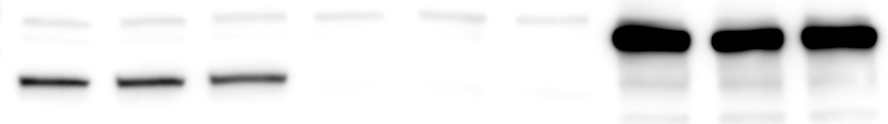

Supplement: Supplementary file 3 — Source data Fig. 1 [file 44318_2024_180_MOESM3_ESM.zip › 1E/TBK1 western cropped.tif]

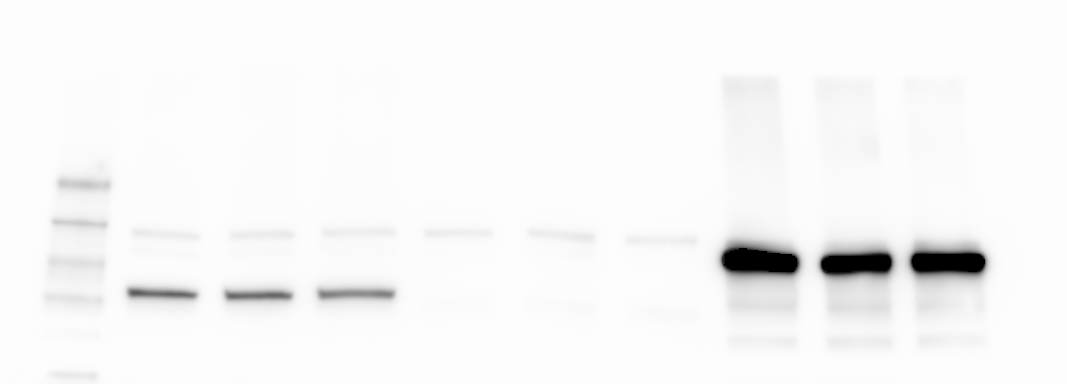

Supplement: Supplementary file 3 — Source data Fig. 1 [file 44318_2024_180_MOESM3_ESM.zip › 1E/TBK1 western.tif]

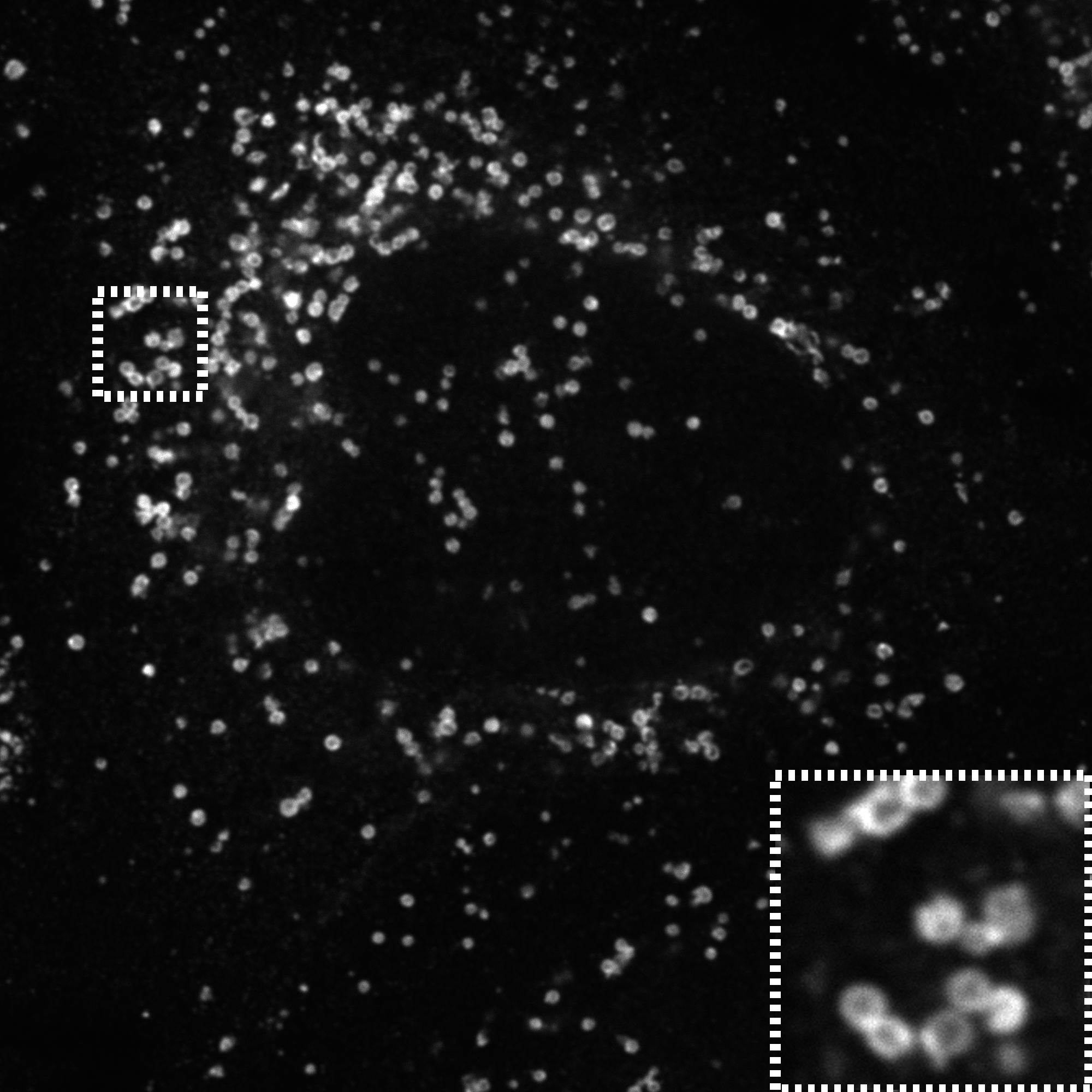

Supplement: Supplementary file 3 — Source data Fig. 1 [file 44318_2024_180_MOESM3_ESM.zip › 1G/TBK1 KO + TBK1-GFP LAMP1 inset.tif]

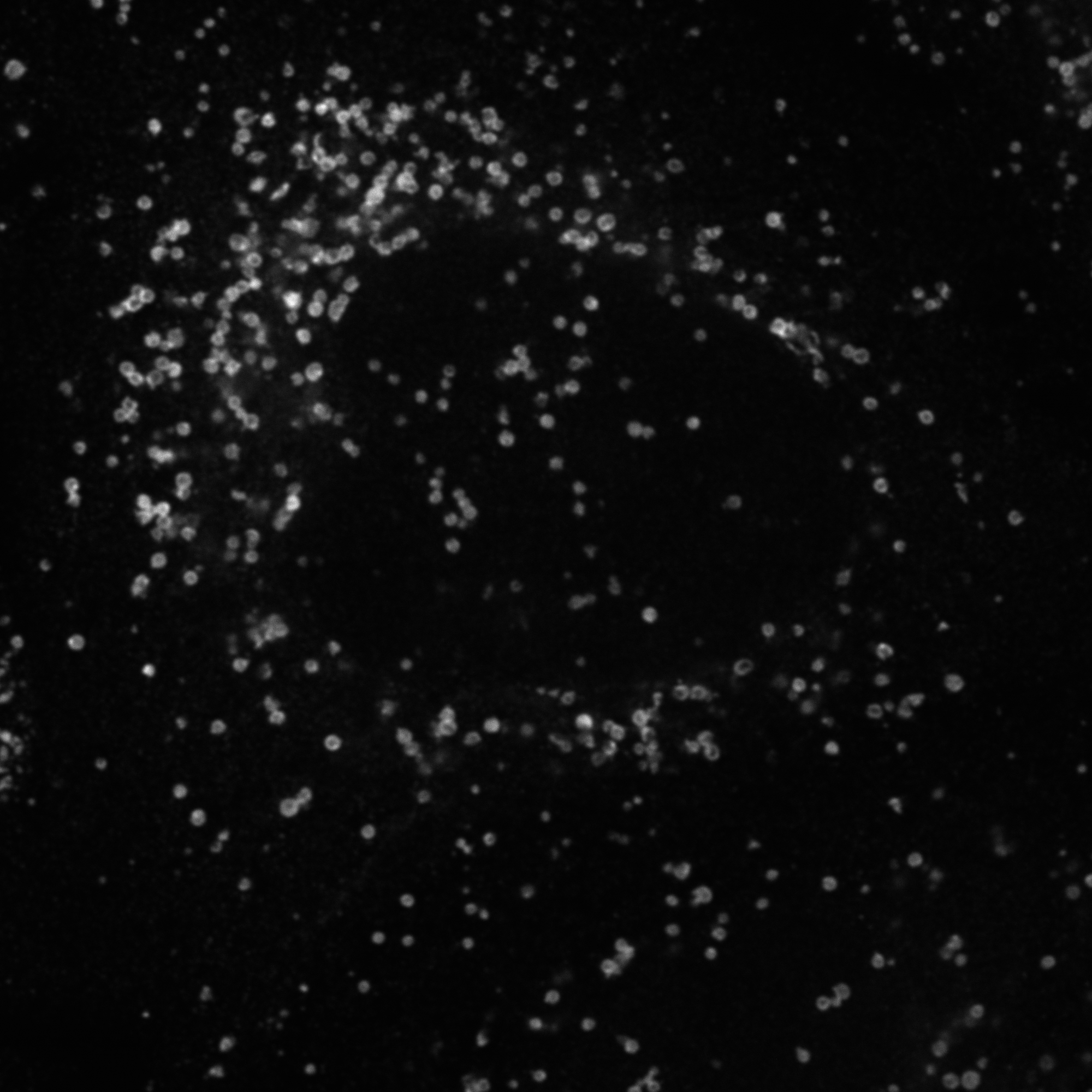

Supplement: Supplementary file 3 — Source data Fig. 1 [file 44318_2024_180_MOESM3_ESM.zip › 1G/TBK1 KO + TBK1-GFP LAMP1.tif]

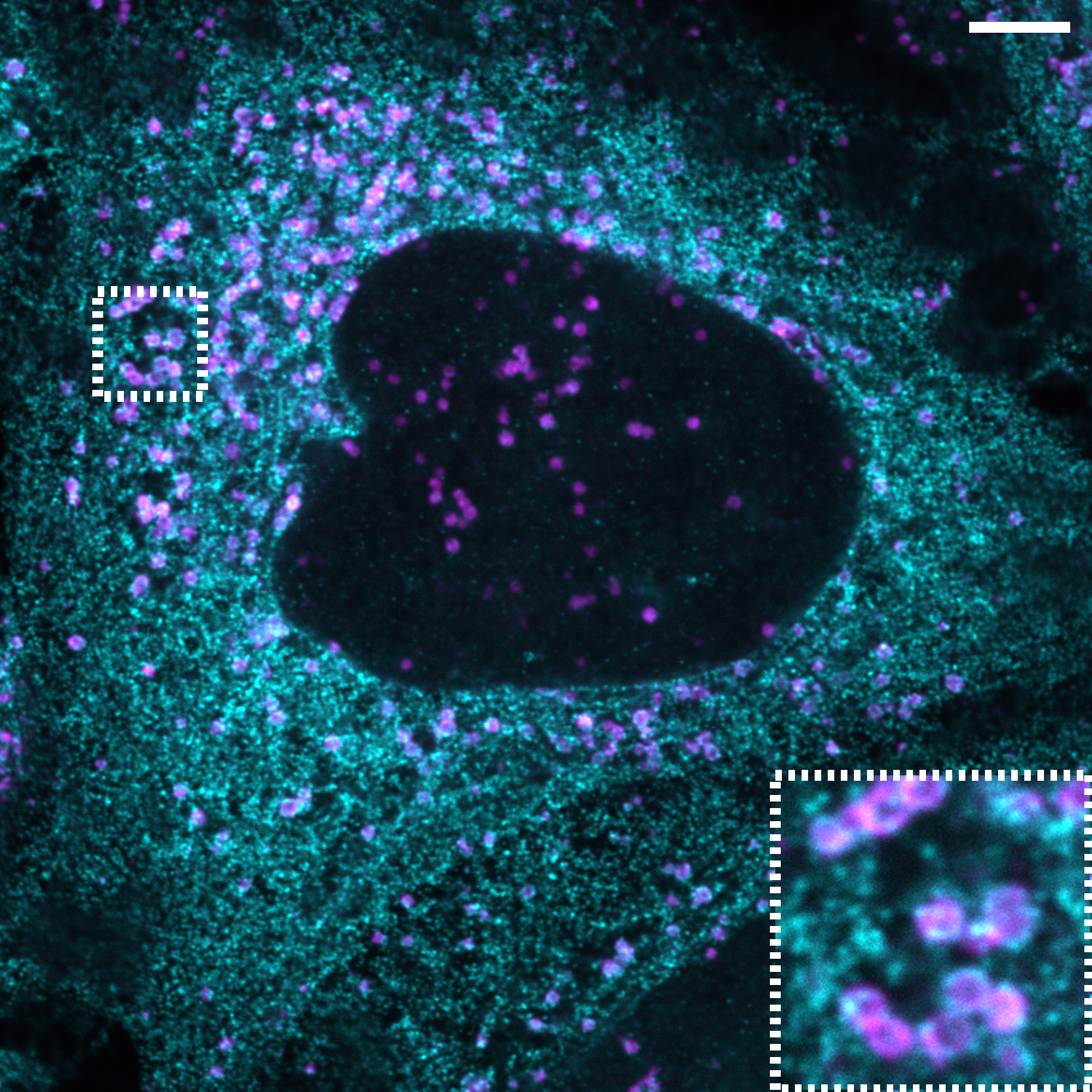

Supplement: Supplementary file 3 — Source data Fig. 1 [file 44318_2024_180_MOESM3_ESM.zip › 1G/TBK1 KO + TBK1-GFP Merge.tif]

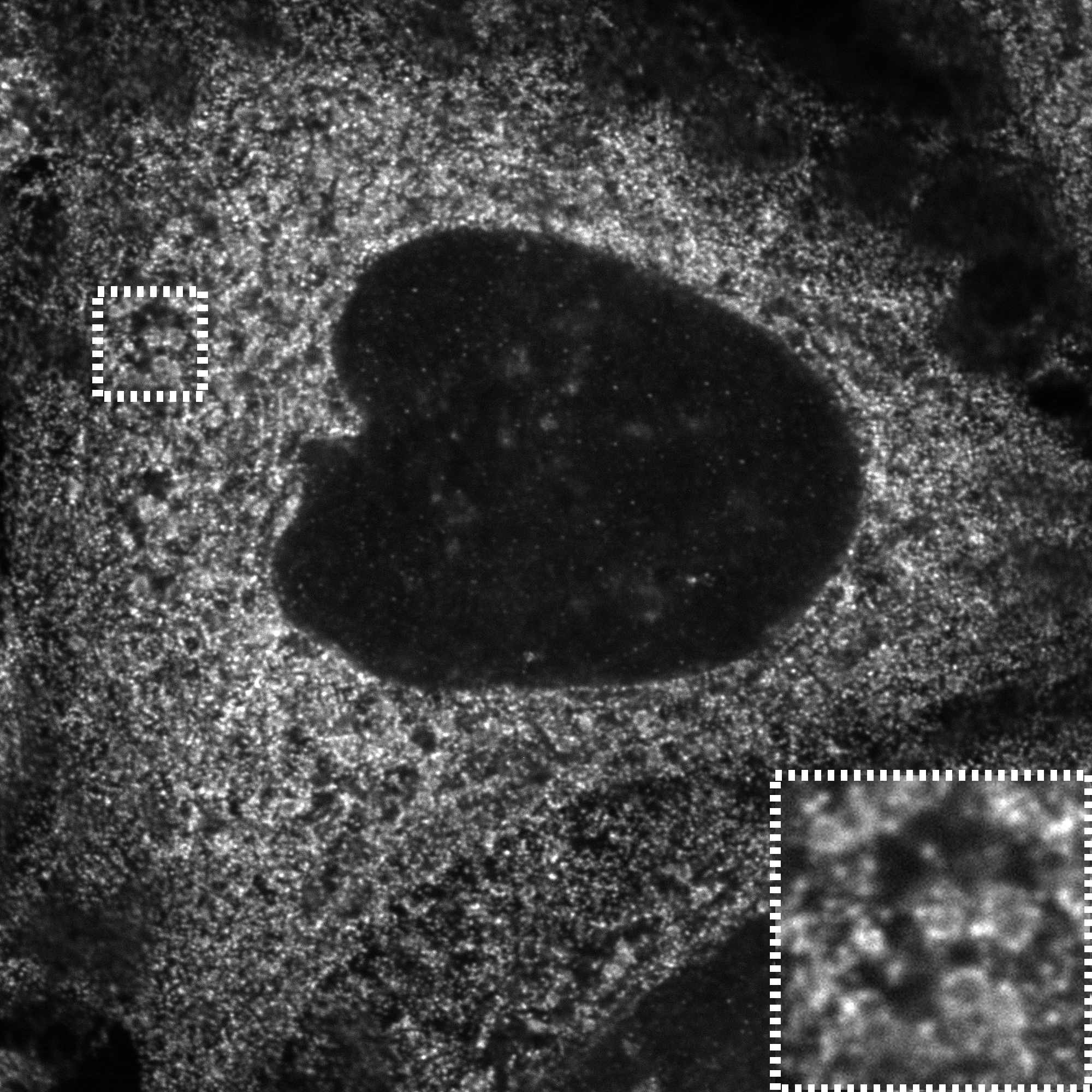

Supplement: Supplementary file 3 — Source data Fig. 1 [file 44318_2024_180_MOESM3_ESM.zip › 1G/TBK1 KO + TBK1-GFP TBK1 inset.tif]

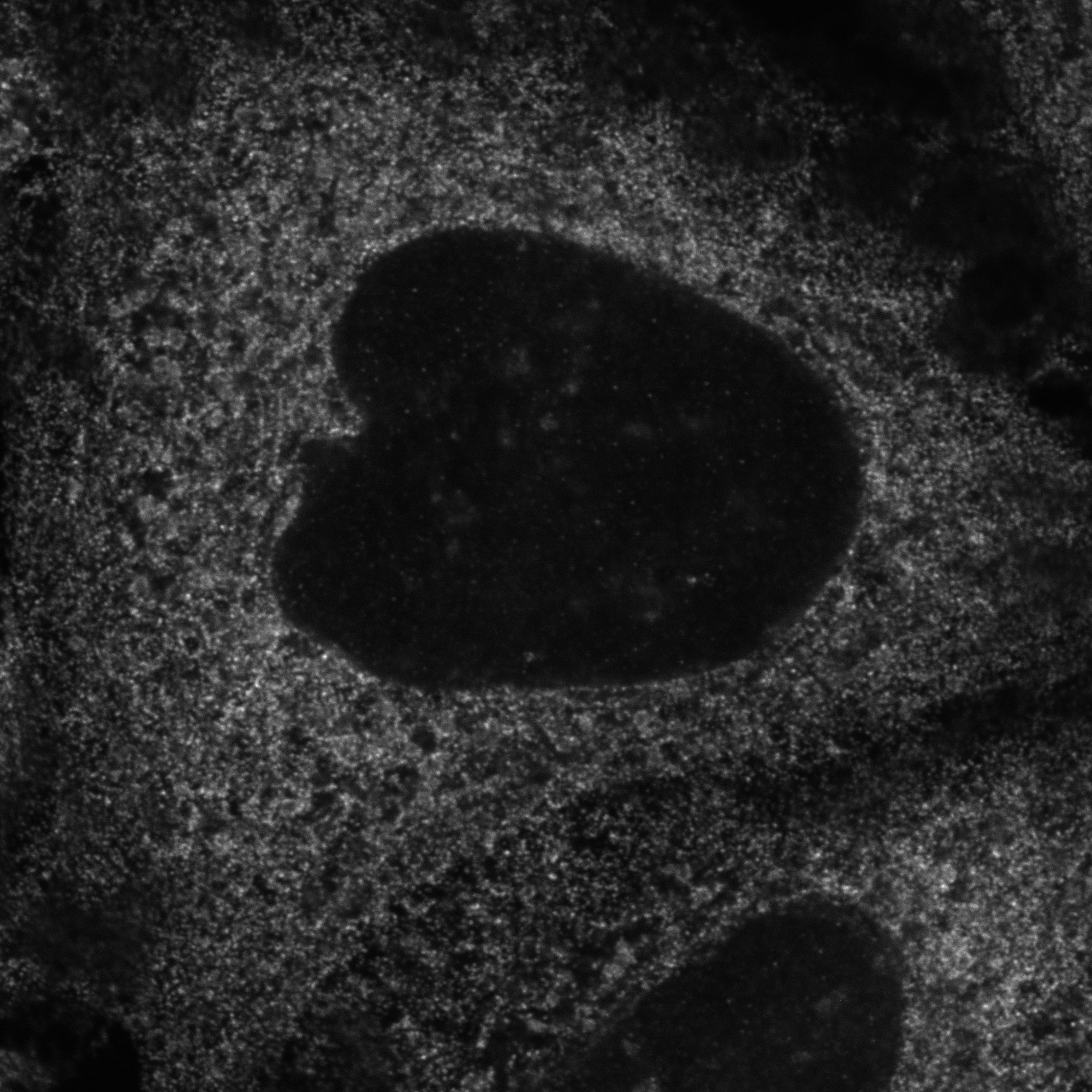

Supplement: Supplementary file 3 — Source data Fig. 1 [file 44318_2024_180_MOESM3_ESM.zip › 1G/TBK1 KO + TBK1-GFP TBK1.tif]

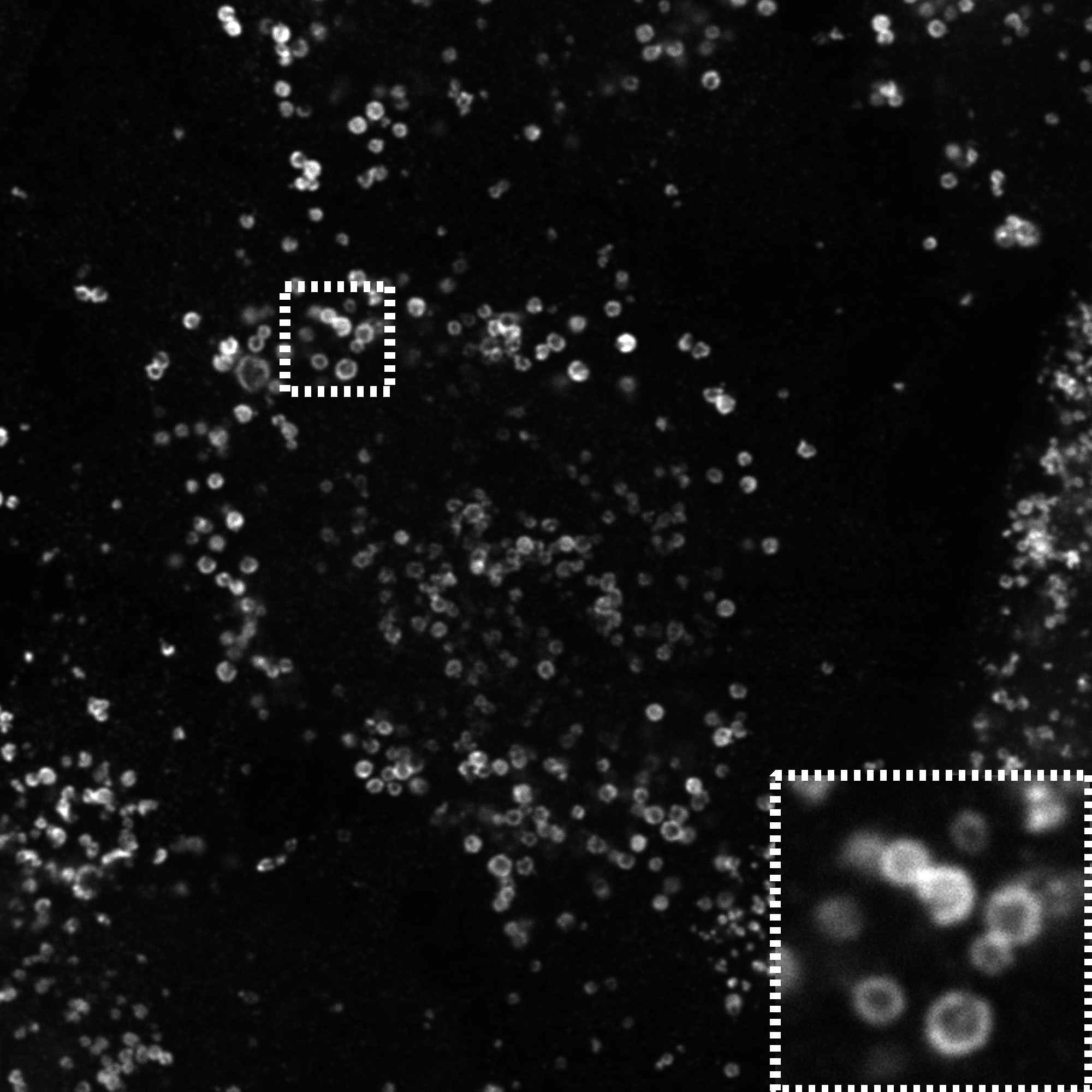

Supplement: Supplementary file 3 — Source data Fig. 1 [file 44318_2024_180_MOESM3_ESM.zip › 1G/TBK1 KO LAMP1 inset.tif]

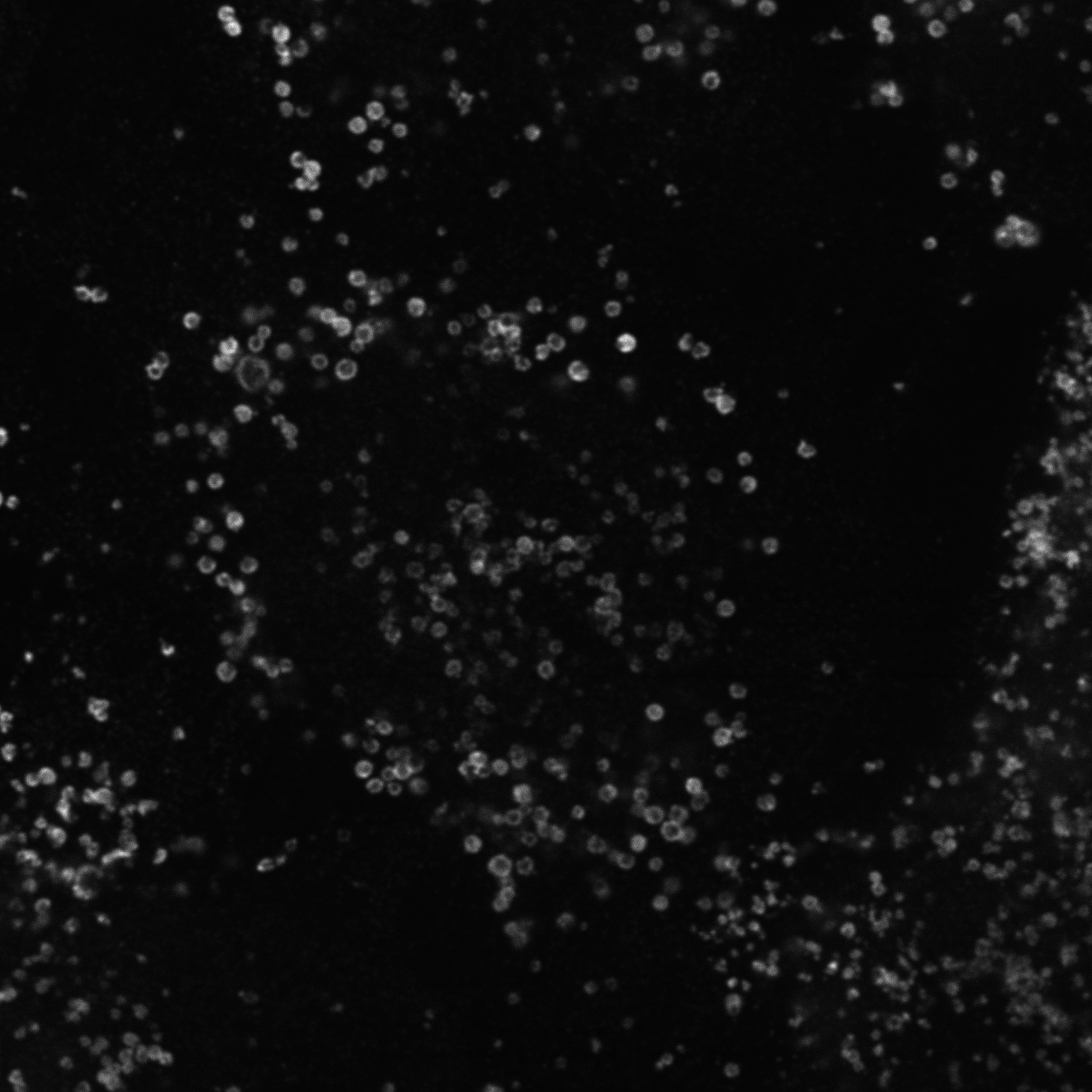

Supplement: Supplementary file 3 — Source data Fig. 1 [file 44318_2024_180_MOESM3_ESM.zip › 1G/TBK1 KO LAMP1.tif]

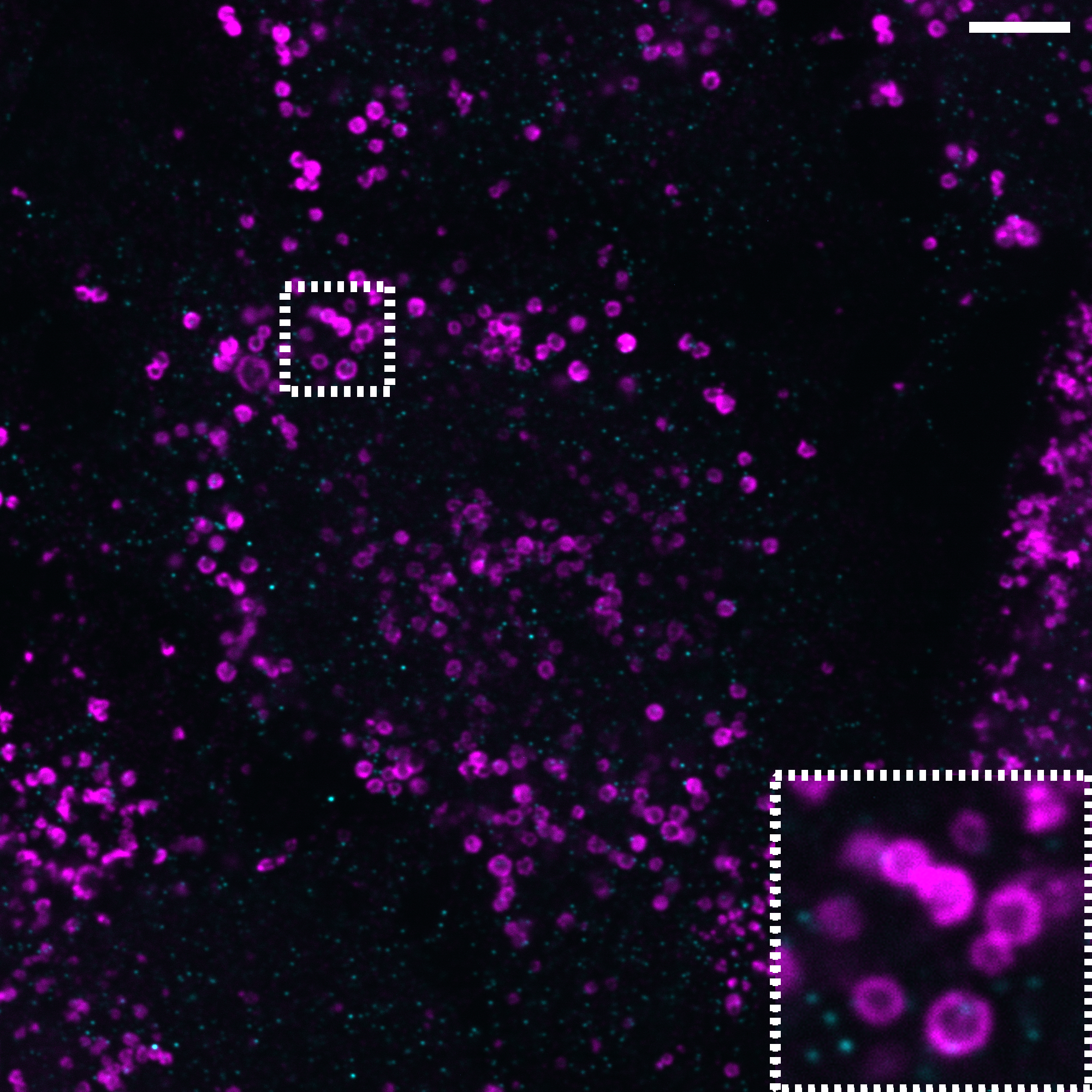

Supplement: Supplementary file 3 — Source data Fig. 1 [file 44318_2024_180_MOESM3_ESM.zip › 1G/TBK1 KO Merge.tif]

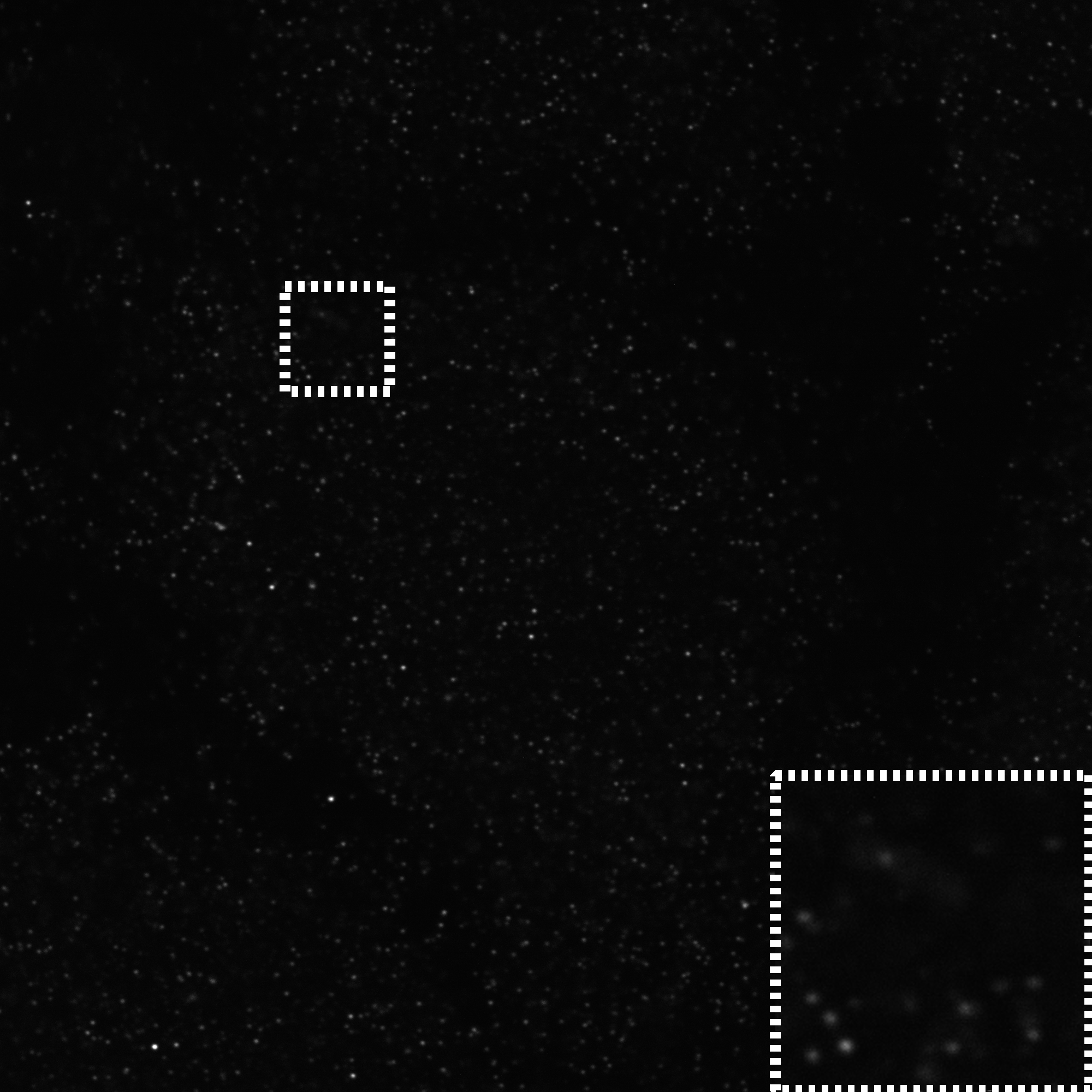

Supplement: Supplementary file 3 — Source data Fig. 1 [file 44318_2024_180_MOESM3_ESM.zip › 1G/TBK1 KO TBK1 inset.tif]

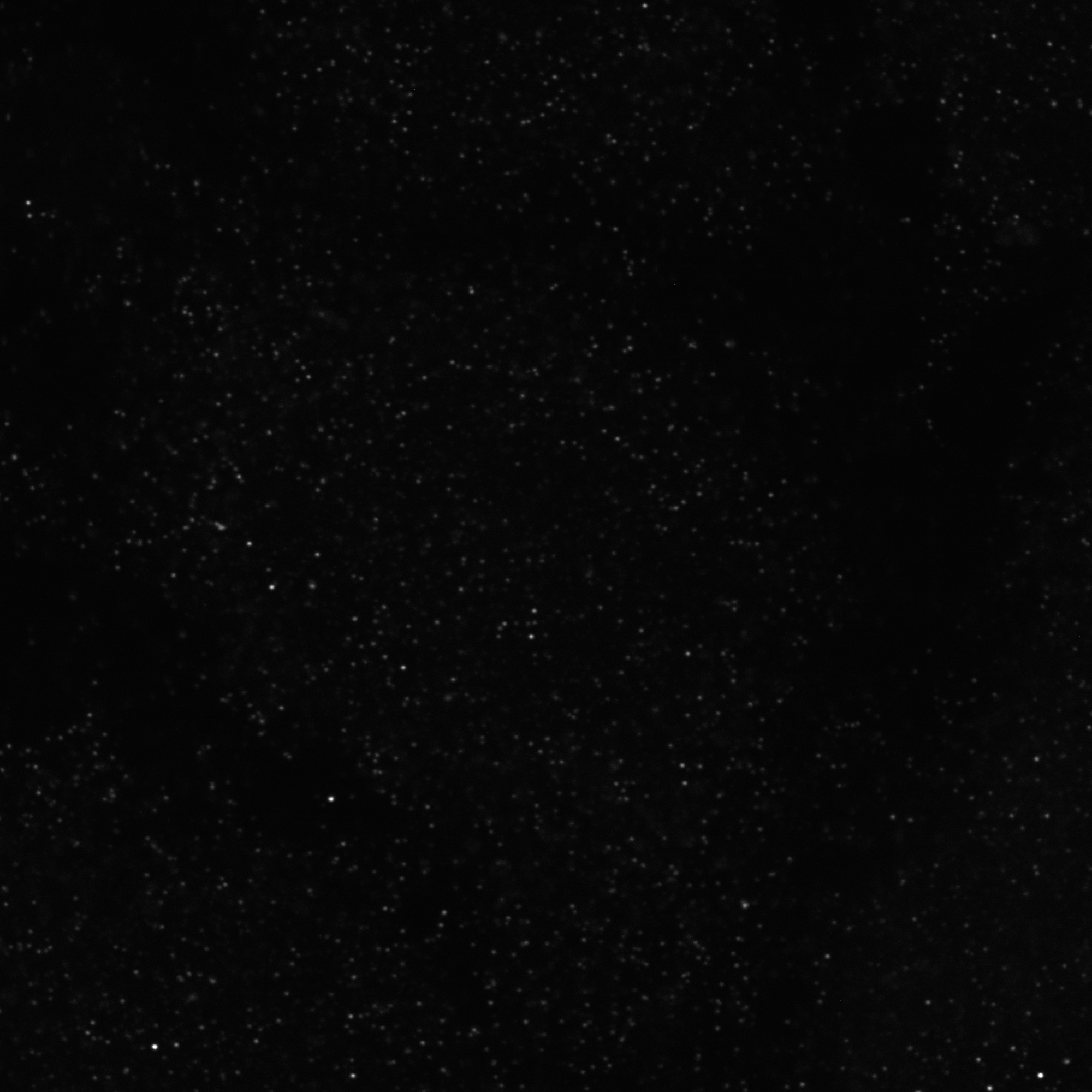

Supplement: Supplementary file 3 — Source data Fig. 1 [file 44318_2024_180_MOESM3_ESM.zip › 1G/TBK1 KO TBK1.tif]

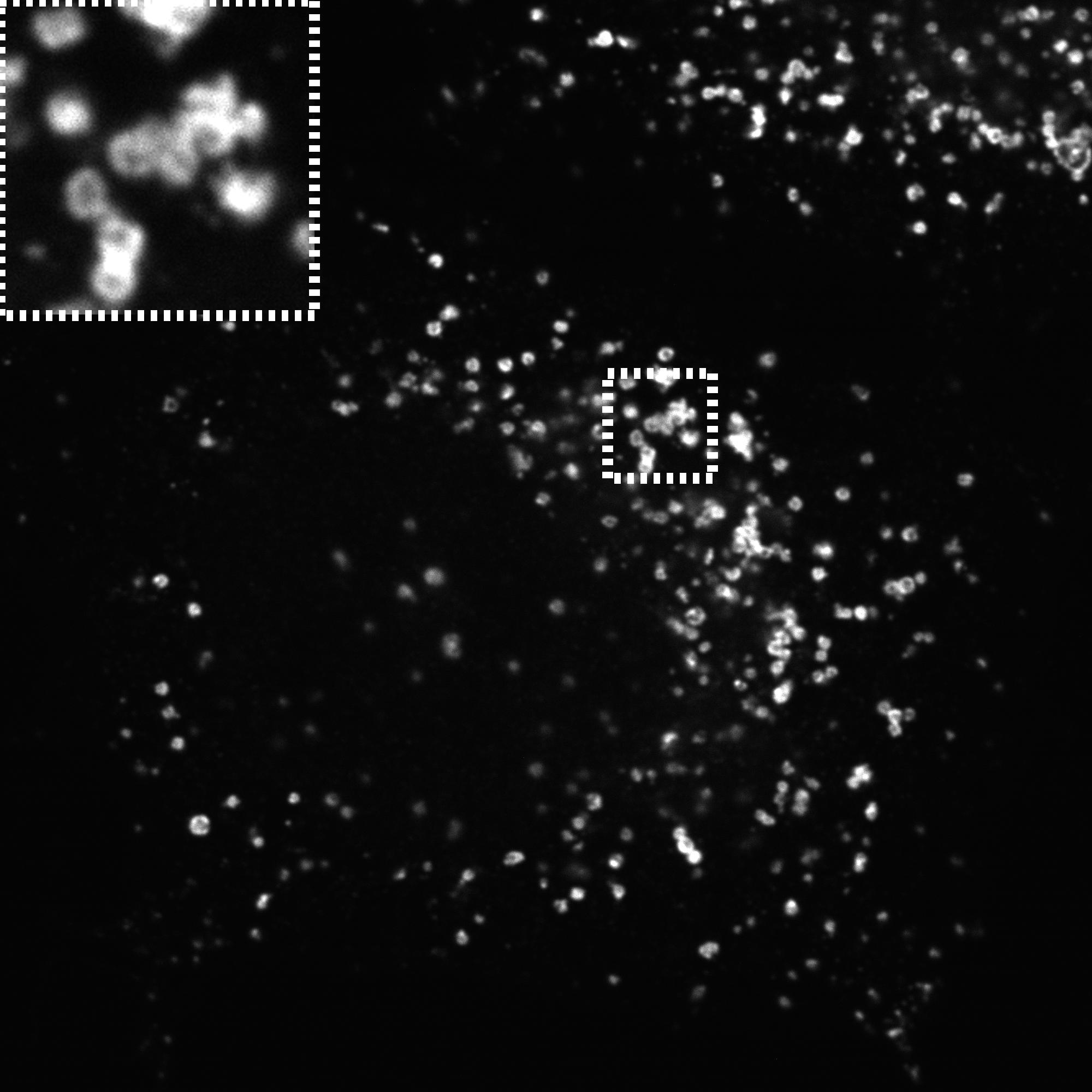

Supplement: Supplementary file 3 — Source data Fig. 1 [file 44318_2024_180_MOESM3_ESM.zip › 1G/WT LAMP1 inset.tif]

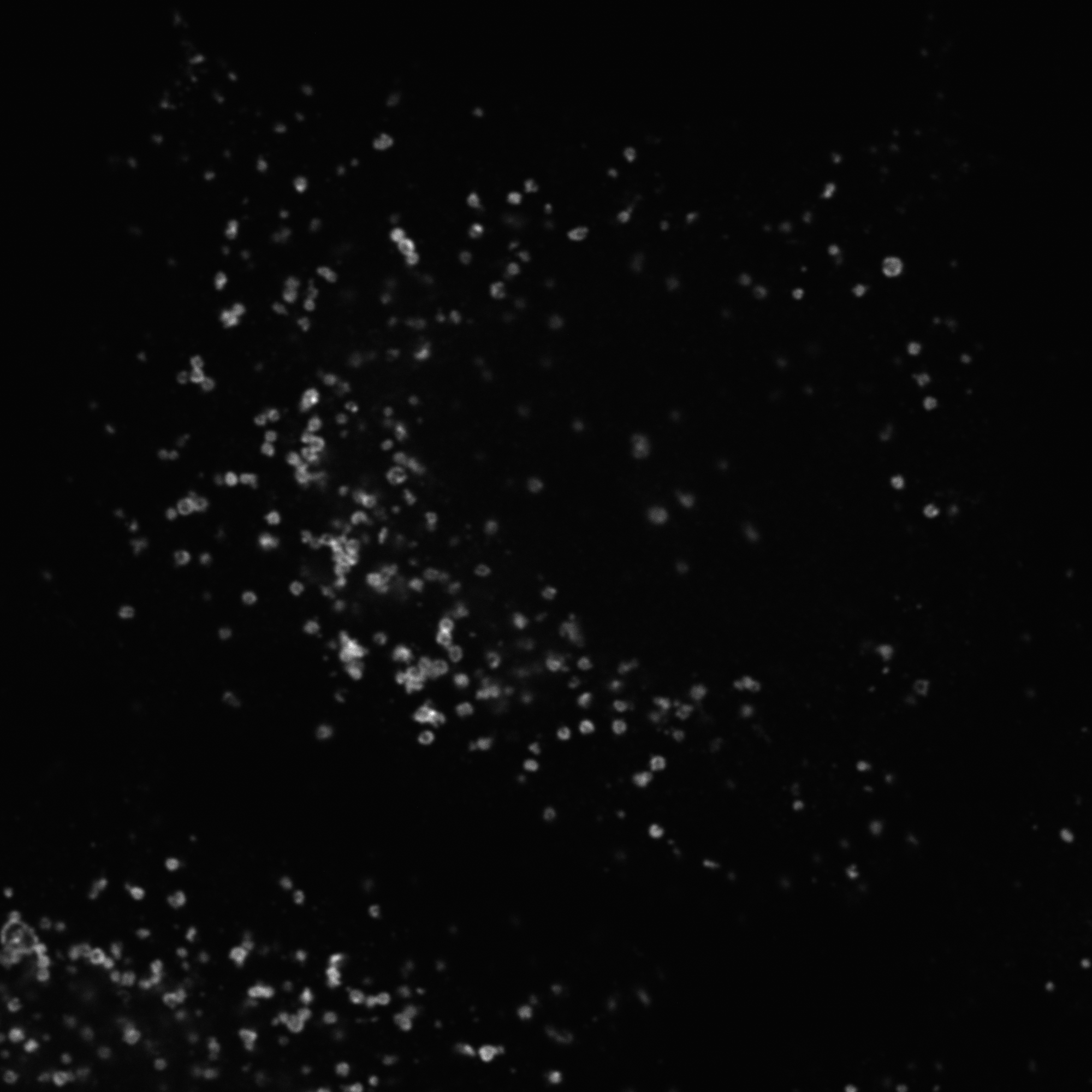

Supplement: Supplementary file 3 — Source data Fig. 1 [file 44318_2024_180_MOESM3_ESM.zip › 1G/WT LAMP1.tif]

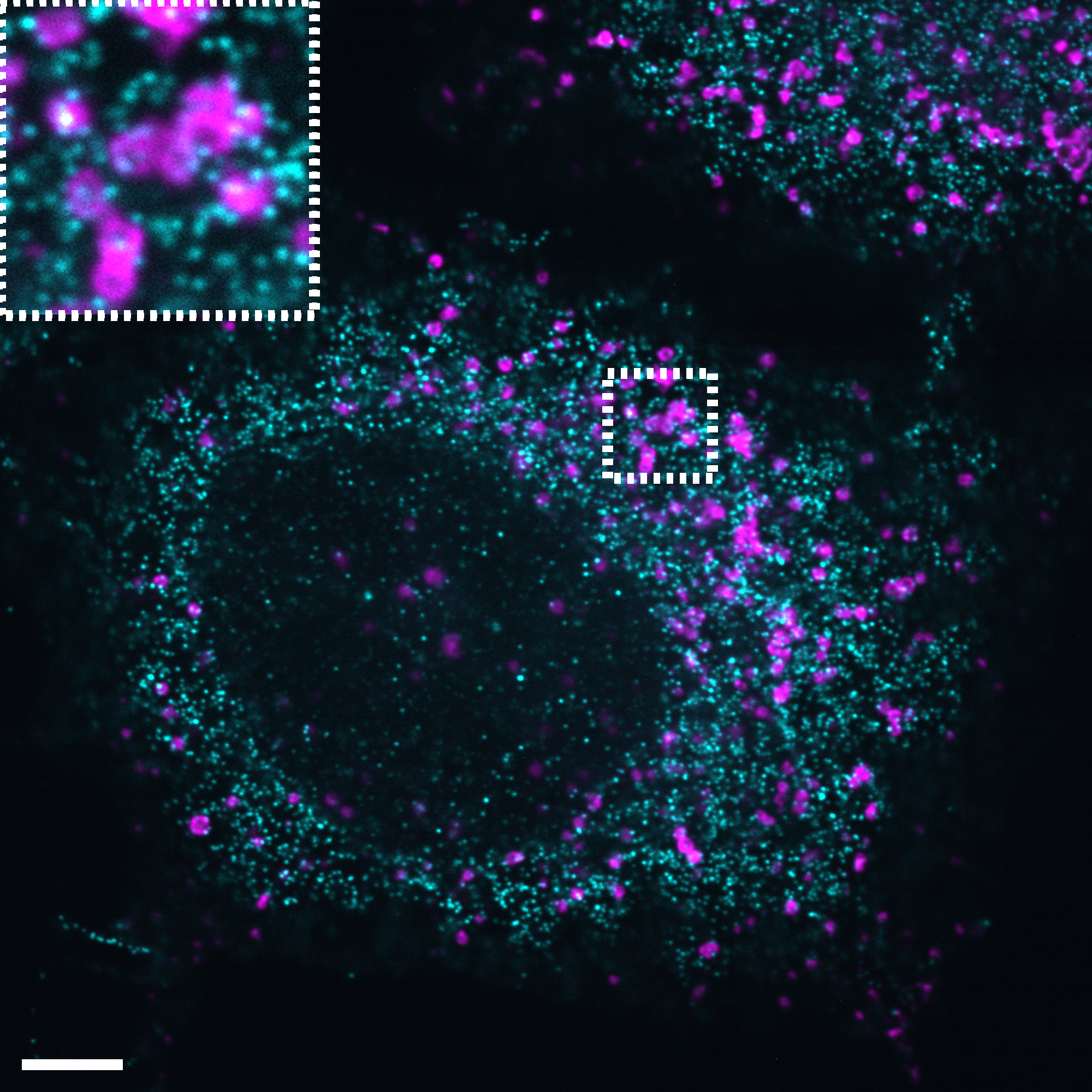

Supplement: Supplementary file 3 — Source data Fig. 1 [file 44318_2024_180_MOESM3_ESM.zip › 1G/WT Merge .tif]

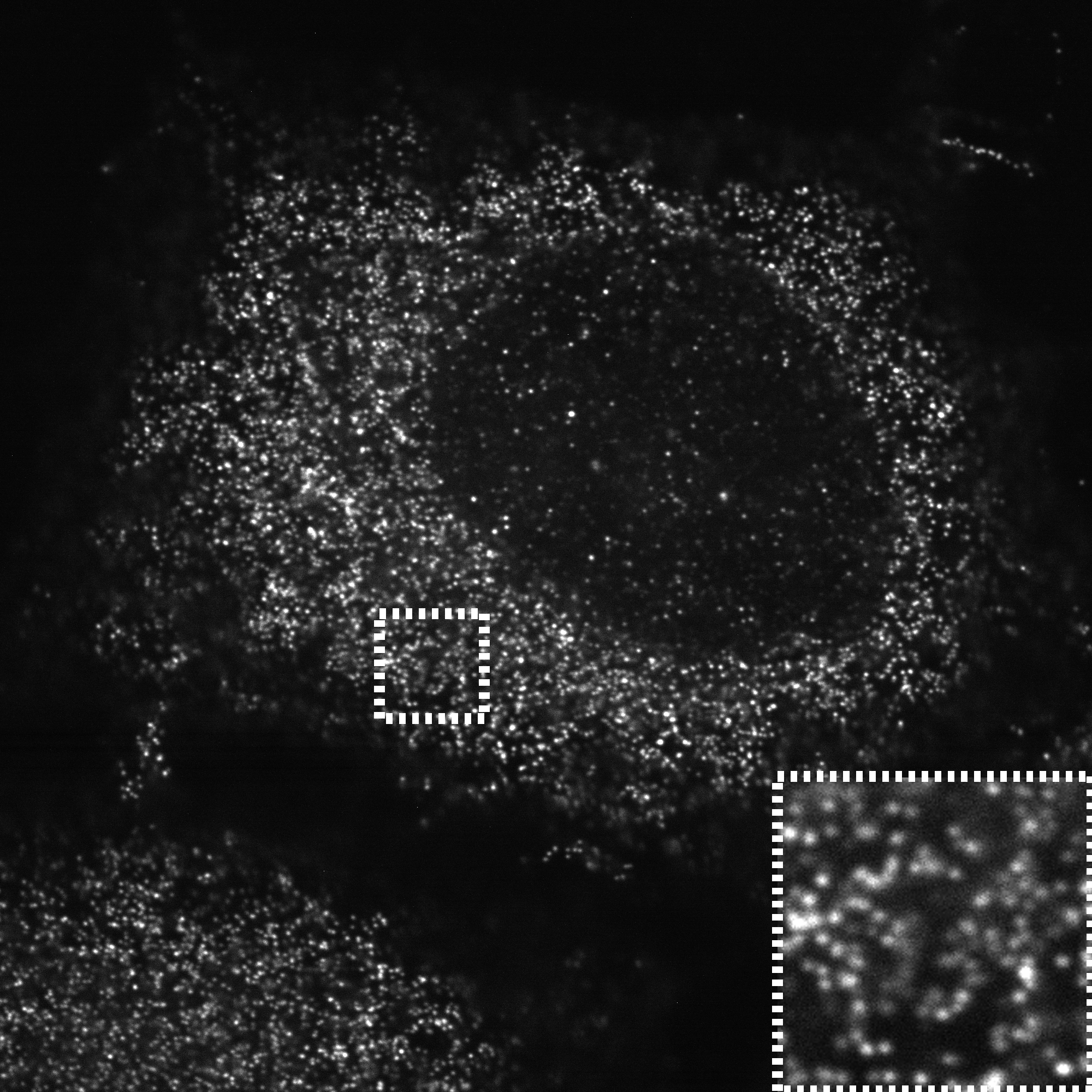

Supplement: Supplementary file 3 — Source data Fig. 1 [file 44318_2024_180_MOESM3_ESM.zip › 1G/WT TBK1 inset.tif]

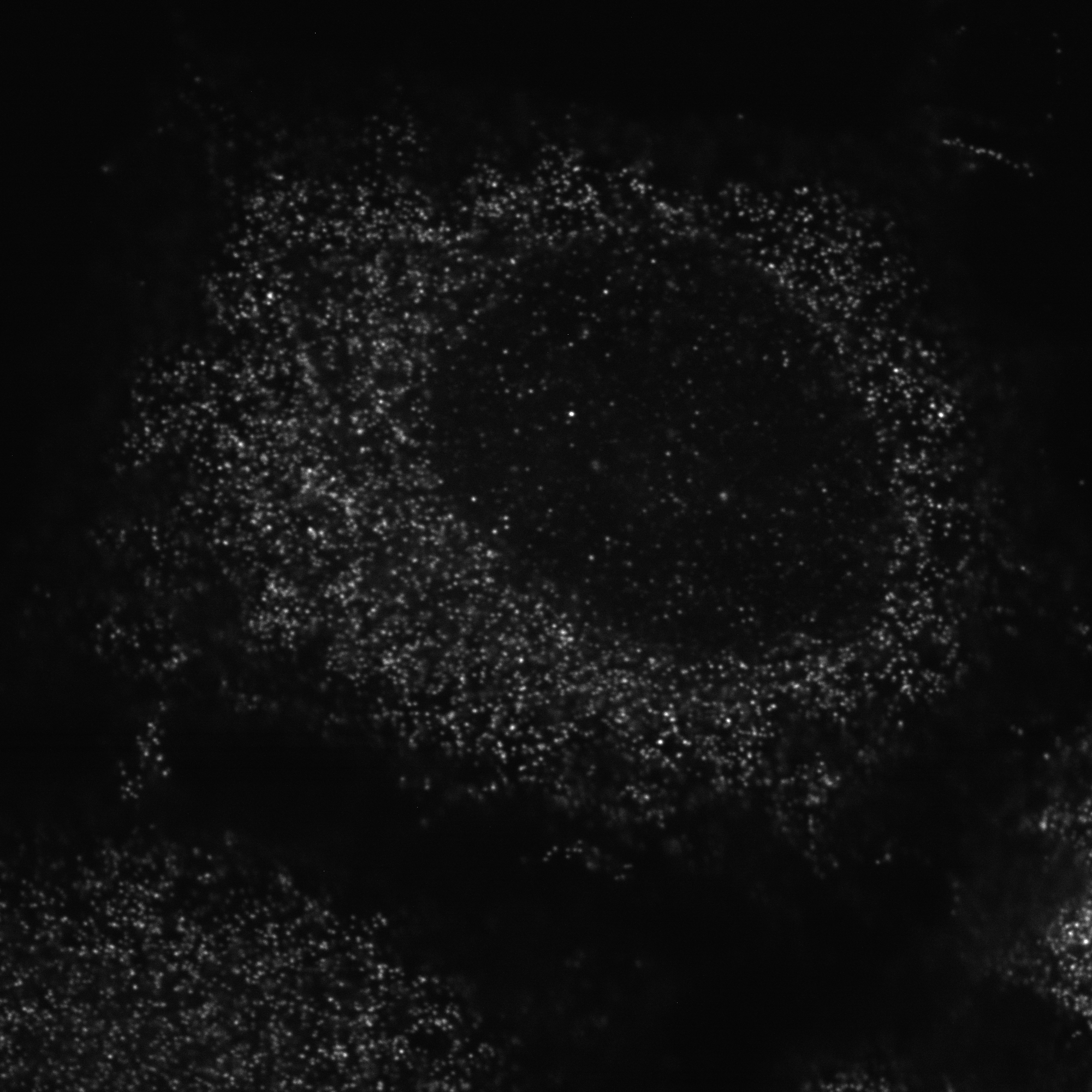

Supplement: Supplementary file 3 — Source data Fig. 1 [file 44318_2024_180_MOESM3_ESM.zip › 1G/WT TBK1.tif]

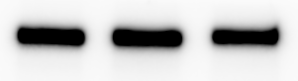

Supplement: Supplementary file 3 — Source data Fig. 1 [file 44318_2024_180_MOESM3_ESM.zip › 1H/GM130 lysates western cropped.tif]

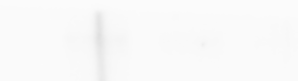

Supplement: Supplementary file 3 — Source data Fig. 1 [file 44318_2024_180_MOESM3_ESM.zip › 1H/GM130 lysosomes western cropped.tif]

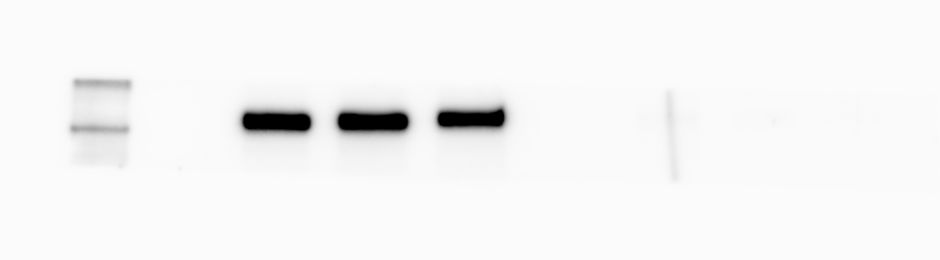

Supplement: Supplementary file 3 — Source data Fig. 1 [file 44318_2024_180_MOESM3_ESM.zip › 1H/GM130 western .tif]

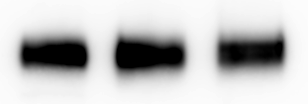

Supplement: Supplementary file 3 — Source data Fig. 1 [file 44318_2024_180_MOESM3_ESM.zip › 1H/LAMP1 lysates western cropped.tif]

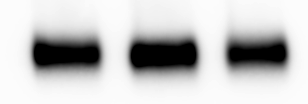

Supplement: Supplementary file 3 — Source data Fig. 1 [file 44318_2024_180_MOESM3_ESM.zip › 1H/LAMP1 lysosomes western cropped.tif]

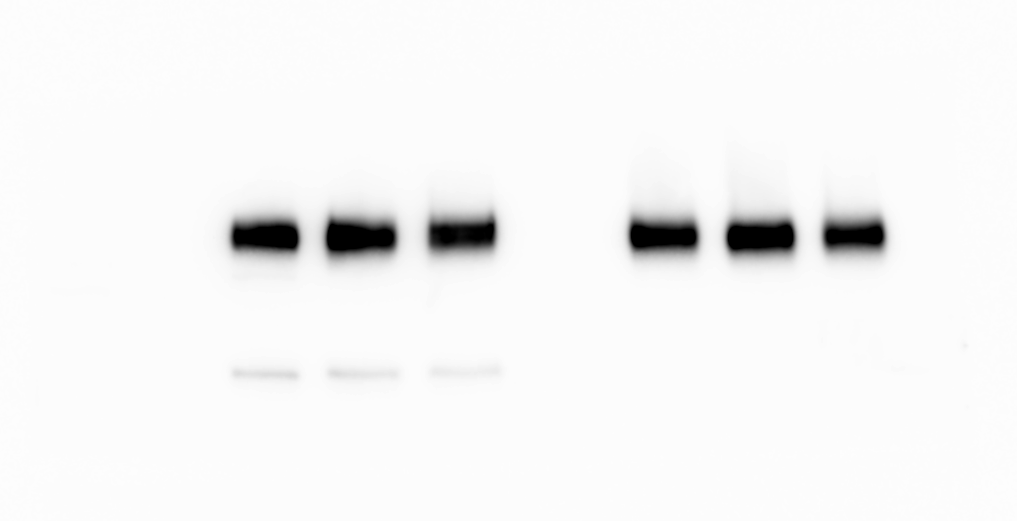

Supplement: Supplementary file 3 — Source data Fig. 1 [file 44318_2024_180_MOESM3_ESM.zip › 1H/LAMP1 western.tif]

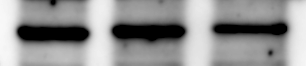

Supplement: Supplementary file 3 — Source data Fig. 1 [file 44318_2024_180_MOESM3_ESM.zip › 1H/PDI lysates western cropped.tif]

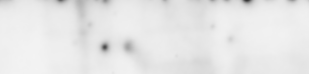

Supplement: Supplementary file 3 — Source data Fig. 1 [file 44318_2024_180_MOESM3_ESM.zip › 1H/PDI lysosomes western cropped.tif]

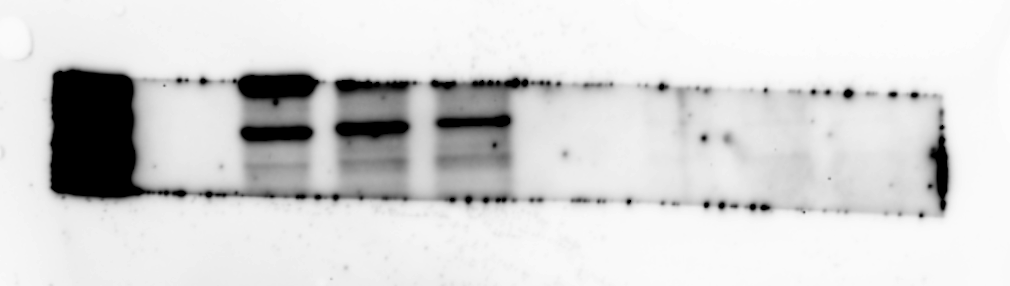

Supplement: Supplementary file 3 — Source data Fig. 1 [file 44318_2024_180_MOESM3_ESM.zip › 1H/PDI western.tif]

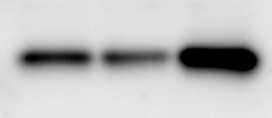

Supplement: Supplementary file 3 — Source data Fig. 1 [file 44318_2024_180_MOESM3_ESM.zip › 1H/pRab7-S72 lysates western cropped.tif]

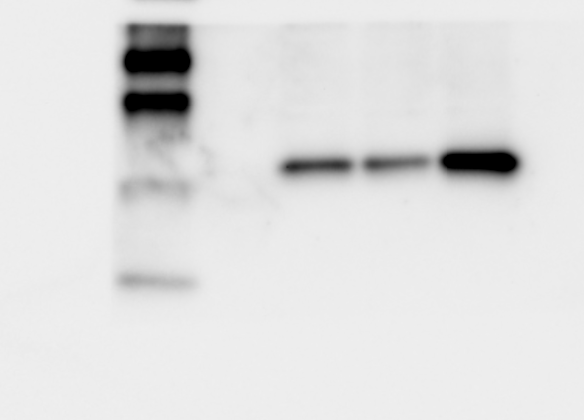

Supplement: Supplementary file 3 — Source data Fig. 1 [file 44318_2024_180_MOESM3_ESM.zip › 1H/pRab7-S72 lysates western.tif]

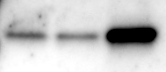

Supplement: Supplementary file 3 — Source data Fig. 1 [file 44318_2024_180_MOESM3_ESM.zip › 1H/pRab7-S72 lysosomes western cropped.tif]

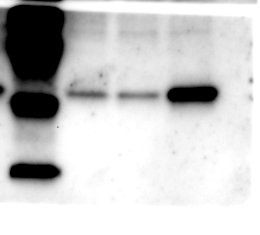

Supplement: Supplementary file 3 — Source data Fig. 1 [file 44318_2024_180_MOESM3_ESM.zip › 1H/pRab7-S72 lysosomes western.tif]

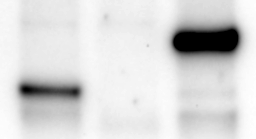

Supplement: Supplementary file 3 — Source data Fig. 1 [file 44318_2024_180_MOESM3_ESM.zip › 1H/pTBK1-lysosomes western cropped.tif]

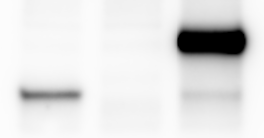

Supplement: Supplementary file 3 — Source data Fig. 1 [file 44318_2024_180_MOESM3_ESM.zip › 1H/pTBK1-S172 lysates western cropped.tif]

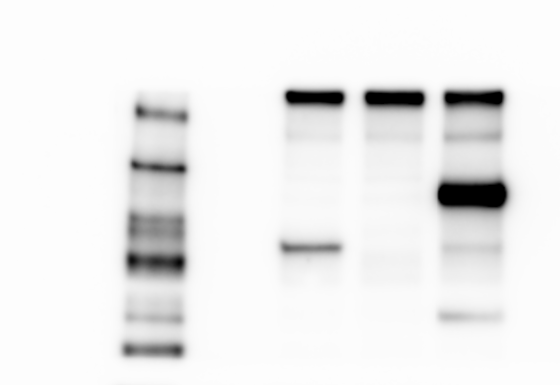

Supplement: Supplementary file 3 — Source data Fig. 1 [file 44318_2024_180_MOESM3_ESM.zip › 1H/pTBK1-S172 lysates western.tif]

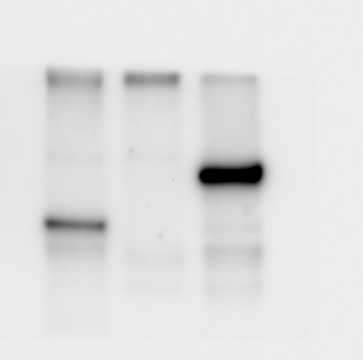

Supplement: Supplementary file 3 — Source data Fig. 1 [file 44318_2024_180_MOESM3_ESM.zip › 1H/pTBK1-S172 lysosomes western.tif]

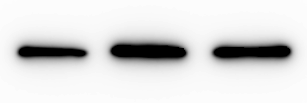

Supplement: Supplementary file 3 — Source data Fig. 1 [file 44318_2024_180_MOESM3_ESM.zip › 1H/Rab7 lysates western cropped.tif]

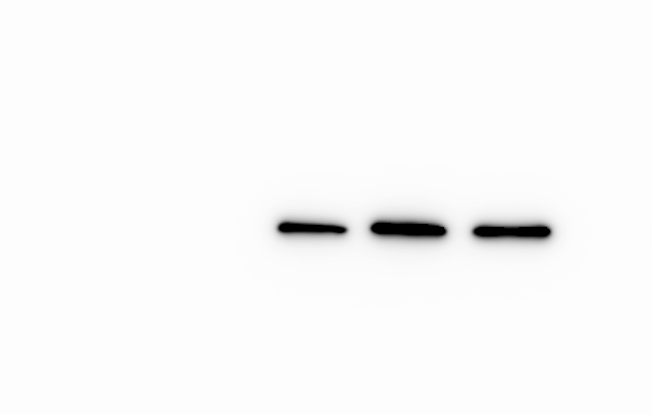

Supplement: Supplementary file 3 — Source data Fig. 1 [file 44318_2024_180_MOESM3_ESM.zip › 1H/Rab7 lysates western.tif]

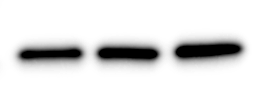

Supplement: Supplementary file 3 — Source data Fig. 1 [file 44318_2024_180_MOESM3_ESM.zip › 1H/Rab7 lysosomes western cropped.tif]

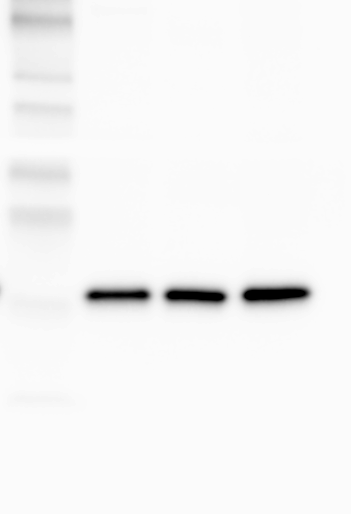

Supplement: Supplementary file 3 — Source data Fig. 1 [file 44318_2024_180_MOESM3_ESM.zip › 1H/Rab7 lysosomes western.tif]

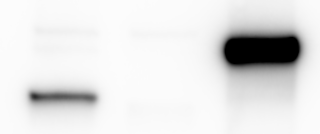

Supplement: Supplementary file 3 — Source data Fig. 1 [file 44318_2024_180_MOESM3_ESM.zip › 1H/TBK1 lysates western cropped.tif]

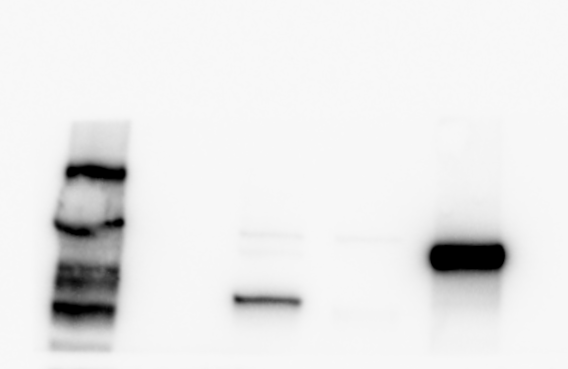

Supplement: Supplementary file 3 — Source data Fig. 1 [file 44318_2024_180_MOESM3_ESM.zip › 1H/TBK1 lysates western.tif]

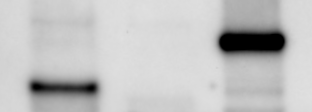

Supplement: Supplementary file 3 — Source data Fig. 1 [file 44318_2024_180_MOESM3_ESM.zip › 1H/TBK1 lysosomes western cropped.tif]

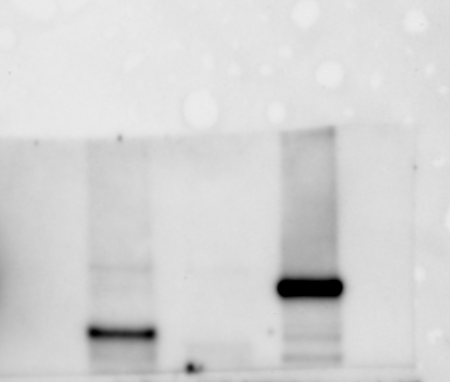

Supplement: Supplementary file 3 — Source data Fig. 1 [file 44318_2024_180_MOESM3_ESM.zip › 1H/TBK1 lysosomes western.tif]

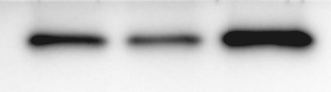

Supplement: Supplementary file 4 — Source data Fig. 2 [file 44318_2024_180_MOESM4_ESM.zip › 2A/pRab7-S72 western cropped.tif]

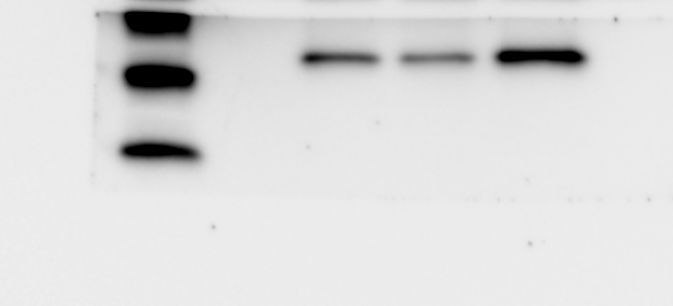

Supplement: Supplementary file 4 — Source data Fig. 2 [file 44318_2024_180_MOESM4_ESM.zip › 2A/pRab7-S72 western.tif]

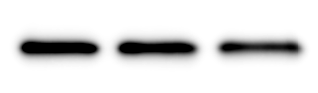

Supplement: Supplementary file 4 — Source data Fig. 2 [file 44318_2024_180_MOESM4_ESM.zip › 2A/Rab7 western cropped.tif]

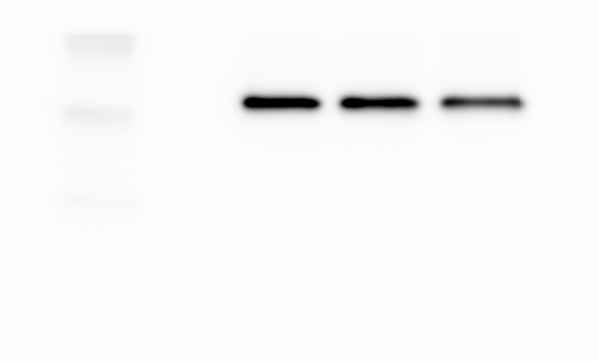

Supplement: Supplementary file 4 — Source data Fig. 2 [file 44318_2024_180_MOESM4_ESM.zip › 2A/Rab7 western.tif]

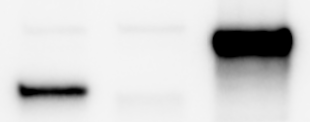

Supplement: Supplementary file 4 — Source data Fig. 2 [file 44318_2024_180_MOESM4_ESM.zip › 2A/TBK1 western cropped.tif]

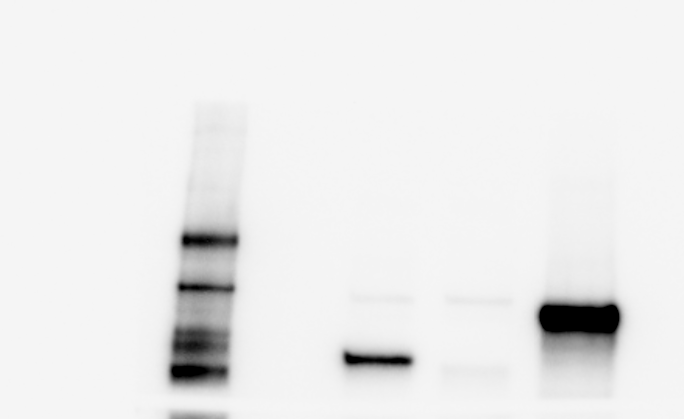

Supplement: Supplementary file 4 — Source data Fig. 2 [file 44318_2024_180_MOESM4_ESM.zip › 2A/TBK1 western.tif]

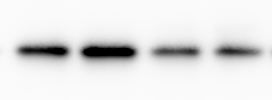

Supplement: Supplementary file 4 — Source data Fig. 2 [file 44318_2024_180_MOESM4_ESM.zip › 2C/pRab7-S72 western cropped.tif]

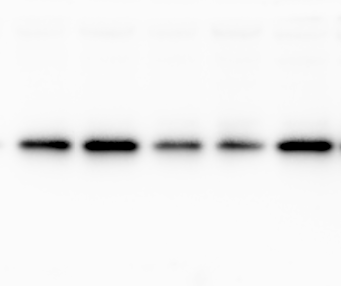

Supplement: Supplementary file 4 — Source data Fig. 2 [file 44318_2024_180_MOESM4_ESM.zip › 2C/pRab7-S72 western.tif]

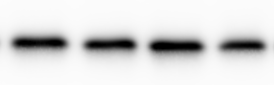

Supplement: Supplementary file 4 — Source data Fig. 2 [file 44318_2024_180_MOESM4_ESM.zip › 2C/Rab7 western cropped.tif]

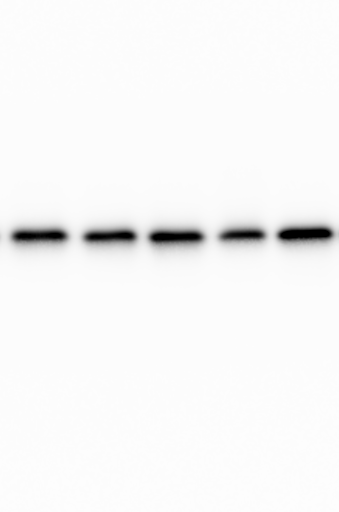

Supplement: Supplementary file 4 — Source data Fig. 2 [file 44318_2024_180_MOESM4_ESM.zip › 2C/Rab7 western.tif]

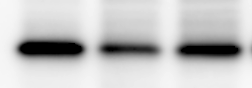

Supplement: Supplementary file 4 — Source data Fig. 2 [file 44318_2024_180_MOESM4_ESM.zip › 2E/pRab7-S72 western cropped.tif]

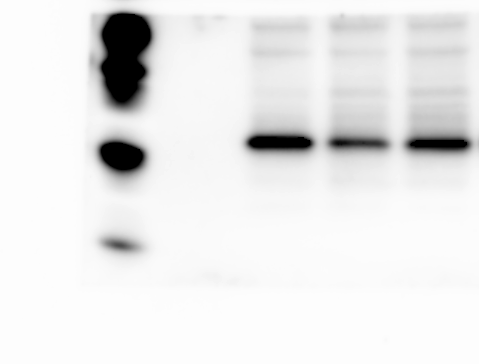

Supplement: Supplementary file 4 — Source data Fig. 2 [file 44318_2024_180_MOESM4_ESM.zip › 2E/pRab7-S72 western.tif]

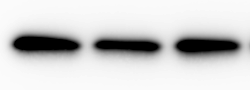

Supplement: Supplementary file 4 — Source data Fig. 2 [file 44318_2024_180_MOESM4_ESM.zip › 2E/Rab7 western cropped.tif]

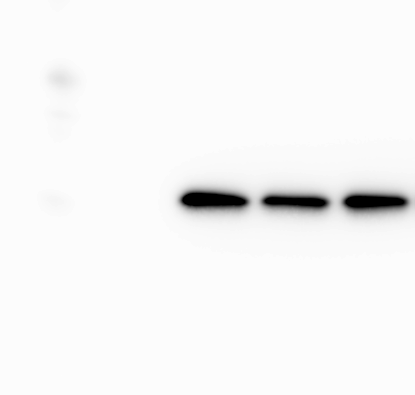

Supplement: Supplementary file 4 — Source data Fig. 2 [file 44318_2024_180_MOESM4_ESM.zip › 2E/Rab7 western.tif]

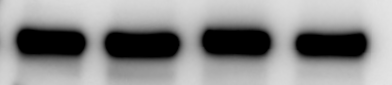

Supplement: Supplementary file 4 — Source data Fig. 2 [file 44318_2024_180_MOESM4_ESM.zip › 2G/GM130 lysates western cropped.tif]

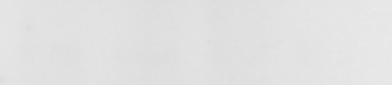

Supplement: Supplementary file 4 — Source data Fig. 2 [file 44318_2024_180_MOESM4_ESM.zip › 2G/GM130 lysosomes western cropped.tif]

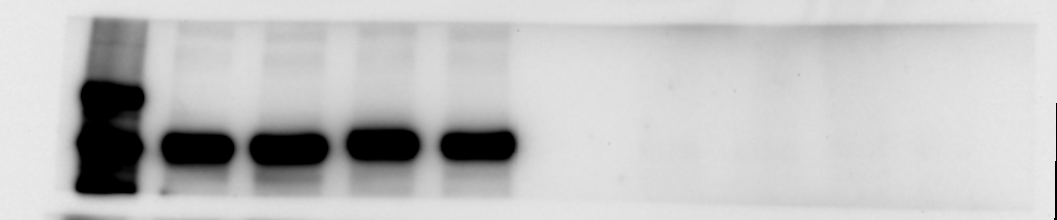

Supplement: Supplementary file 4 — Source data Fig. 2 [file 44318_2024_180_MOESM4_ESM.zip › 2G/GM130 western.tif]

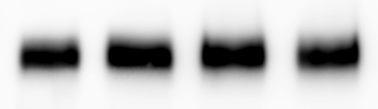

Supplement: Supplementary file 4 — Source data Fig. 2 [file 44318_2024_180_MOESM4_ESM.zip › 2G/LAMP1 lysates western cropped.tif]

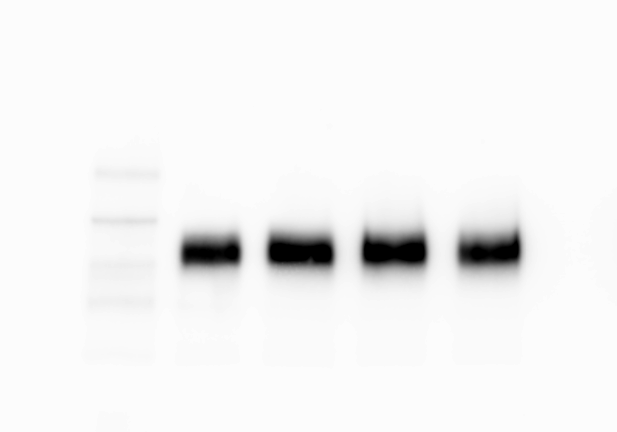

Supplement: Supplementary file 4 — Source data Fig. 2 [file 44318_2024_180_MOESM4_ESM.zip › 2G/LAMP1 lysates western.tif]

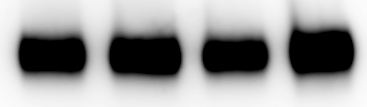

Supplement: Supplementary file 4 — Source data Fig. 2 [file 44318_2024_180_MOESM4_ESM.zip › 2G/LAMP1 lysosomes western cropped.tif]

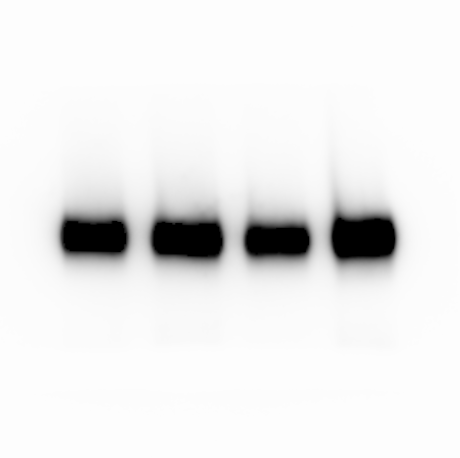

Supplement: Supplementary file 4 — Source data Fig. 2 [file 44318_2024_180_MOESM4_ESM.zip › 2G/LAMP1 lysosomes western.tif]

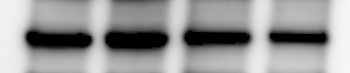

Supplement: Supplementary file 4 — Source data Fig. 2 [file 44318_2024_180_MOESM4_ESM.zip › 2G/PDI lysates western cropped.tif]

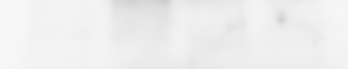

Supplement: Supplementary file 4 — Source data Fig. 2 [file 44318_2024_180_MOESM4_ESM.zip › 2G/PDI lysosomes western cropped.tif]

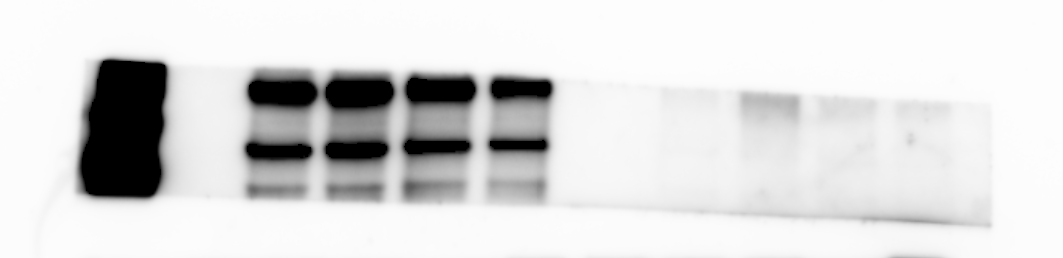

Supplement: Supplementary file 4 — Source data Fig. 2 [file 44318_2024_180_MOESM4_ESM.zip › 2G/PDI western.tif]

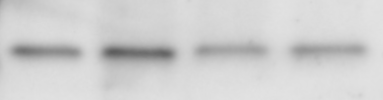

Supplement: Supplementary file 4 — Source data Fig. 2 [file 44318_2024_180_MOESM4_ESM.zip › 2G/pRab7-S72 lysates western cropped.tif]

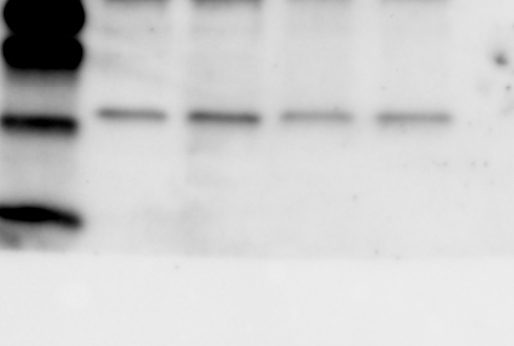

Supplement: Supplementary file 4 — Source data Fig. 2 [file 44318_2024_180_MOESM4_ESM.zip › 2G/pRab7-S72 lysates western.tif]

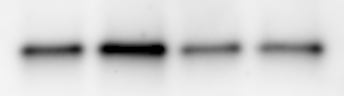

Supplement: Supplementary file 4 — Source data Fig. 2 [file 44318_2024_180_MOESM4_ESM.zip › 2G/pRab7-S72 lysosomes western cropped.tif]

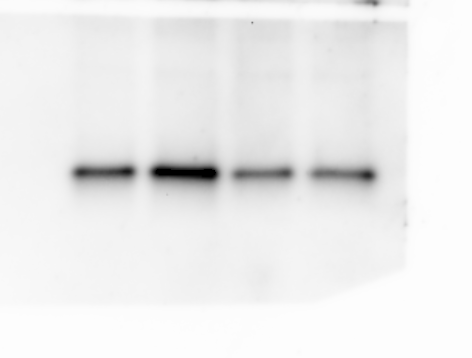

Supplement: Supplementary file 4 — Source data Fig. 2 [file 44318_2024_180_MOESM4_ESM.zip › 2G/pRab7-S72 lysosomes western.tif]

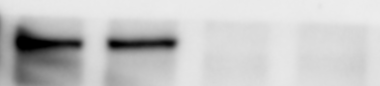

Supplement: Supplementary file 4 — Source data Fig. 2 [file 44318_2024_180_MOESM4_ESM.zip › 2G/pTBK1-S172 lysates western cropped.tif]

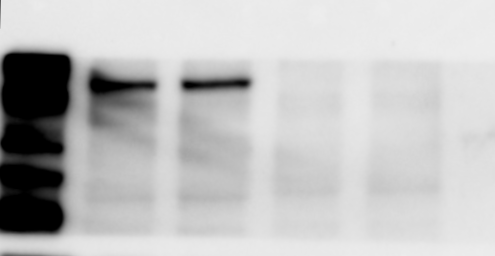

Supplement: Supplementary file 4 — Source data Fig. 2 [file 44318_2024_180_MOESM4_ESM.zip › 2G/pTBK1-S172 lysates western.tif]

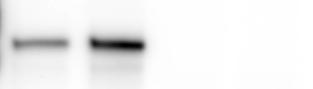

Supplement: Supplementary file 4 — Source data Fig. 2 [file 44318_2024_180_MOESM4_ESM.zip › 2G/pTBK1-S172 lysosomes western cropped.tif]

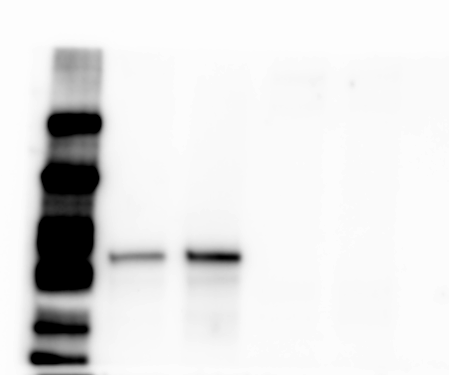

Supplement: Supplementary file 4 — Source data Fig. 2 [file 44318_2024_180_MOESM4_ESM.zip › 2G/pTBK1-S172 lysosomes western.tif]

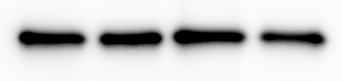

Supplement: Supplementary file 4 — Source data Fig. 2 [file 44318_2024_180_MOESM4_ESM.zip › 2G/Rab7 lysates western cropped.tif]

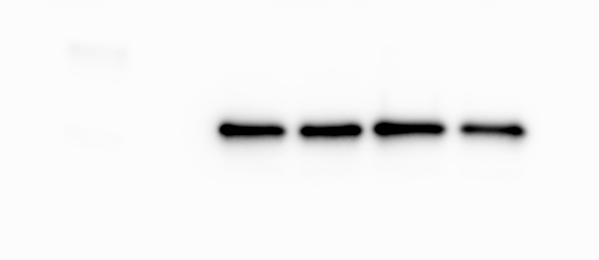

Supplement: Supplementary file 4 — Source data Fig. 2 [file 44318_2024_180_MOESM4_ESM.zip › 2G/Rab7 lysates western.tif]

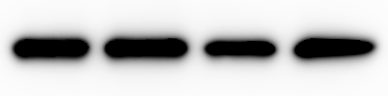

Supplement: Supplementary file 4 — Source data Fig. 2 [file 44318_2024_180_MOESM4_ESM.zip › 2G/Rab7 lysosomes western cropped.tif]

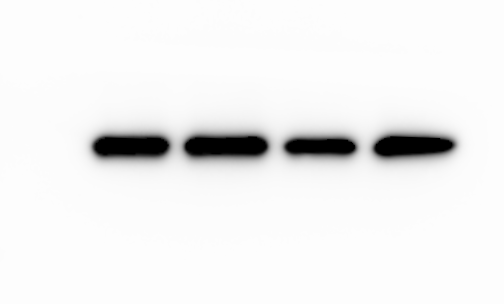

Supplement: Supplementary file 4 — Source data Fig. 2 [file 44318_2024_180_MOESM4_ESM.zip › 2G/Rab7 lysososomes western.tif]

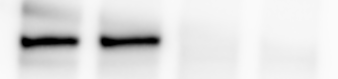

Supplement: Supplementary file 4 — Source data Fig. 2 [file 44318_2024_180_MOESM4_ESM.zip › 2G/TBK1 lysates western cropped.tif]

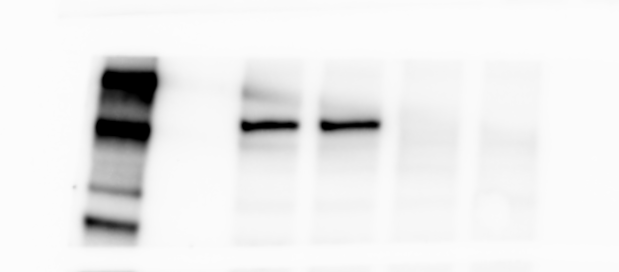

Supplement: Supplementary file 4 — Source data Fig. 2 [file 44318_2024_180_MOESM4_ESM.zip › 2G/TBK1 lysates western.tif]

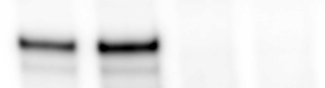

Supplement: Supplementary file 4 — Source data Fig. 2 [file 44318_2024_180_MOESM4_ESM.zip › 2G/TBK1 lysosomes western cropped.tif]

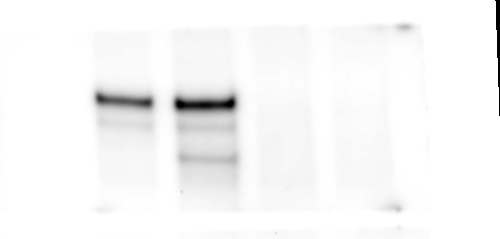

Supplement: Supplementary file 4 — Source data Fig. 2 [file 44318_2024_180_MOESM4_ESM.zip › 2G/TBK1 lysosomes western.tif]

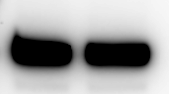

Supplement: Supplementary file 5 — Source data Fig. 3 [file 44318_2024_180_MOESM5_ESM.zip › 3A/GM130 lysates western cropped.tif]

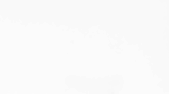

Supplement: Supplementary file 5 — Source data Fig. 3 [file 44318_2024_180_MOESM5_ESM.zip › 3A/GM130 lysosomes western cropped.tif]

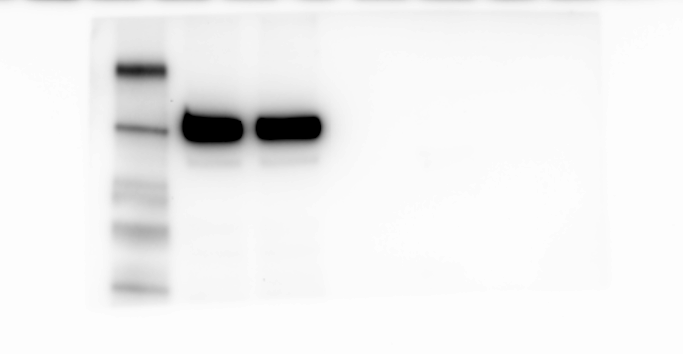

Supplement: Supplementary file 5 — Source data Fig. 3 [file 44318_2024_180_MOESM5_ESM.zip › 3A/GM130 western.tif]

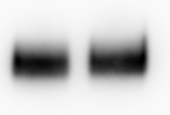

Supplement: Supplementary file 5 — Source data Fig. 3 [file 44318_2024_180_MOESM5_ESM.zip › 3A/LAMP1 lysates western cropped.tif]
