# Supplementary figures and images for: Lysosomal TBK1 responds to amino acid availability to relieve Rab7-dependent mTORC1 inhibition (part 2 of 3)
Source: EMBO J. 2024 Aug 5;43(18):7. doi: 10.1038/s44318-024-00180-8 (PMC11405869; doi:10.1038/s44318-024-00180-8)

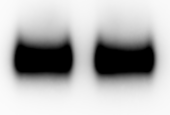

Supplement: Supplementary file 5 — Source data Fig. 3 [file 44318_2024_180_MOESM5_ESM.zip › 3A/LAMP1 lysosomes western cropped.tif]

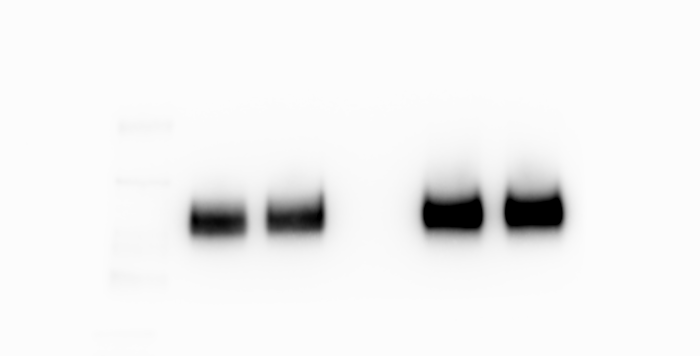

Supplement: Supplementary file 5 — Source data Fig. 3 [file 44318_2024_180_MOESM5_ESM.zip › 3A/LAMP1 western.tif]

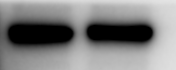

Supplement: Supplementary file 5 — Source data Fig. 3 [file 44318_2024_180_MOESM5_ESM.zip › 3A/PDI lysates western cropped.tif]

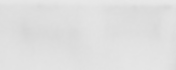

Supplement: Supplementary file 5 — Source data Fig. 3 [file 44318_2024_180_MOESM5_ESM.zip › 3A/PDI lysosomes western cropped.tif]

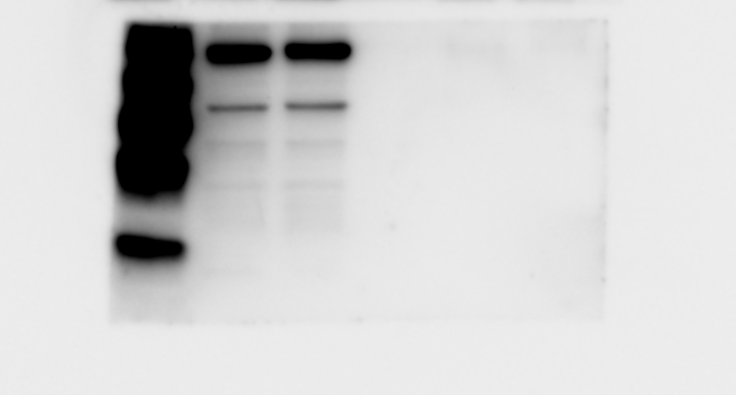

Supplement: Supplementary file 5 — Source data Fig. 3 [file 44318_2024_180_MOESM5_ESM.zip › 3A/PDI western.tif]

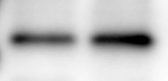

Supplement: Supplementary file 5 — Source data Fig. 3 [file 44318_2024_180_MOESM5_ESM.zip › 3A/pRab7-S72 lysates western cropped.tif]

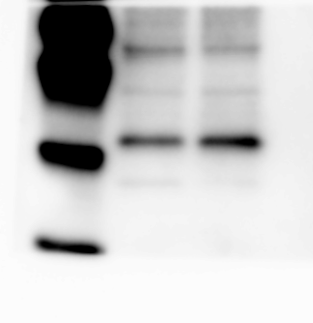

Supplement: Supplementary file 5 — Source data Fig. 3 [file 44318_2024_180_MOESM5_ESM.zip › 3A/pRab7-S72 lysates western.tif]

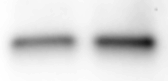

Supplement: Supplementary file 5 — Source data Fig. 3 [file 44318_2024_180_MOESM5_ESM.zip › 3A/pRab7-S72 lysosomes western cropped.tif]

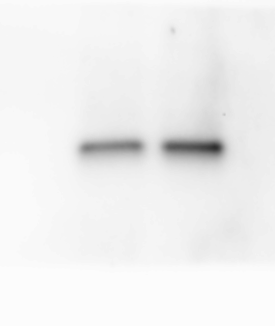

Supplement: Supplementary file 5 — Source data Fig. 3 [file 44318_2024_180_MOESM5_ESM.zip › 3A/pRab7-S72 lysosomes western.tif]

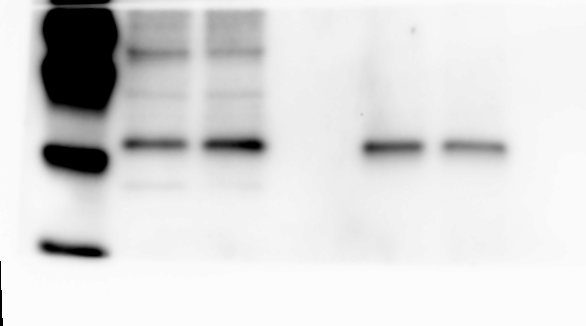

Supplement: Supplementary file 5 — Source data Fig. 3 [file 44318_2024_180_MOESM5_ESM.zip › 3A/pRab7-S72 western.tif]

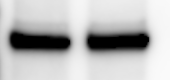

Supplement: Supplementary file 5 — Source data Fig. 3 [file 44318_2024_180_MOESM5_ESM.zip › 3A/pTBK1-S172 lysates western cropped.tif]

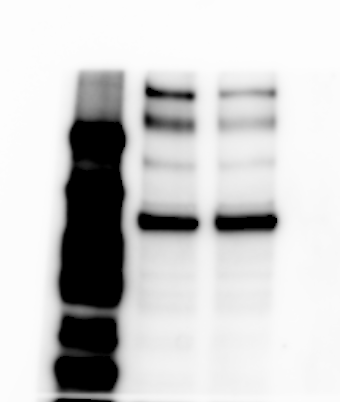

Supplement: Supplementary file 5 — Source data Fig. 3 [file 44318_2024_180_MOESM5_ESM.zip › 3A/pTBK1-S172 lysates western.tif]

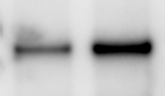

Supplement: Supplementary file 5 — Source data Fig. 3 [file 44318_2024_180_MOESM5_ESM.zip › 3A/pTBK1-S172 lysosomes western cropped.tif]

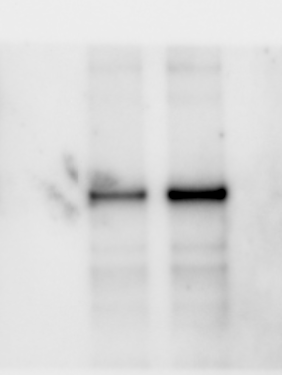

Supplement: Supplementary file 5 — Source data Fig. 3 [file 44318_2024_180_MOESM5_ESM.zip › 3A/pTBK1-S172 lysosomes western.tif]

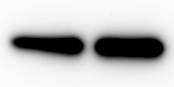

Supplement: Supplementary file 5 — Source data Fig. 3 [file 44318_2024_180_MOESM5_ESM.zip › 3A/Rab7 lysosomes western cropped.tif]

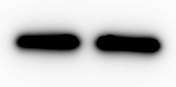

Supplement: Supplementary file 5 — Source data Fig. 3 [file 44318_2024_180_MOESM5_ESM.zip › 3A/Rab7 western lysates cropped.tif]

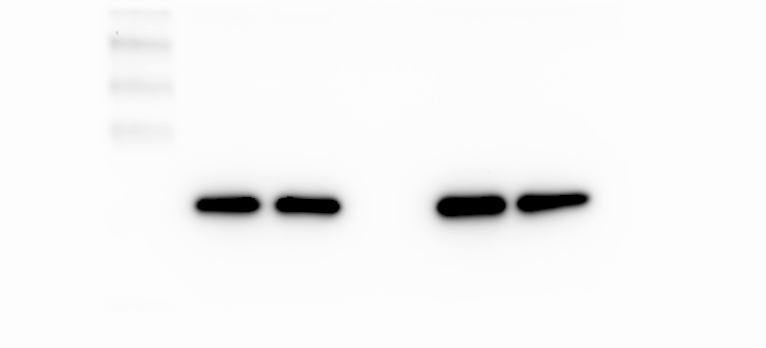

Supplement: Supplementary file 5 — Source data Fig. 3 [file 44318_2024_180_MOESM5_ESM.zip › 3A/Rab7 western.tif]

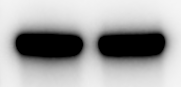

Supplement: Supplementary file 5 — Source data Fig. 3 [file 44318_2024_180_MOESM5_ESM.zip › 3A/TBK1 lysates western cropped.tif]

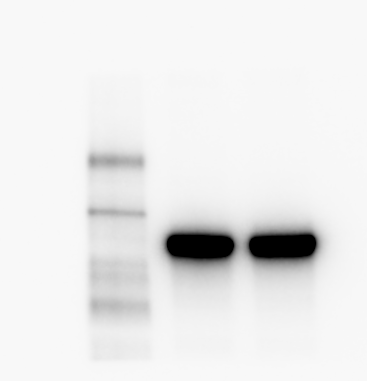

Supplement: Supplementary file 5 — Source data Fig. 3 [file 44318_2024_180_MOESM5_ESM.zip › 3A/TBK1 lysates western.tif]

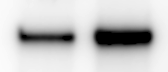

Supplement: Supplementary file 5 — Source data Fig. 3 [file 44318_2024_180_MOESM5_ESM.zip › 3A/TBK1 lysosomes western cropped.tif]

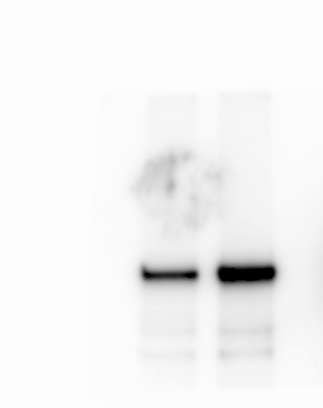

Supplement: Supplementary file 5 — Source data Fig. 3 [file 44318_2024_180_MOESM5_ESM.zip › 3A/TBK1 lysosomes western.tif]

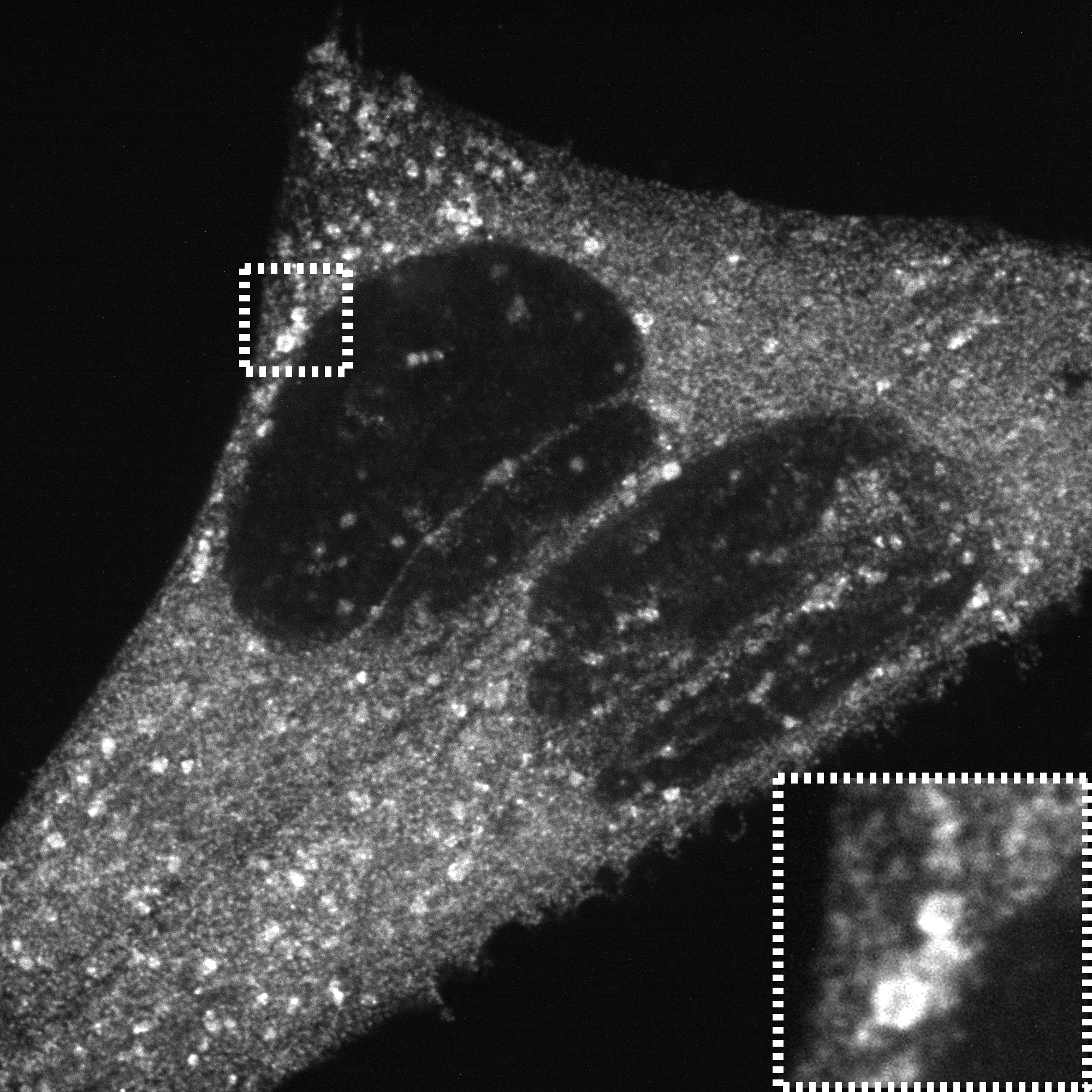

Supplement: Supplementary file 5 — Source data Fig. 3 [file 44318_2024_180_MOESM5_ESM.zip › 3F/TBK1-GFP + AAs GFP inset.tif]

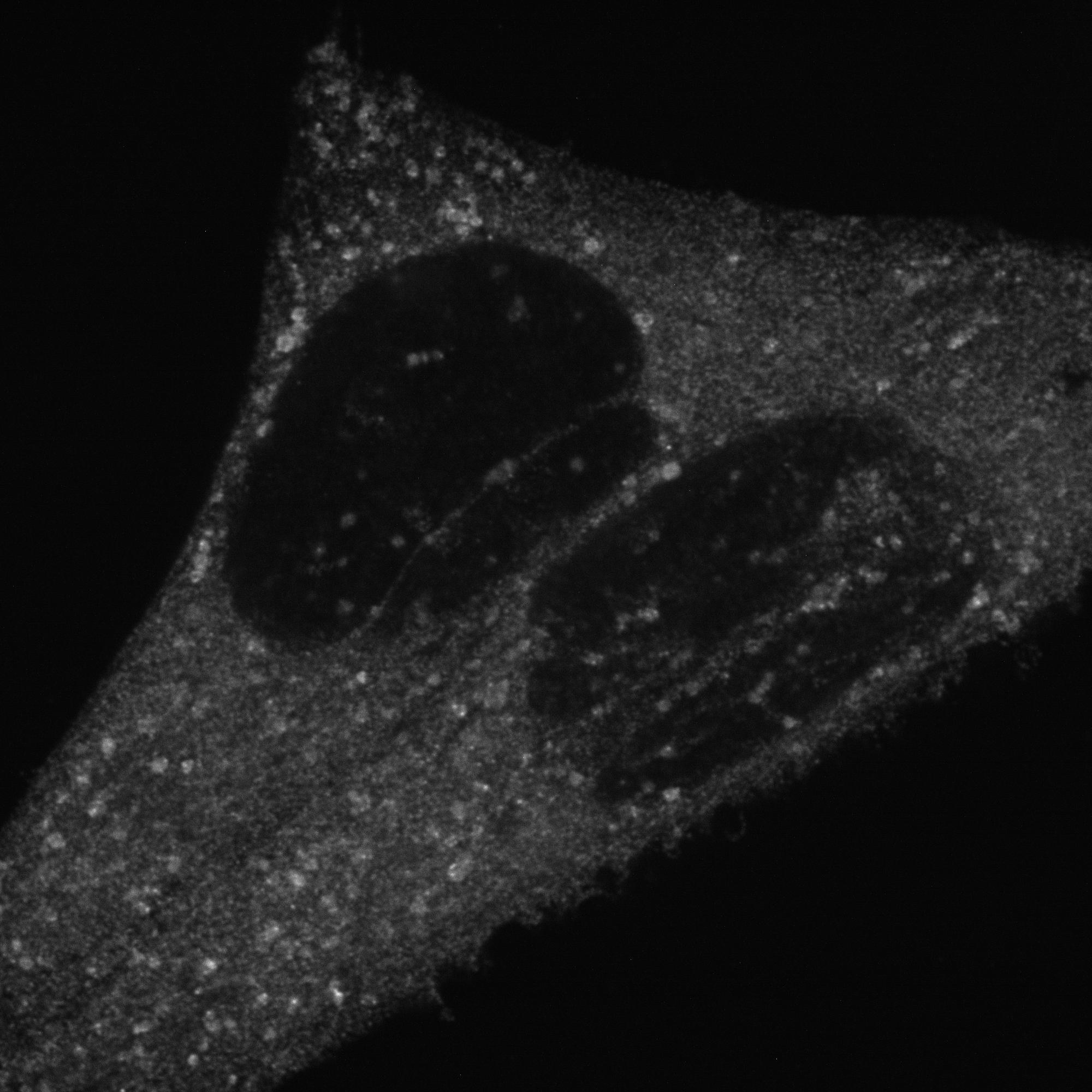

Supplement: Supplementary file 5 — Source data Fig. 3 [file 44318_2024_180_MOESM5_ESM.zip › 3F/TBK1-GFP + AAs GFP.tif]

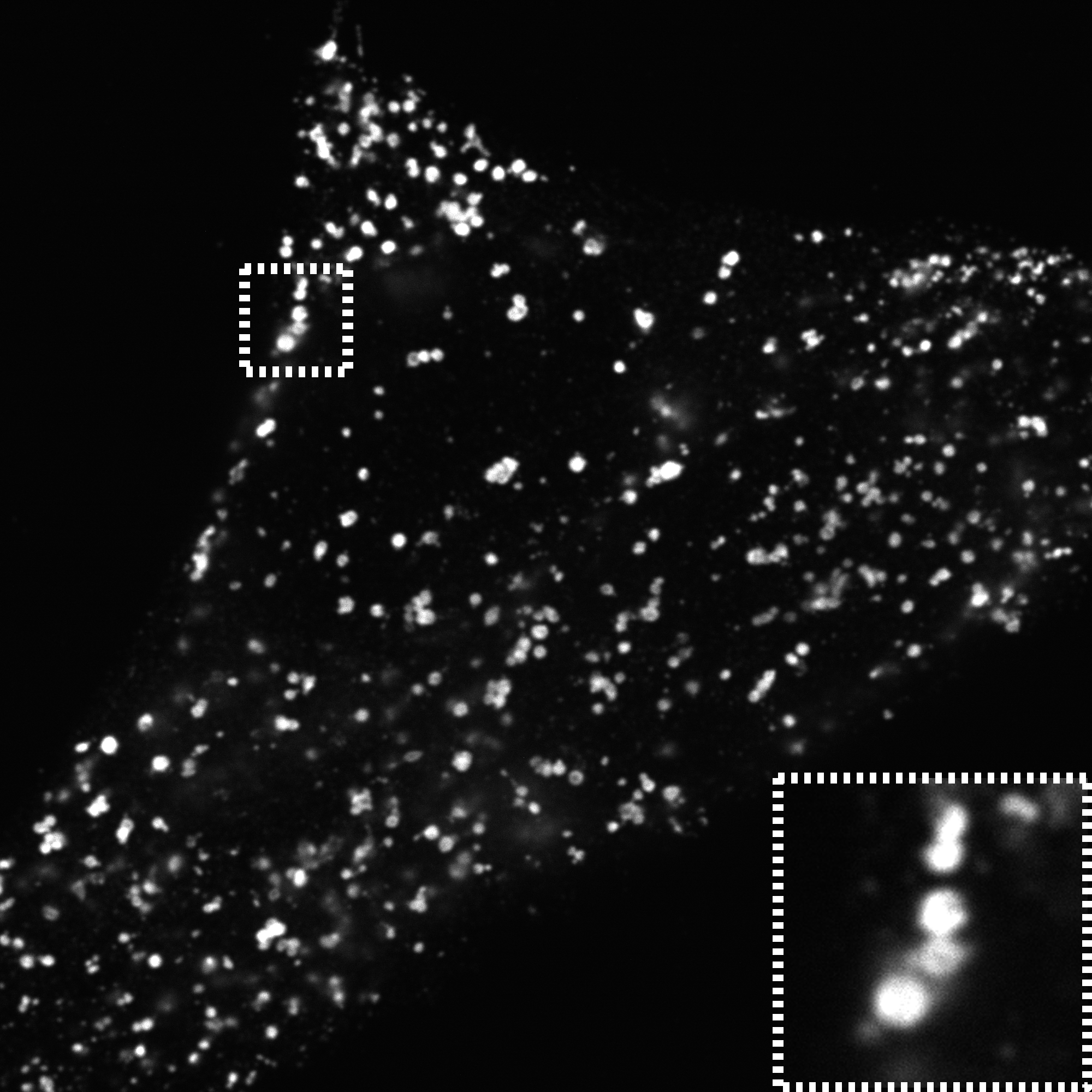

Supplement: Supplementary file 5 — Source data Fig. 3 [file 44318_2024_180_MOESM5_ESM.zip › 3F/TBK1-GFP + AAs LAMP1 inset.tif]

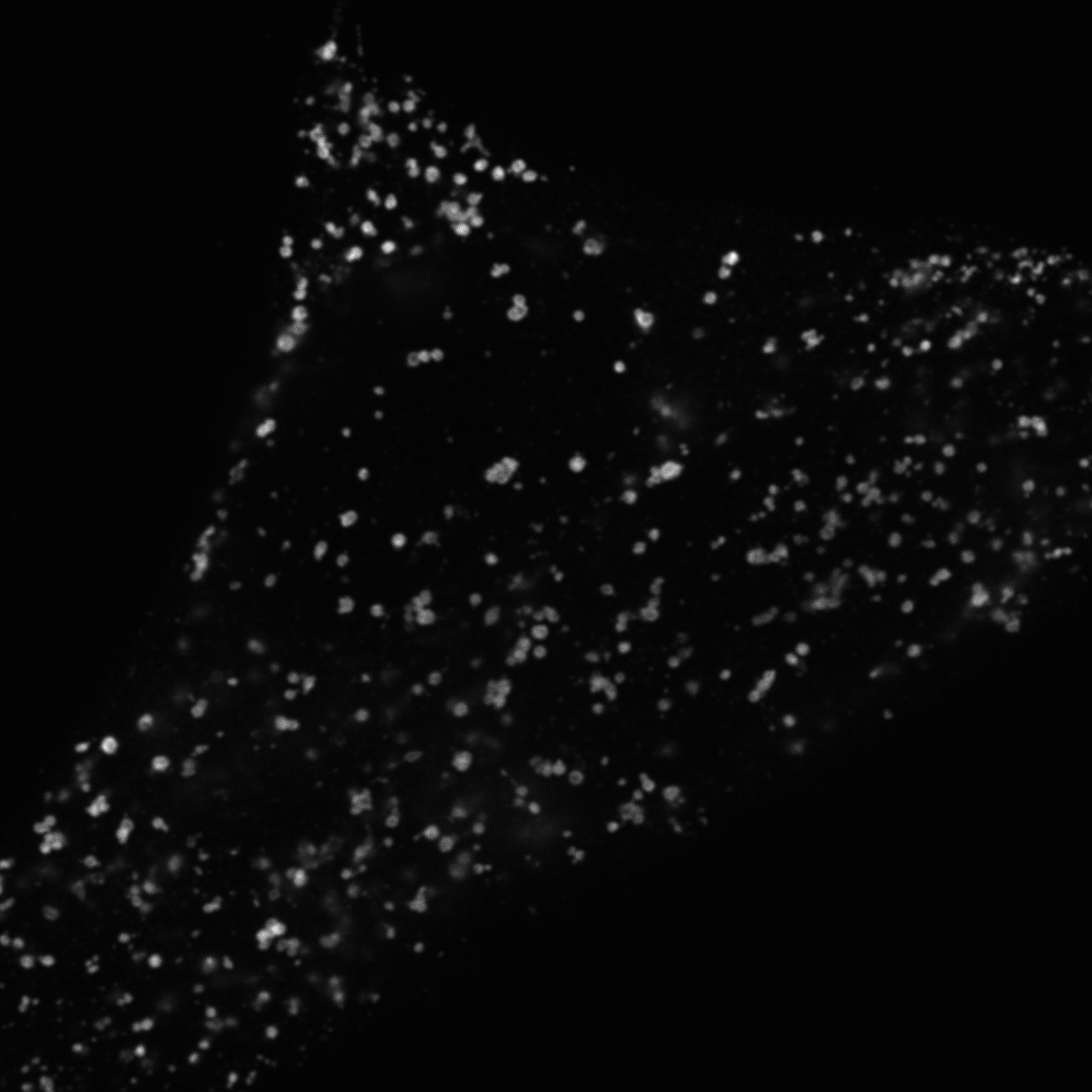

Supplement: Supplementary file 5 — Source data Fig. 3 [file 44318_2024_180_MOESM5_ESM.zip › 3F/TBK1-GFP + AAs LAMP1.tif]

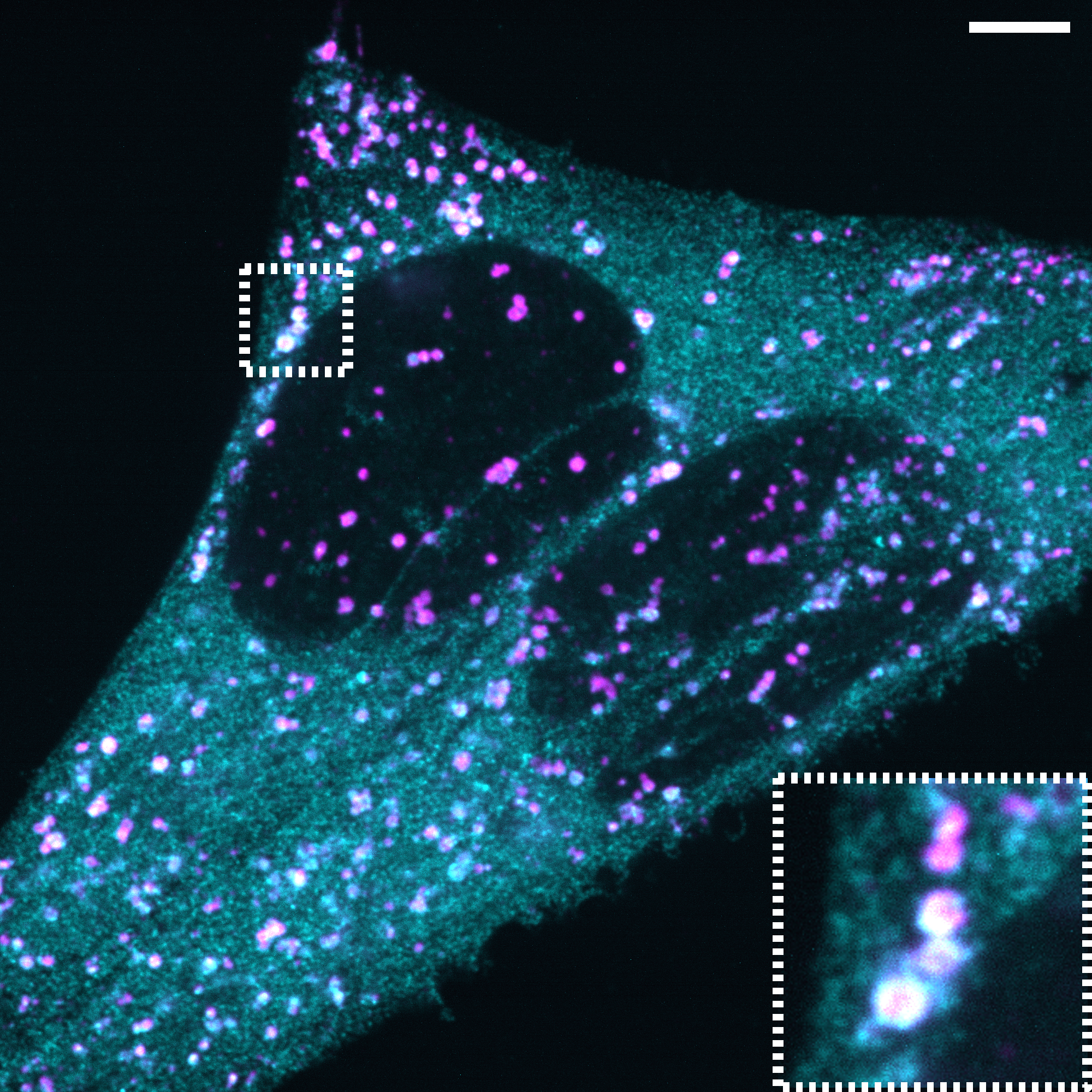

Supplement: Supplementary file 5 — Source data Fig. 3 [file 44318_2024_180_MOESM5_ESM.zip › 3F/TBK1-GFP + AAs Merge .tif]

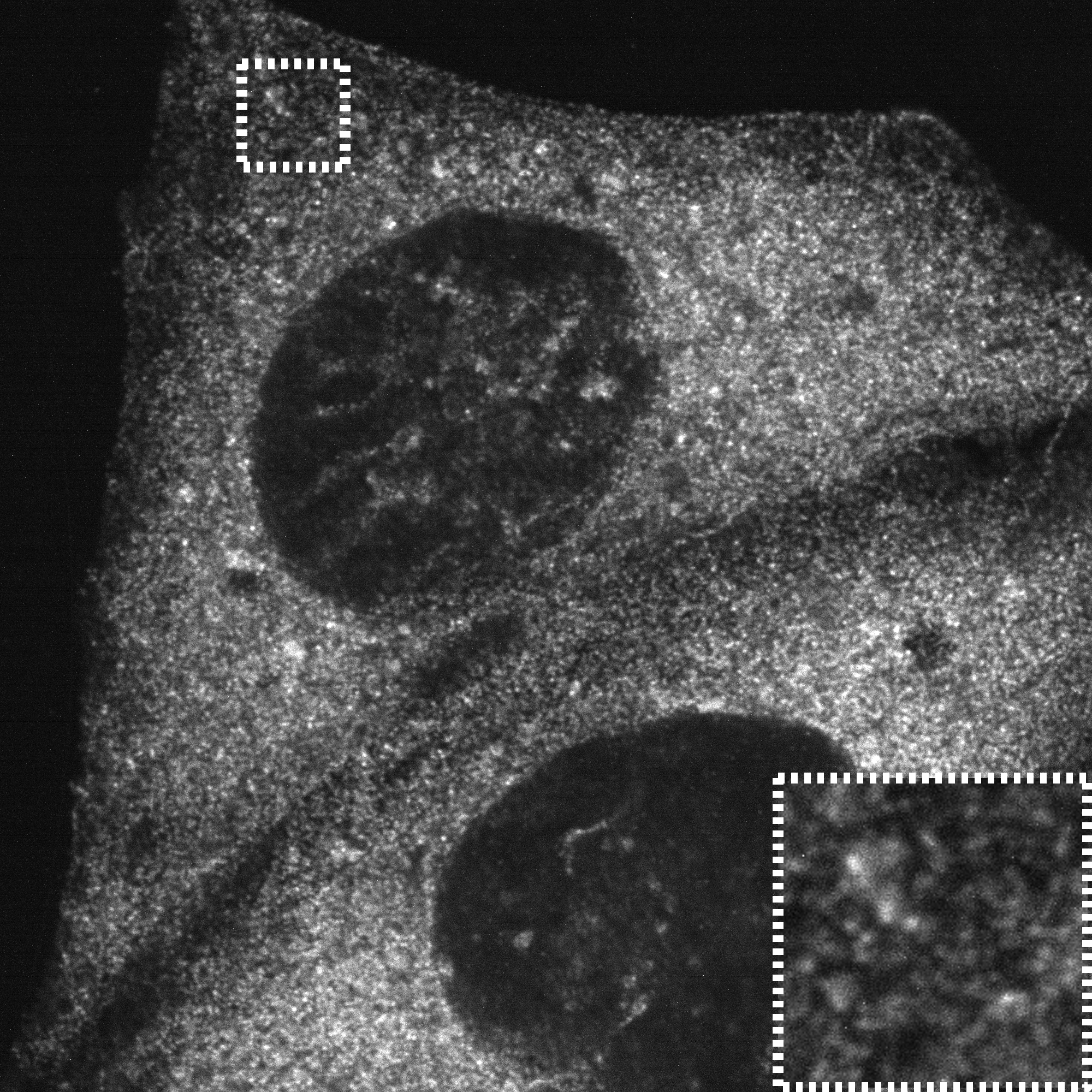

Supplement: Supplementary file 5 — Source data Fig. 3 [file 44318_2024_180_MOESM5_ESM.zip › 3F/TBK1-GFP - AAs GFP inset.tif]

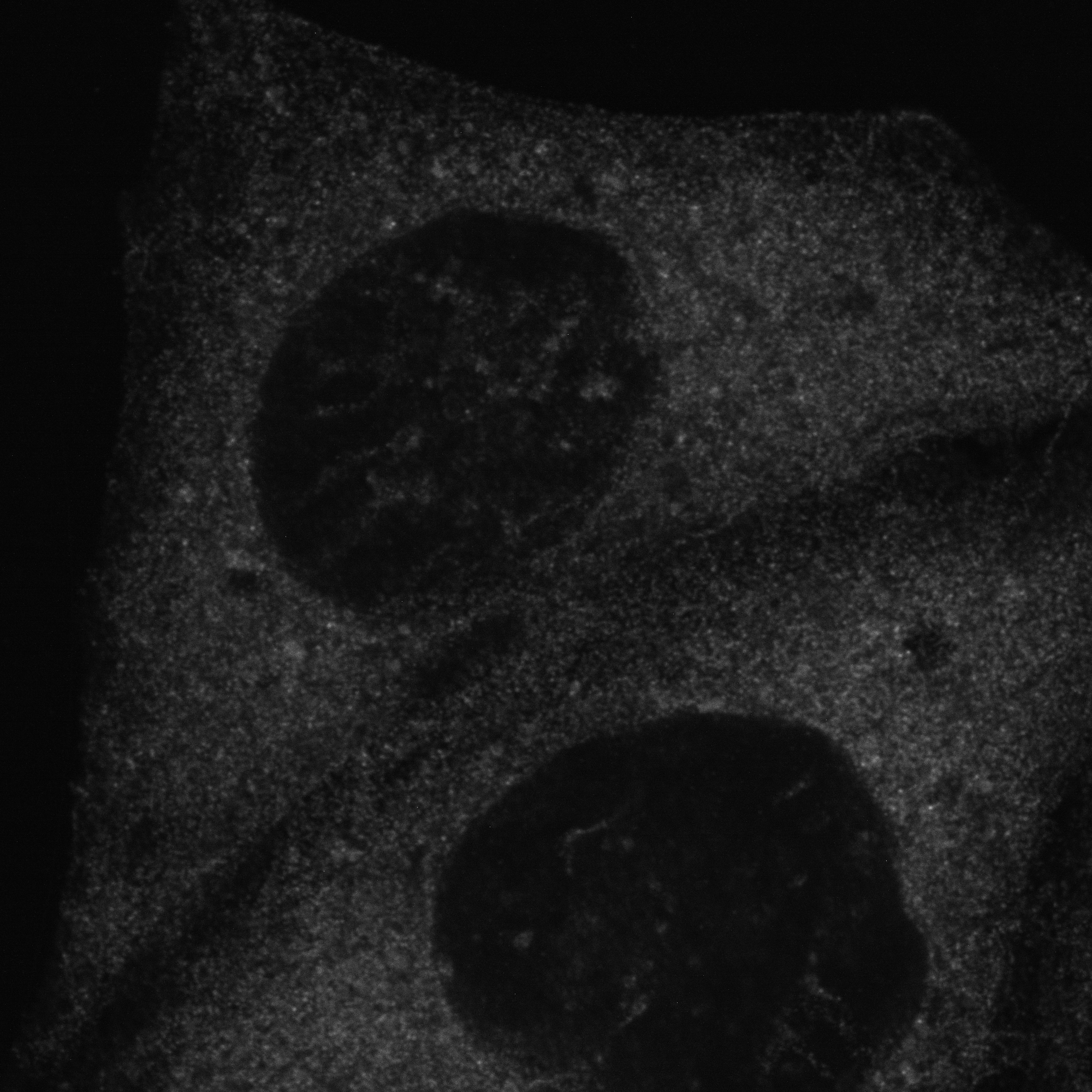

Supplement: Supplementary file 5 — Source data Fig. 3 [file 44318_2024_180_MOESM5_ESM.zip › 3F/TBK1-GFP - AAs GFP.tif]

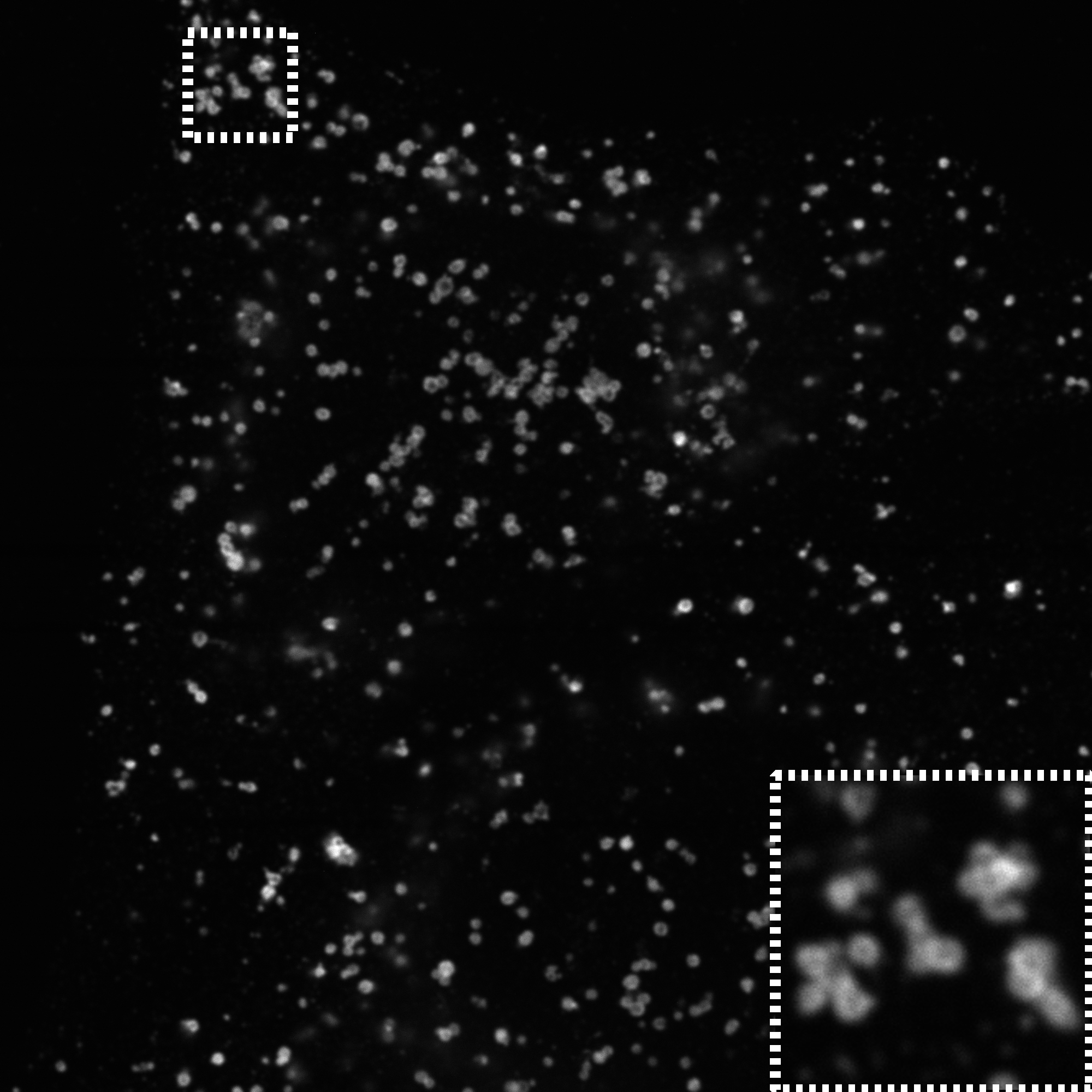

Supplement: Supplementary file 5 — Source data Fig. 3 [file 44318_2024_180_MOESM5_ESM.zip › 3F/TBK1-GFP - AAs LAMP1 inset.tif]

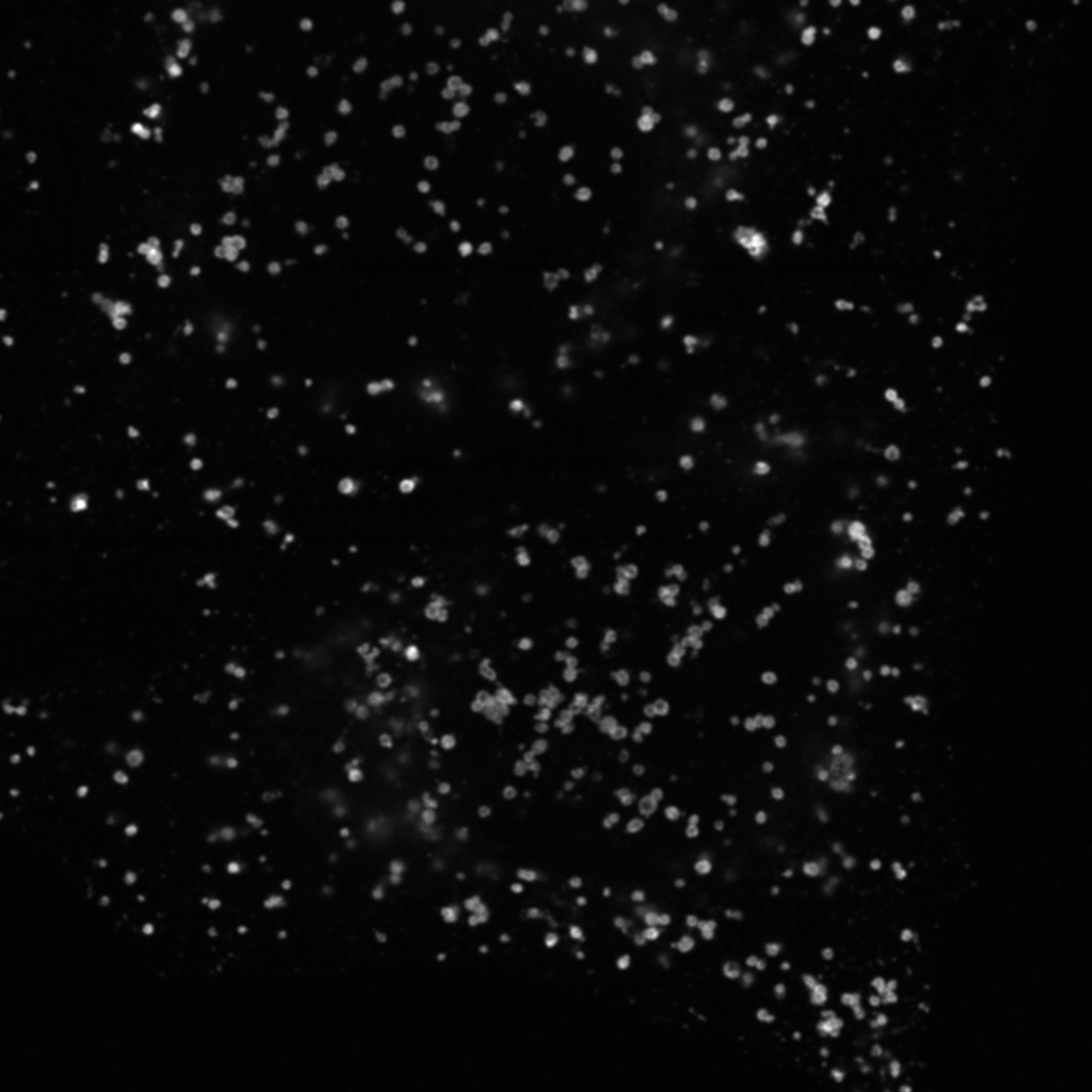

Supplement: Supplementary file 5 — Source data Fig. 3 [file 44318_2024_180_MOESM5_ESM.zip › 3F/TBK1-GFP - AAs LAMP1.tif]

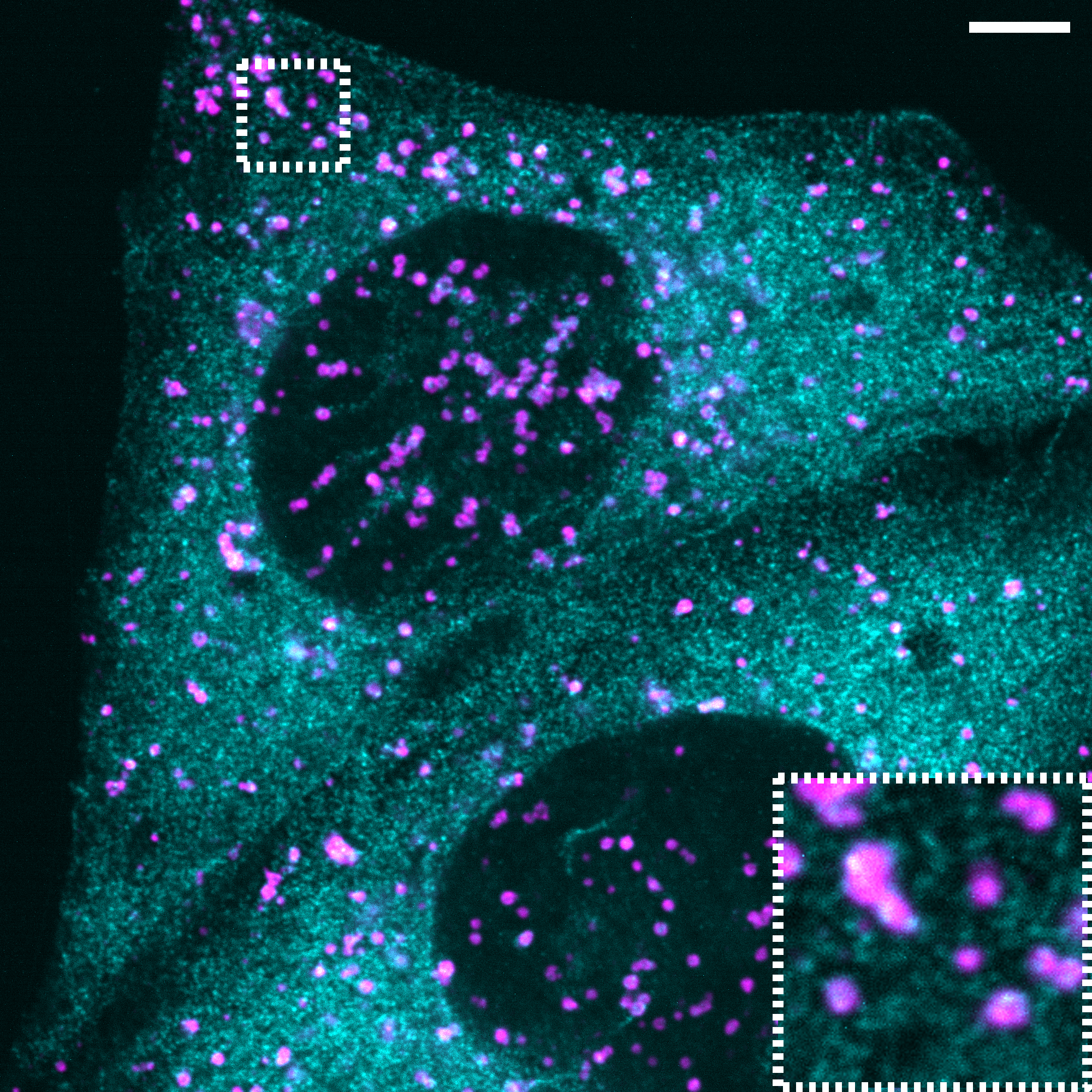

Supplement: Supplementary file 5 — Source data Fig. 3 [file 44318_2024_180_MOESM5_ESM.zip › 3F/TBK1-GFP - AAs Merge.tif]

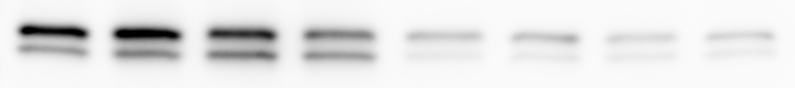

Supplement: Supplementary file 6 — Source data Fig. 4 [file 44318_2024_180_MOESM6_ESM.zip › 4A/pRab7-S72 western cropped.tif]

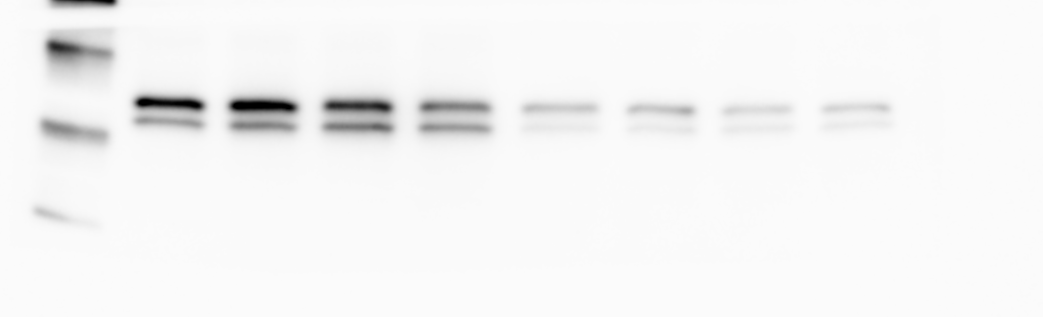

Supplement: Supplementary file 6 — Source data Fig. 4 [file 44318_2024_180_MOESM6_ESM.zip › 4A/pRab7-S72 western.tif]

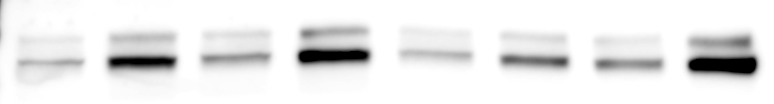

Supplement: Supplementary file 6 — Source data Fig. 4 [file 44318_2024_180_MOESM6_ESM.zip › 4A/pS6K1-T389 western cropped.tif]

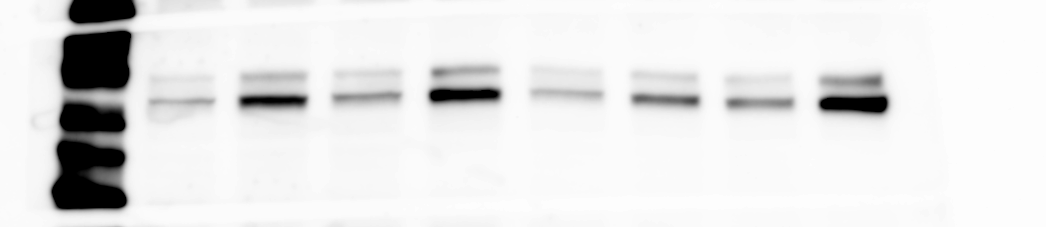

Supplement: Supplementary file 6 — Source data Fig. 4 [file 44318_2024_180_MOESM6_ESM.zip › 4A/pS6K1-T389 western.tif]

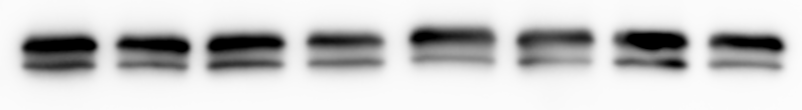

Supplement: Supplementary file 6 — Source data Fig. 4 [file 44318_2024_180_MOESM6_ESM.zip › 4A/Rab7 western cropped.tif]

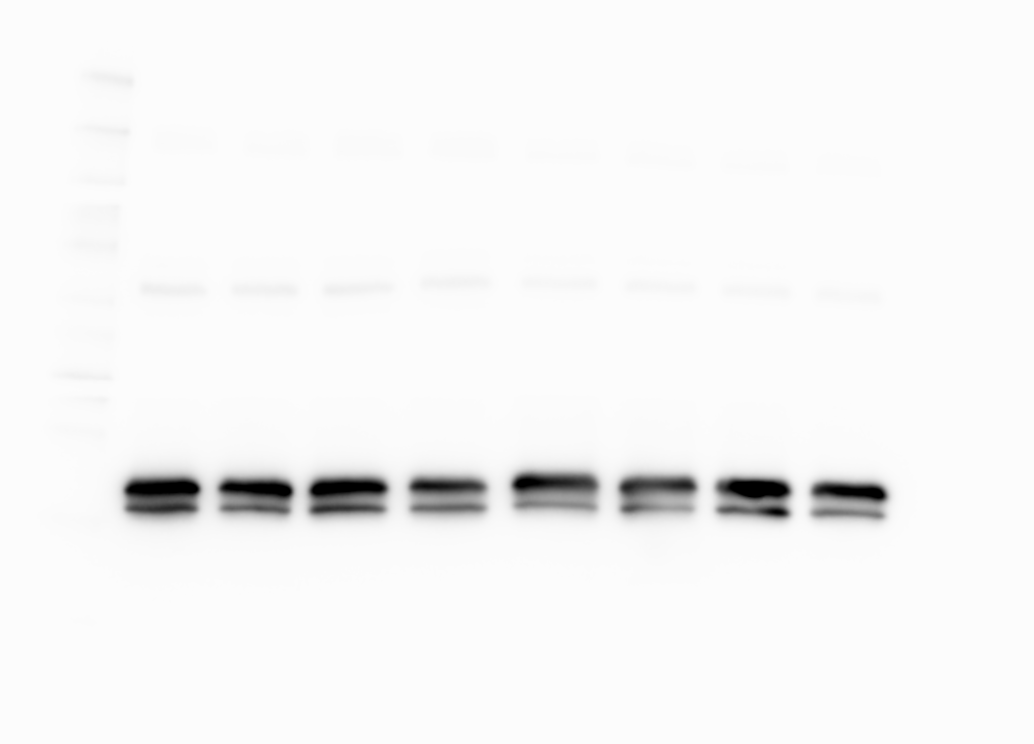

Supplement: Supplementary file 6 — Source data Fig. 4 [file 44318_2024_180_MOESM6_ESM.zip › 4A/Rab7 western.tif]

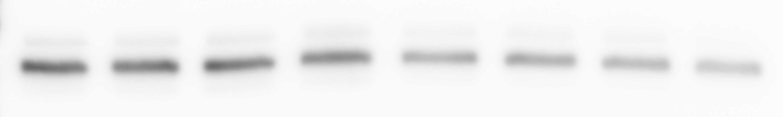

Supplement: Supplementary file 6 — Source data Fig. 4 [file 44318_2024_180_MOESM6_ESM.zip › 4A/S6K1 western cropped.tif]

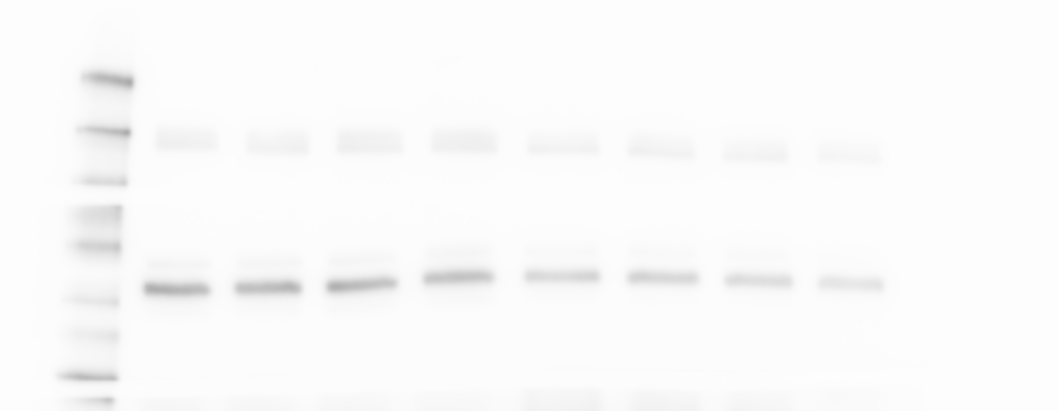

Supplement: Supplementary file 6 — Source data Fig. 4 [file 44318_2024_180_MOESM6_ESM.zip › 4A/S6K1 western.tif]

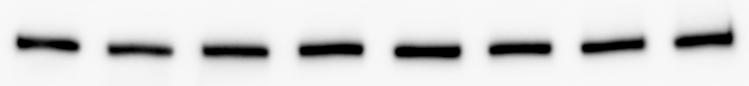

Supplement: Supplementary file 6 — Source data Fig. 4 [file 44318_2024_180_MOESM6_ESM.zip › 4B/mTOR western cropped.tif]

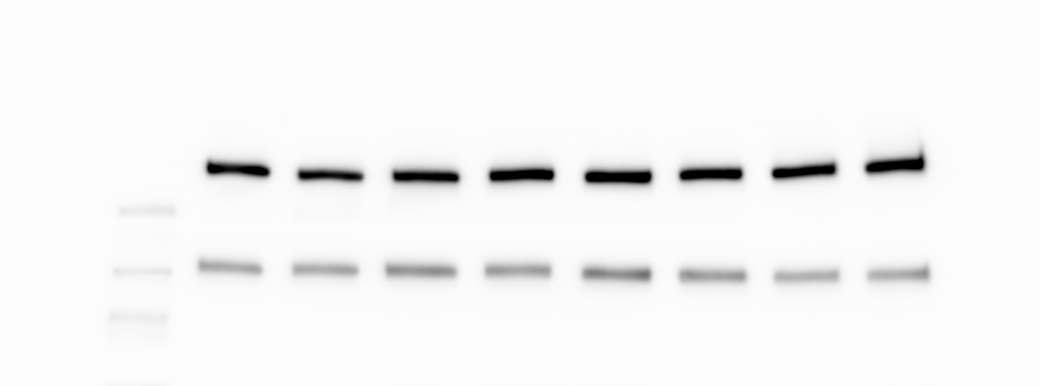

Supplement: Supplementary file 6 — Source data Fig. 4 [file 44318_2024_180_MOESM6_ESM.zip › 4B/mTOR western.tif]

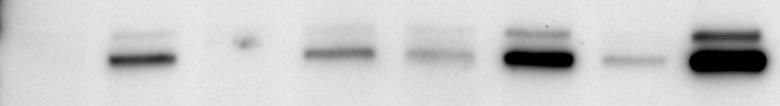

Supplement: Supplementary file 6 — Source data Fig. 4 [file 44318_2024_180_MOESM6_ESM.zip › 4B/pS6K1-T389 western cropped.tif]

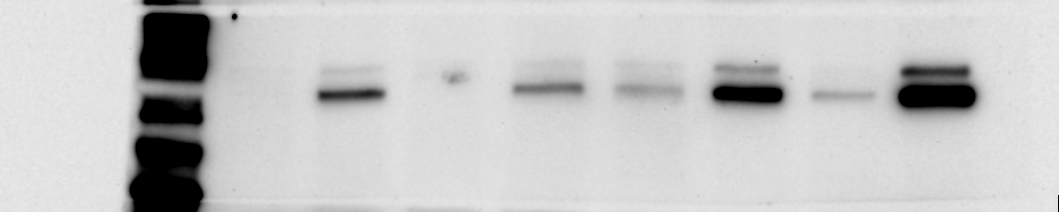

Supplement: Supplementary file 6 — Source data Fig. 4 [file 44318_2024_180_MOESM6_ESM.zip › 4B/pS6K1-T389 western.tif]

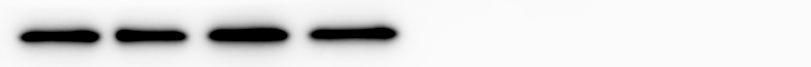

Supplement: Supplementary file 6 — Source data Fig. 4 [file 44318_2024_180_MOESM6_ESM.zip › 4B/Rab7 western cropped.tif]

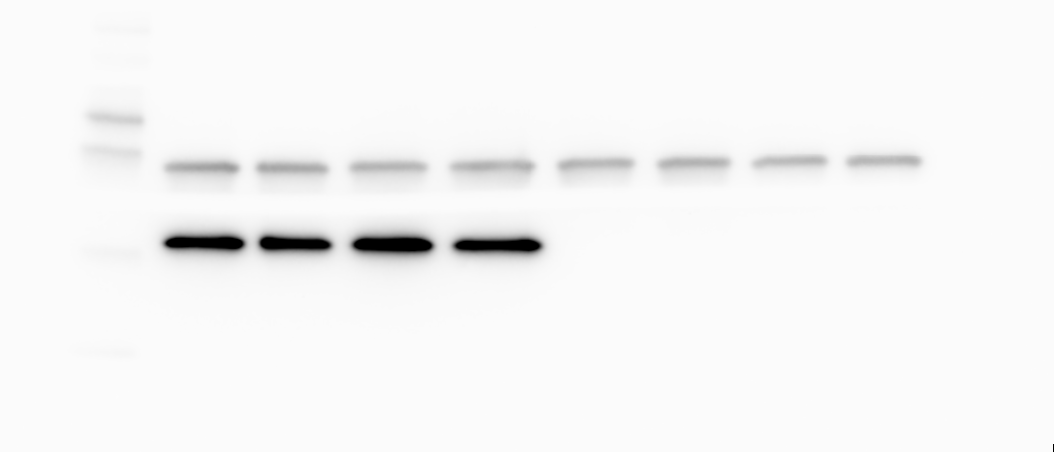

Supplement: Supplementary file 6 — Source data Fig. 4 [file 44318_2024_180_MOESM6_ESM.zip › 4B/Rab7 western.tif]

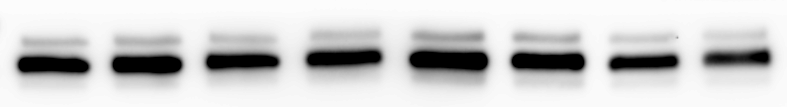

Supplement: Supplementary file 6 — Source data Fig. 4 [file 44318_2024_180_MOESM6_ESM.zip › 4B/S6K1 western cropped.tif]

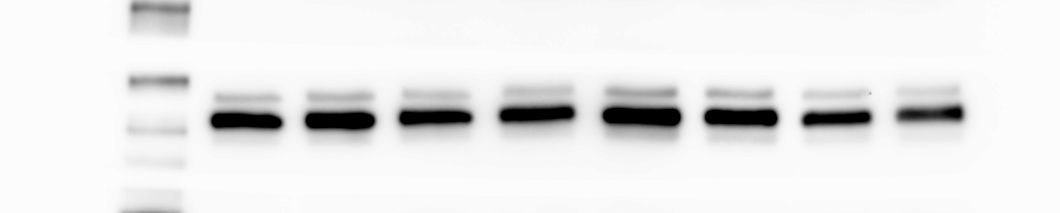

Supplement: Supplementary file 6 — Source data Fig. 4 [file 44318_2024_180_MOESM6_ESM.zip › 4B/S6K1 western.tif]

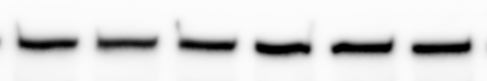

Supplement: Supplementary file 6 — Source data Fig. 4 [file 44318_2024_180_MOESM6_ESM.zip › 4D/mTOR western cropped.tif]

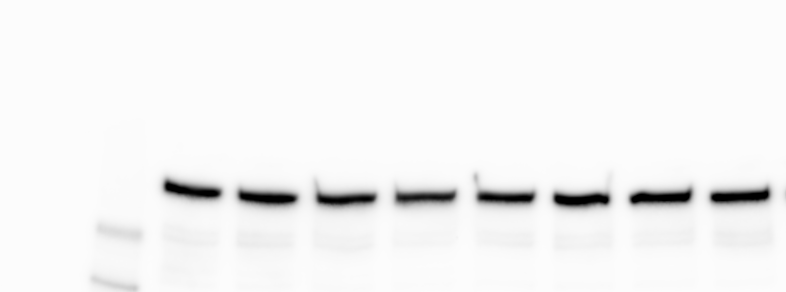

Supplement: Supplementary file 6 — Source data Fig. 4 [file 44318_2024_180_MOESM6_ESM.zip › 4D/mTOR western.tif]

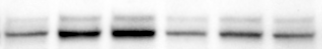

Supplement: Supplementary file 6 — Source data Fig. 4 [file 44318_2024_180_MOESM6_ESM.zip › 4D/pS6K1-T389 western cropped.tif]

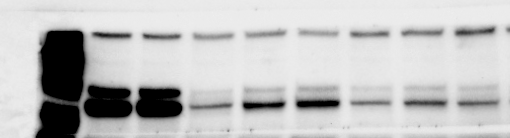

Supplement: Supplementary file 6 — Source data Fig. 4 [file 44318_2024_180_MOESM6_ESM.zip › 4D/pS6K1-T389 western.tif]

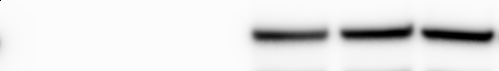

Supplement: Supplementary file 6 — Source data Fig. 4 [file 44318_2024_180_MOESM6_ESM.zip › 4D/Rab7 western cropped.tif]

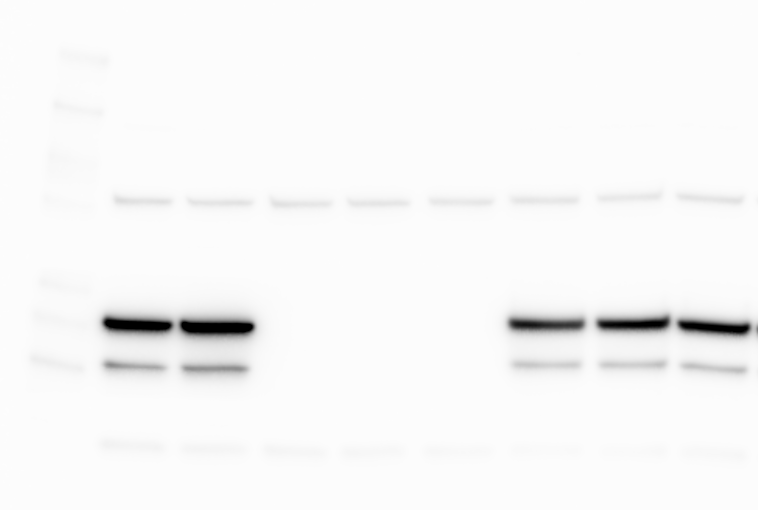

Supplement: Supplementary file 6 — Source data Fig. 4 [file 44318_2024_180_MOESM6_ESM.zip › 4D/Rab7 western.tif]

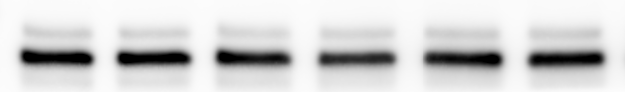

Supplement: Supplementary file 6 — Source data Fig. 4 [file 44318_2024_180_MOESM6_ESM.zip › 4D/S6K1 western cropped.tif]

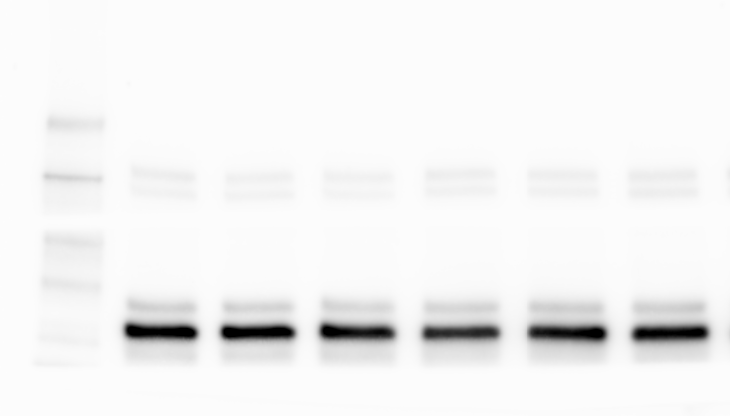

Supplement: Supplementary file 6 — Source data Fig. 4 [file 44318_2024_180_MOESM6_ESM.zip › 4D/S6K1 western.tif]

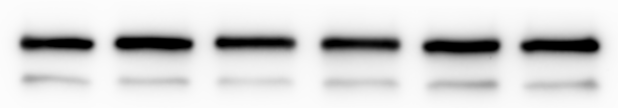

Supplement: Supplementary file 7 — Source data Fig. 5 [file 44318_2024_180_MOESM7_ESM.zip › 5A/mCh-Rab7 western cropped.tif]

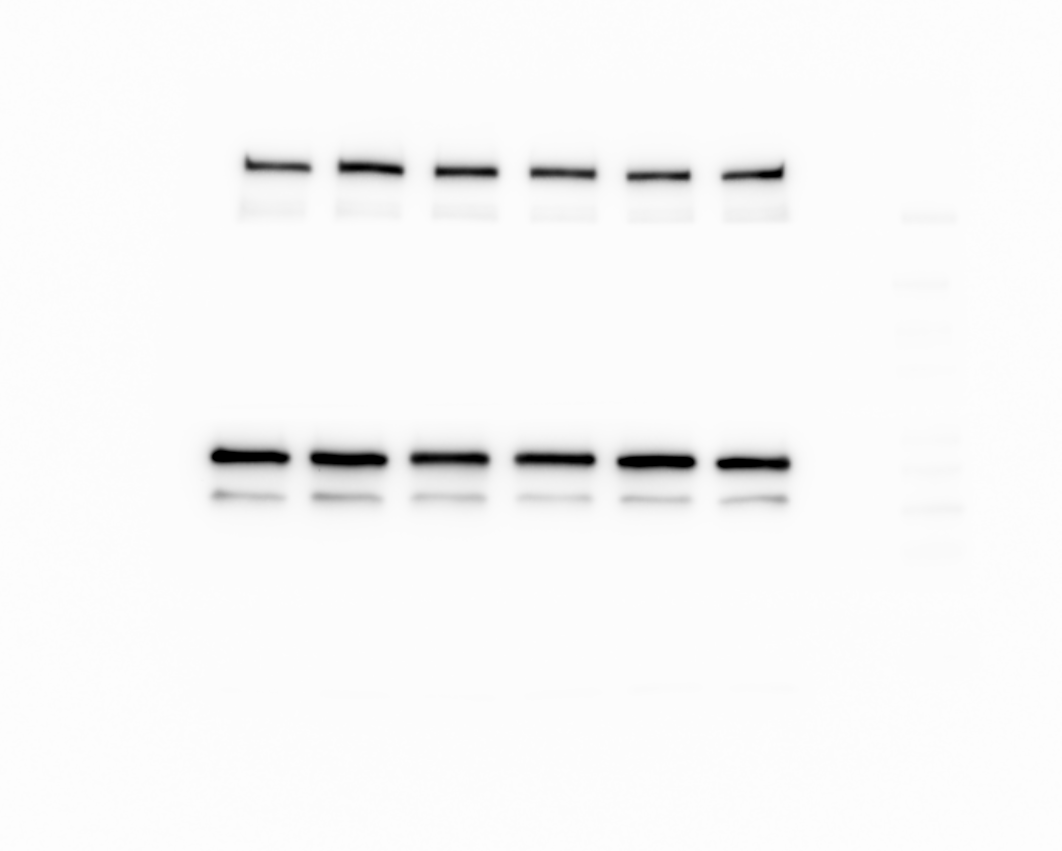

Supplement: Supplementary file 7 — Source data Fig. 5 [file 44318_2024_180_MOESM7_ESM.zip › 5A/mTOR and Rab7 western.tif]

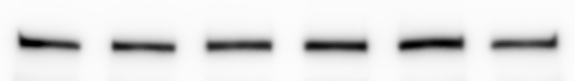

Supplement: Supplementary file 7 — Source data Fig. 5 [file 44318_2024_180_MOESM7_ESM.zip › 5A/mTOR western cropped.tif]

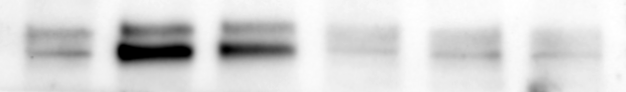

Supplement: Supplementary file 7 — Source data Fig. 5 [file 44318_2024_180_MOESM7_ESM.zip › 5A/pS6K1-T389 western cropped.tif]

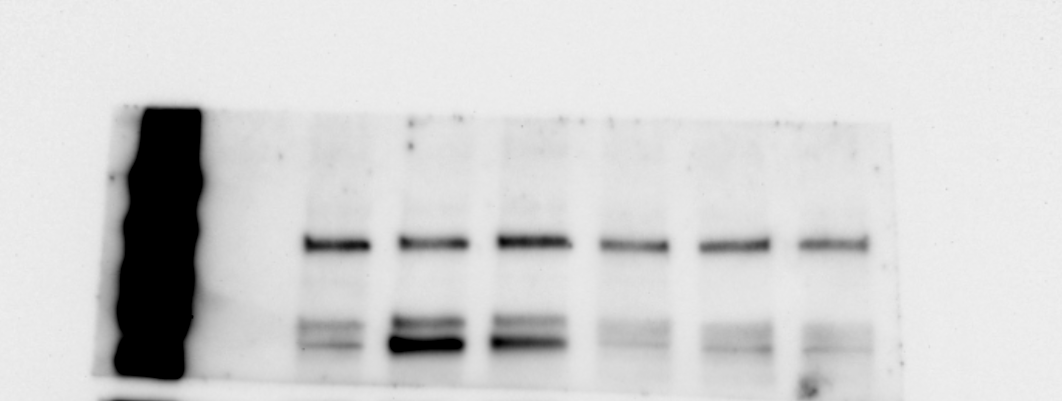

Supplement: Supplementary file 7 — Source data Fig. 5 [file 44318_2024_180_MOESM7_ESM.zip › 5A/pS6K1-T389 western.tif]

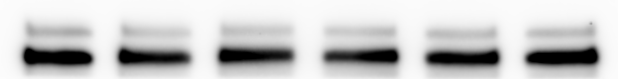

Supplement: Supplementary file 7 — Source data Fig. 5 [file 44318_2024_180_MOESM7_ESM.zip › 5A/S6K1 western cropped.tif]

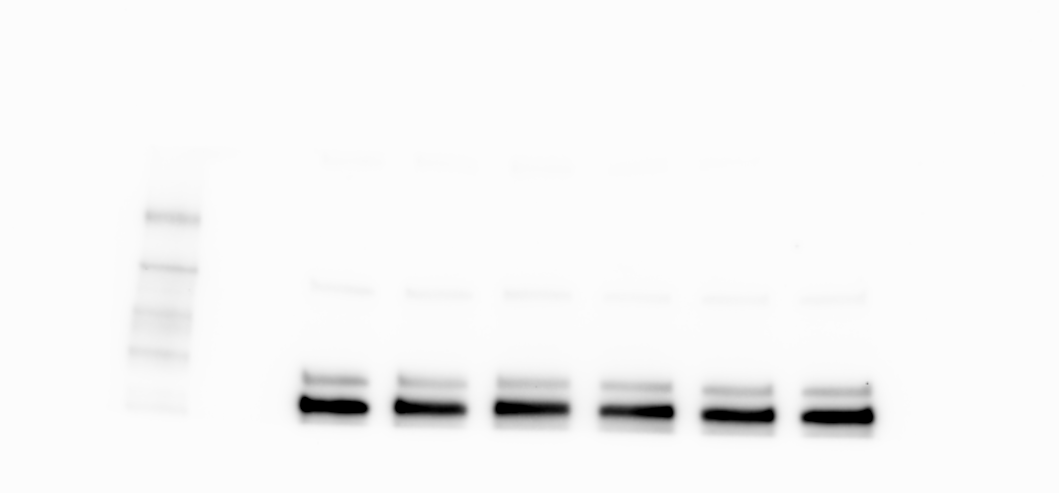

Supplement: Supplementary file 7 — Source data Fig. 5 [file 44318_2024_180_MOESM7_ESM.zip › 5A/S6K1 western.tif]

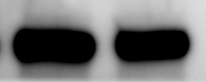

Supplement: Supplementary file 7 — Source data Fig. 5 [file 44318_2024_180_MOESM7_ESM.zip › 5C/GM130 lysates western cropped.tif]

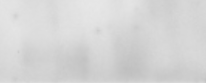

Supplement: Supplementary file 7 — Source data Fig. 5 [file 44318_2024_180_MOESM7_ESM.zip › 5C/GM130 lysosomes western cropped.tif]

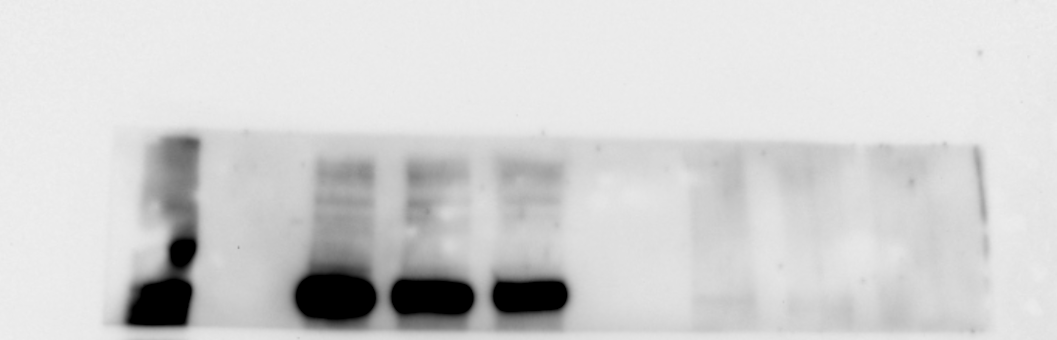

Supplement: Supplementary file 7 — Source data Fig. 5 [file 44318_2024_180_MOESM7_ESM.zip › 5C/GM130 western.tif]

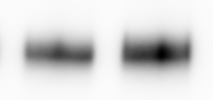

Supplement: Supplementary file 7 — Source data Fig. 5 [file 44318_2024_180_MOESM7_ESM.zip › 5C/LAMP1 lysates western cropped.tif]

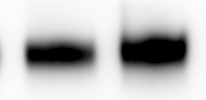

Supplement: Supplementary file 7 — Source data Fig. 5 [file 44318_2024_180_MOESM7_ESM.zip › 5C/LAMP1 lysosomes western cropped.tif]

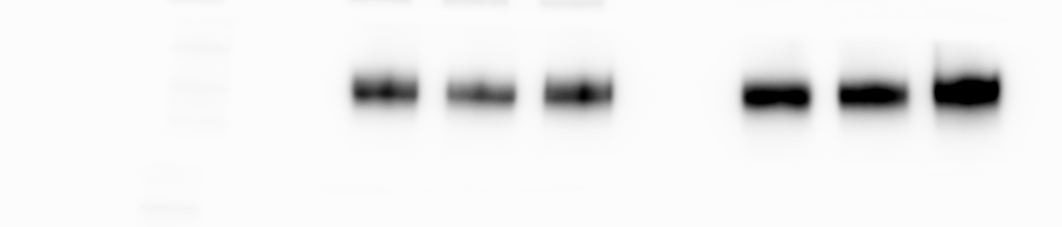

Supplement: Supplementary file 7 — Source data Fig. 5 [file 44318_2024_180_MOESM7_ESM.zip › 5C/LAMP1 western.tif]

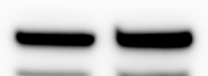

Supplement: Supplementary file 7 — Source data Fig. 5 [file 44318_2024_180_MOESM7_ESM.zip › 5C/mCh-Rab7 lysates western cropped.tif]

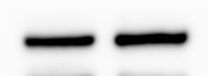

Supplement: Supplementary file 7 — Source data Fig. 5 [file 44318_2024_180_MOESM7_ESM.zip › 5C/mCh-Rab7 lysosomes western cropped.tif]

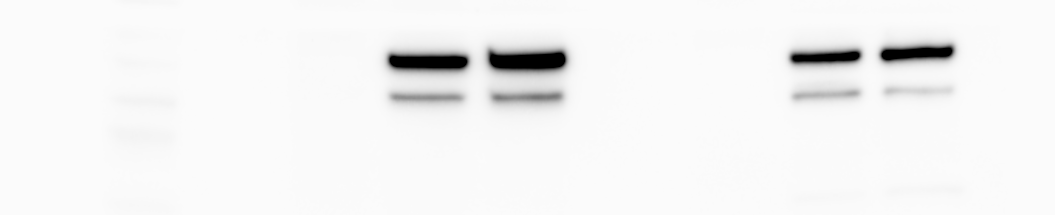

Supplement: Supplementary file 7 — Source data Fig. 5 [file 44318_2024_180_MOESM7_ESM.zip › 5C/mCh-Rab7 western.tif]

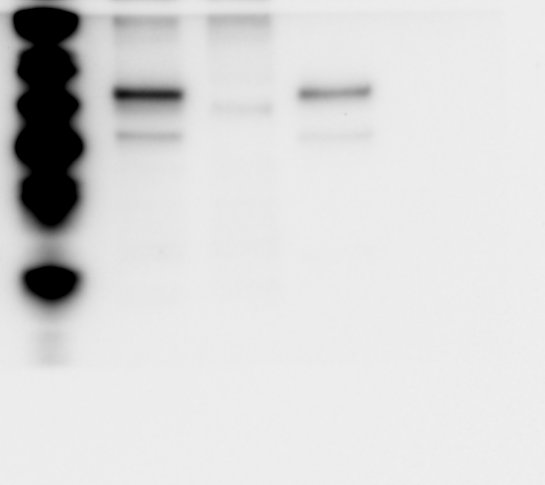

Supplement: Supplementary file 7 — Source data Fig. 5 [file 44318_2024_180_MOESM7_ESM.zip › 5C/p-mCh-Rab7 western.tif]

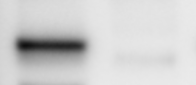

Supplement: Supplementary file 7 — Source data Fig. 5 [file 44318_2024_180_MOESM7_ESM.zip › 5C/p-mCh-Rab7-S72 lysates western cropped.tif]

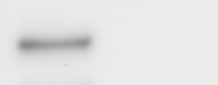

Supplement: Supplementary file 7 — Source data Fig. 5 [file 44318_2024_180_MOESM7_ESM.zip › 5C/p-mCh-Rab7-S72 lysosomes western cropped.tif]

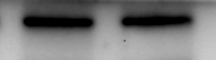

Supplement: Supplementary file 7 — Source data Fig. 5 [file 44318_2024_180_MOESM7_ESM.zip › 5C/PDI western lysates cropped.tif]

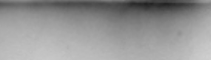

Supplement: Supplementary file 7 — Source data Fig. 5 [file 44318_2024_180_MOESM7_ESM.zip › 5C/PDI western lysosomes cropped.tif]

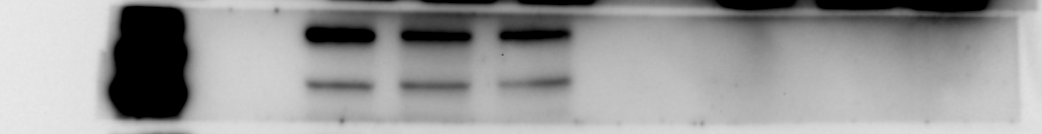

Supplement: Supplementary file 7 — Source data Fig. 5 [file 44318_2024_180_MOESM7_ESM.zip › 5C/PDI western raw.tif]

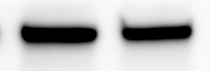

Supplement: Supplementary file 7 — Source data Fig. 5 [file 44318_2024_180_MOESM7_ESM.zip › 5C/TBK1 lysates western cropped.tif]

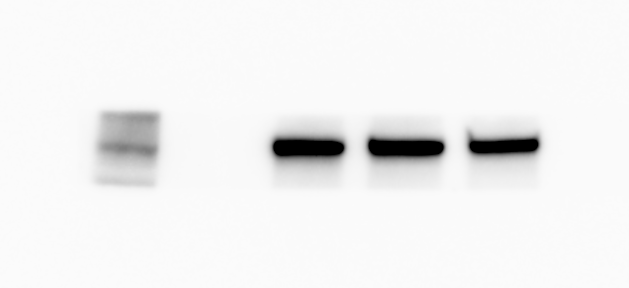

Supplement: Supplementary file 7 — Source data Fig. 5 [file 44318_2024_180_MOESM7_ESM.zip › 5C/TBK1 lysates western.tif]

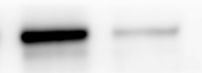

Supplement: Supplementary file 7 — Source data Fig. 5 [file 44318_2024_180_MOESM7_ESM.zip › 5C/TBK1 lysosomes western cropped.tif]

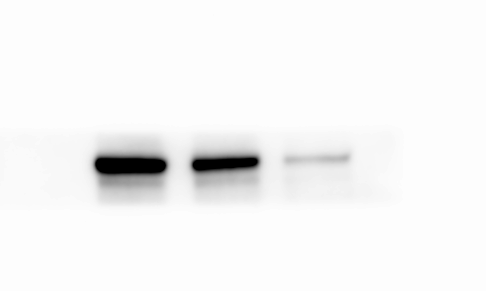

Supplement: Supplementary file 7 — Source data Fig. 5 [file 44318_2024_180_MOESM7_ESM.zip › 5C/TBK1 lysosomes western.tif]

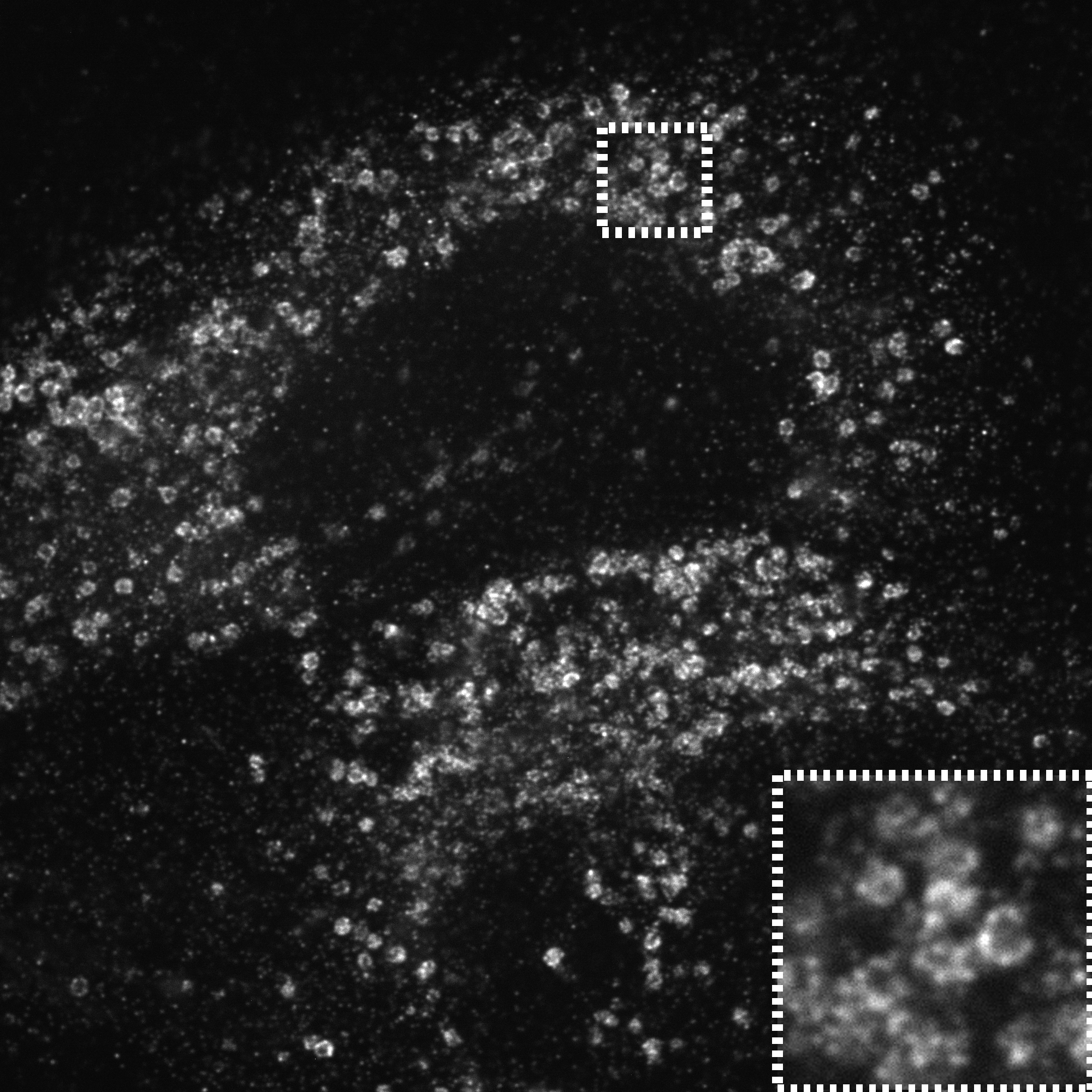

Supplement: Supplementary file 8 — Source data Fig. 6 [file 44318_2024_180_MOESM8_ESM.zip › 6A/TBK1-E696K GFP inset.tif]

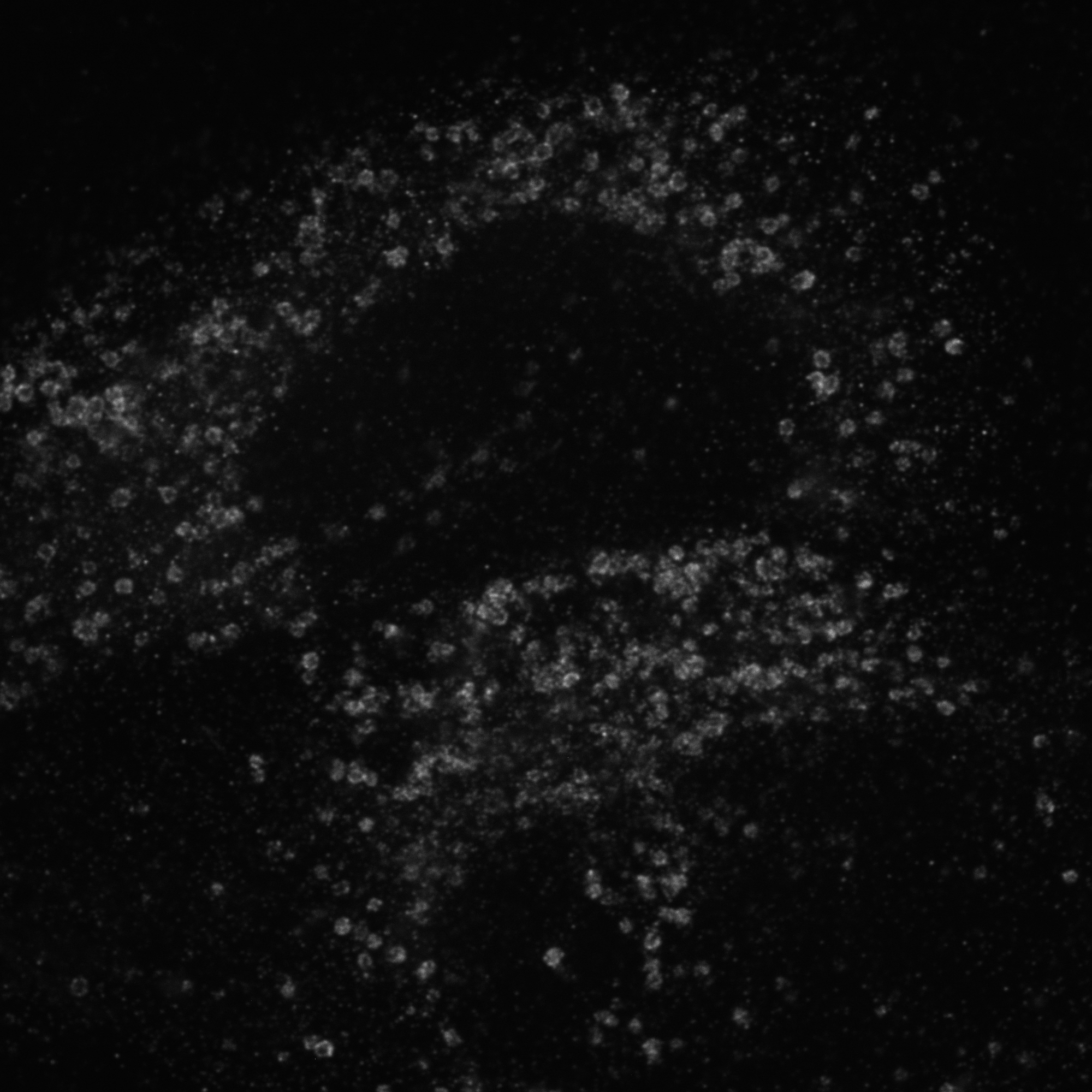

Supplement: Supplementary file 8 — Source data Fig. 6 [file 44318_2024_180_MOESM8_ESM.zip › 6A/TBK1-E696K GFP.tif]

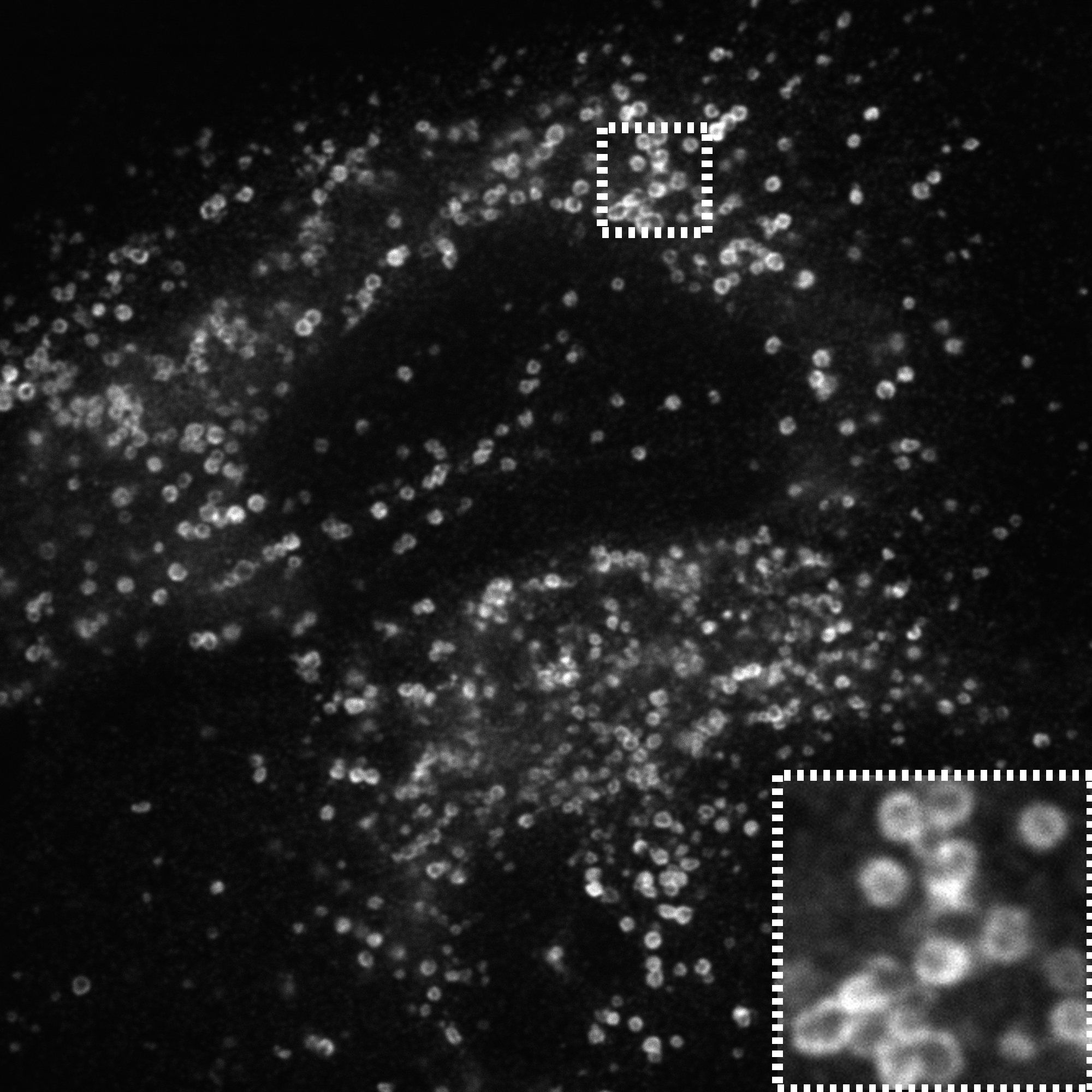

Supplement: Supplementary file 8 — Source data Fig. 6 [file 44318_2024_180_MOESM8_ESM.zip › 6A/TBK1-E696K LAMP1 inset.tif]

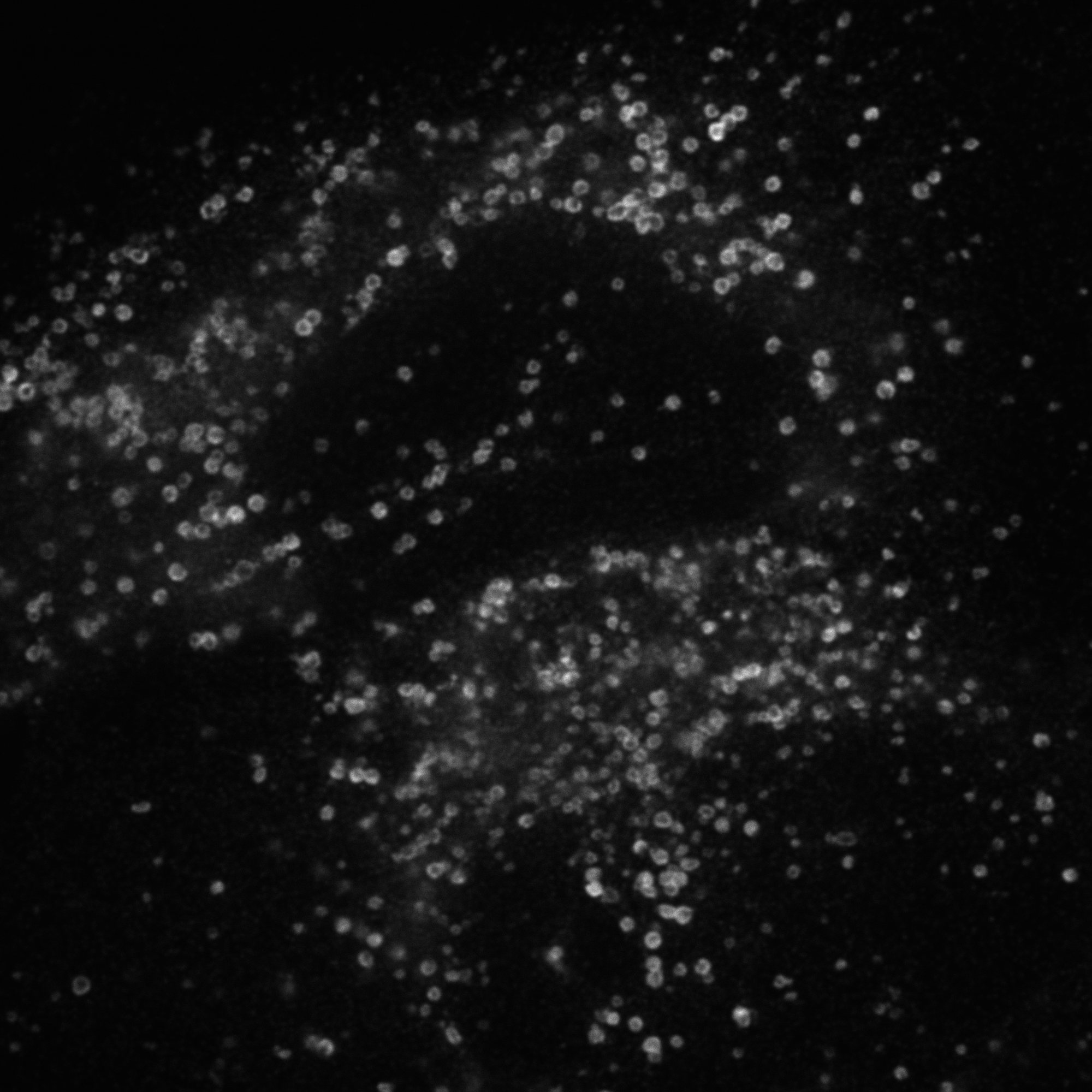

Supplement: Supplementary file 8 — Source data Fig. 6 [file 44318_2024_180_MOESM8_ESM.zip › 6A/TBK1-E696K LAMP1.tif]

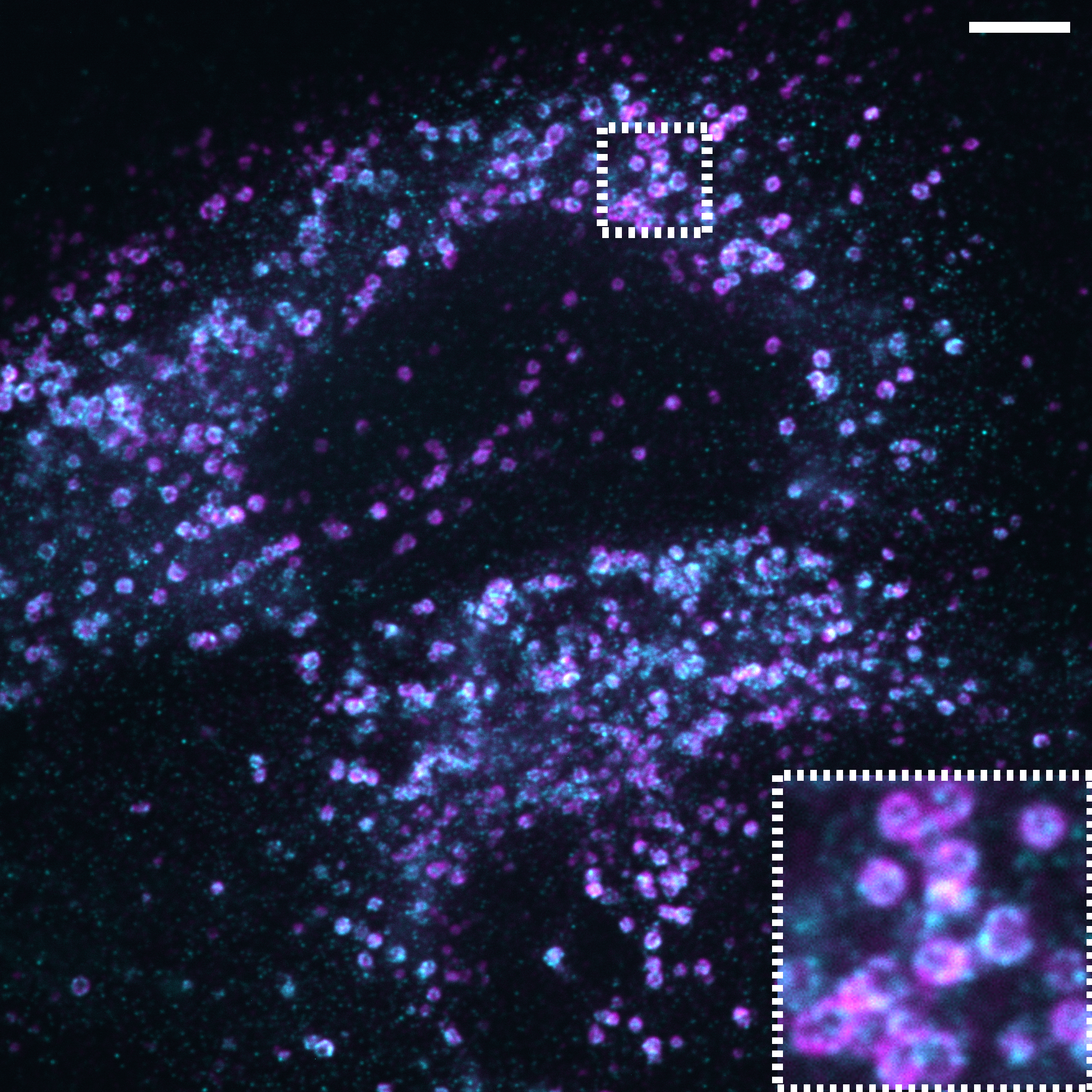

Supplement: Supplementary file 8 — Source data Fig. 6 [file 44318_2024_180_MOESM8_ESM.zip › 6A/TBK1-E696K Merge.tif]

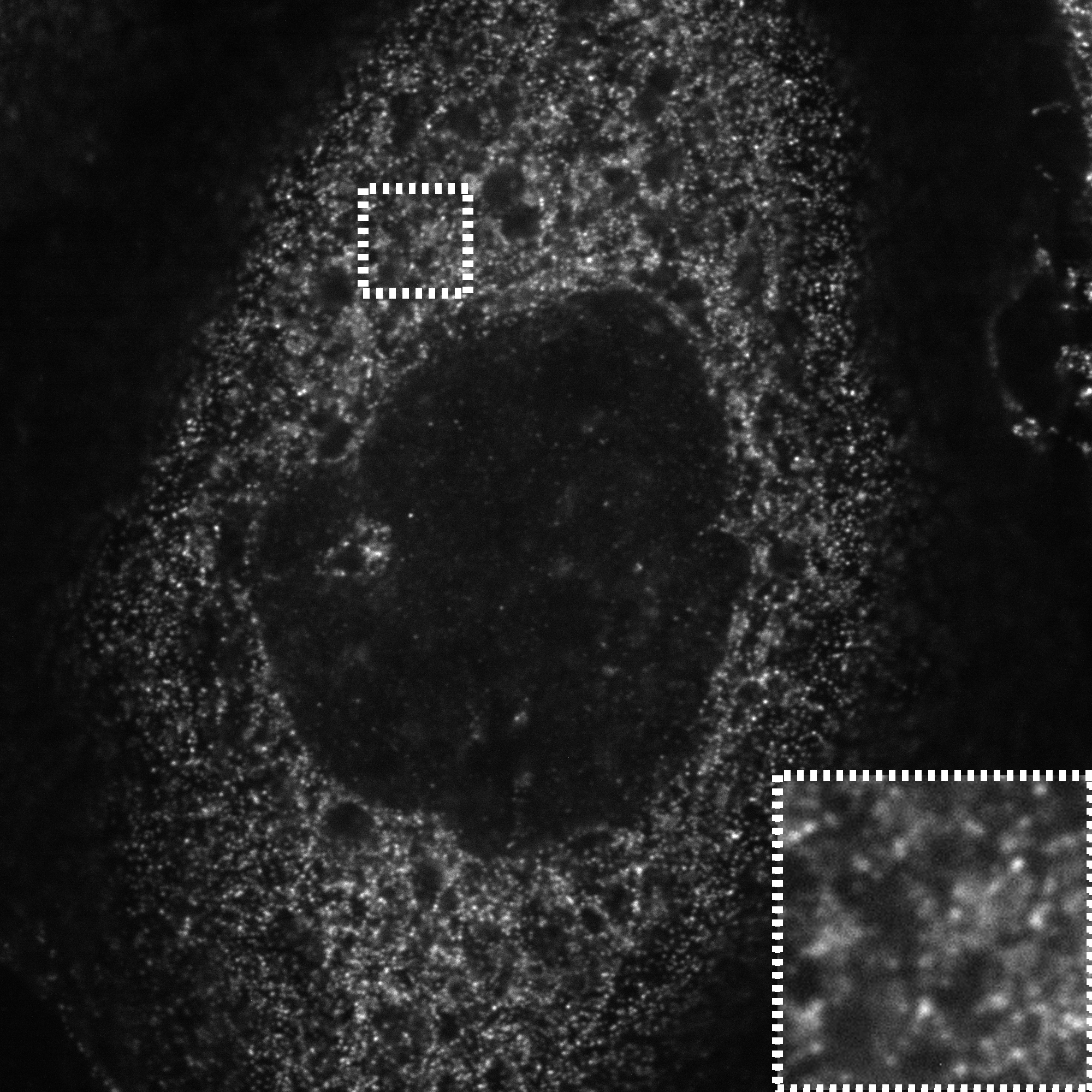

Supplement: Supplementary file 8 — Source data Fig. 6 [file 44318_2024_180_MOESM8_ESM.zip › 6A/TBK1-WT GFP inset.tif]

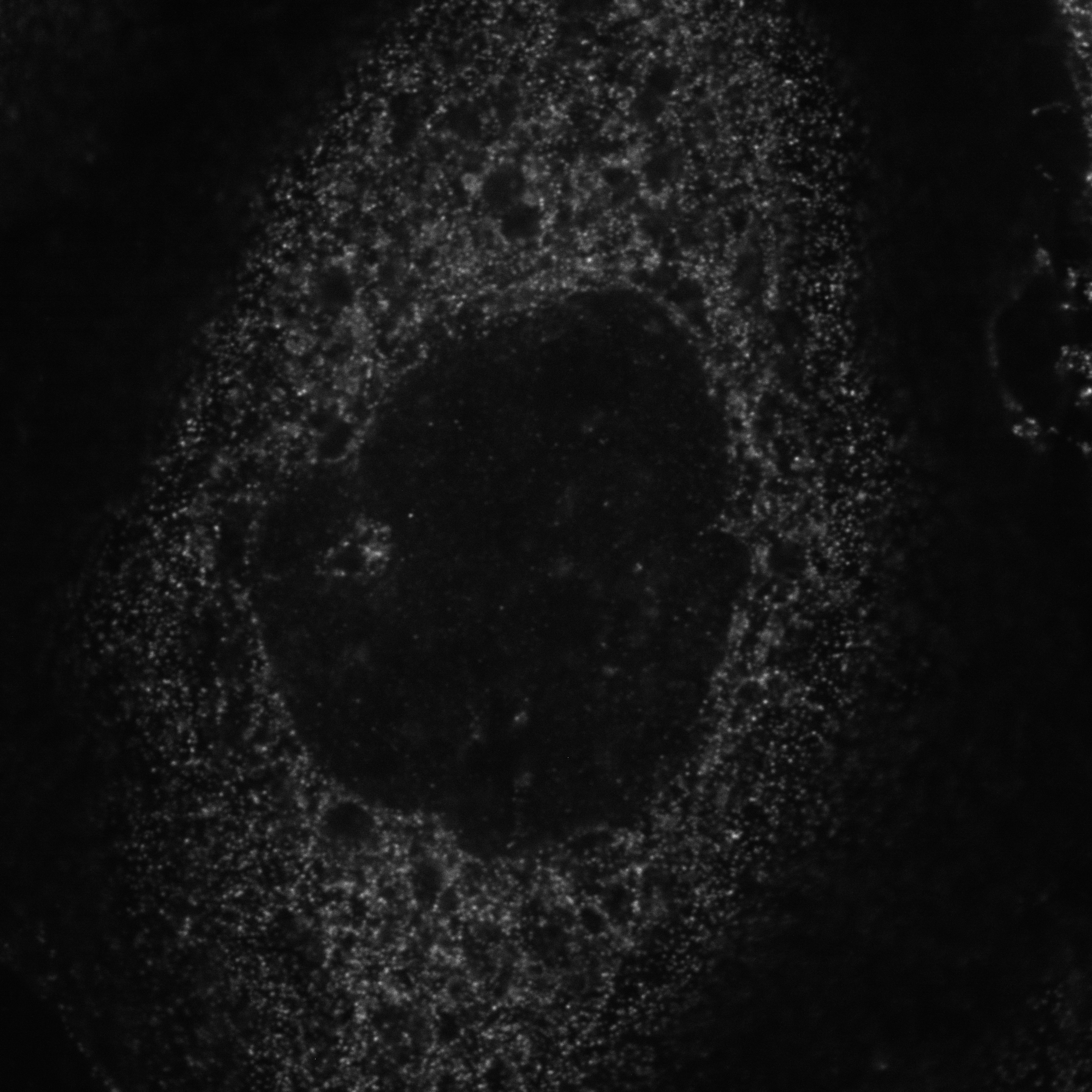

Supplement: Supplementary file 8 — Source data Fig. 6 [file 44318_2024_180_MOESM8_ESM.zip › 6A/TBK1-WT GFP.tif]

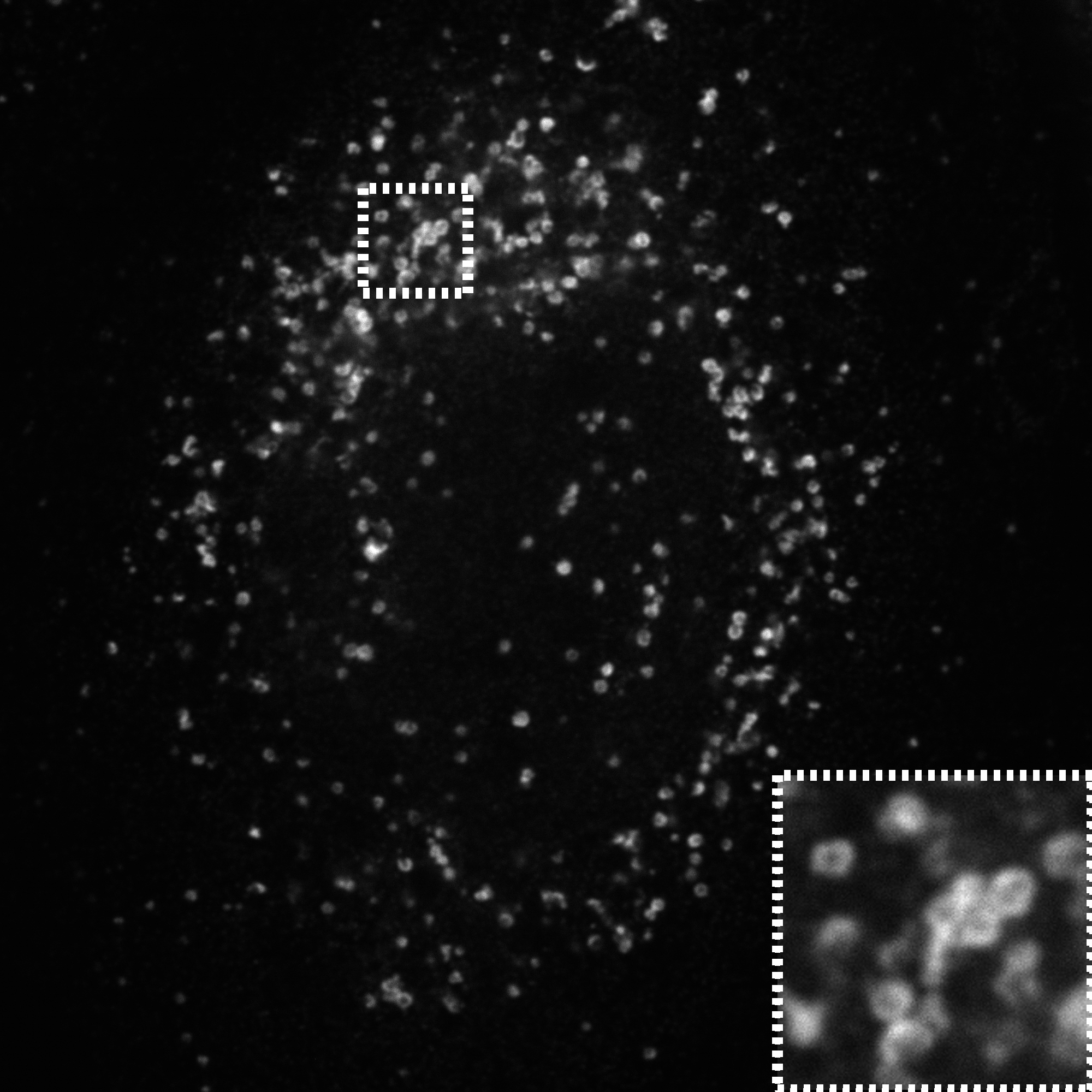

Supplement: Supplementary file 8 — Source data Fig. 6 [file 44318_2024_180_MOESM8_ESM.zip › 6A/TBK1-WT LAMP1 inset.tif]

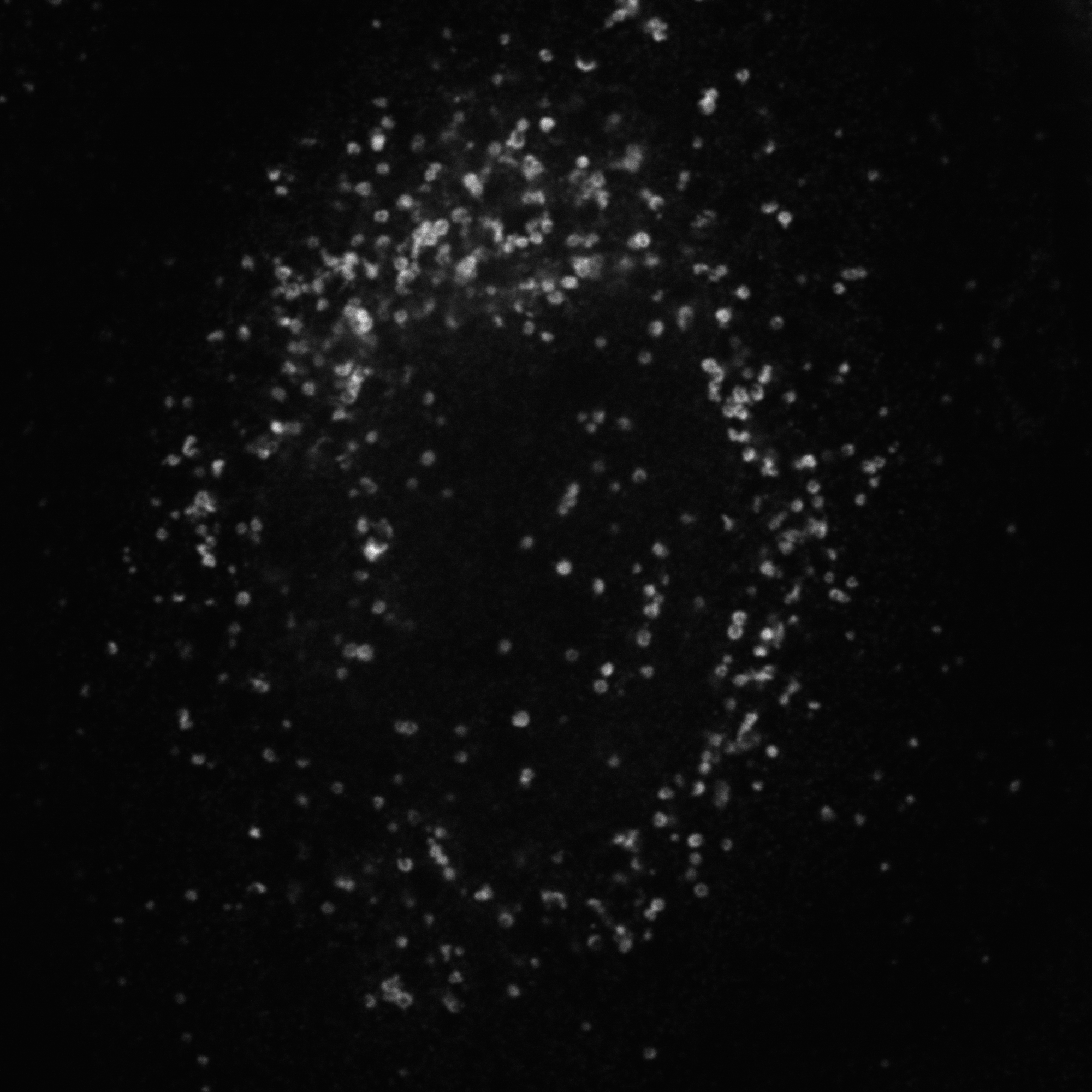

Supplement: Supplementary file 8 — Source data Fig. 6 [file 44318_2024_180_MOESM8_ESM.zip › 6A/TBK1-WT LAMP1.tif]

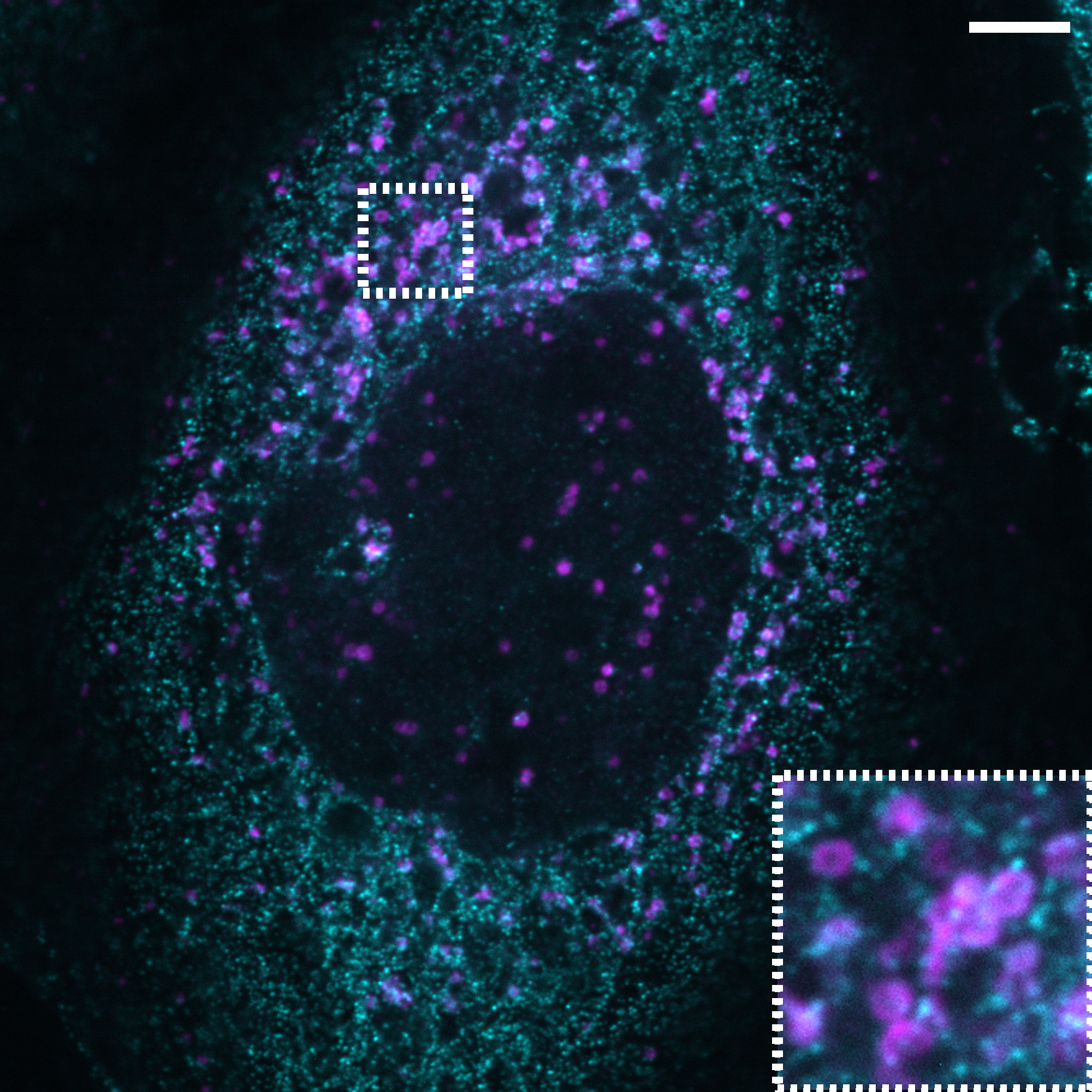

Supplement: Supplementary file 8 — Source data Fig. 6 [file 44318_2024_180_MOESM8_ESM.zip › 6A/TBK1-WT Merge.tif]

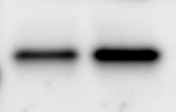

Supplement: Supplementary file 8 — Source data Fig. 6 [file 44318_2024_180_MOESM8_ESM.zip › 6C/pRab7-S72 western cropped.tif]

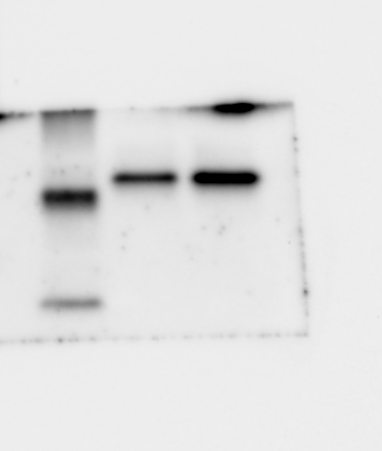

Supplement: Supplementary file 8 — Source data Fig. 6 [file 44318_2024_180_MOESM8_ESM.zip › 6C/pRab7-S72 western.tif]

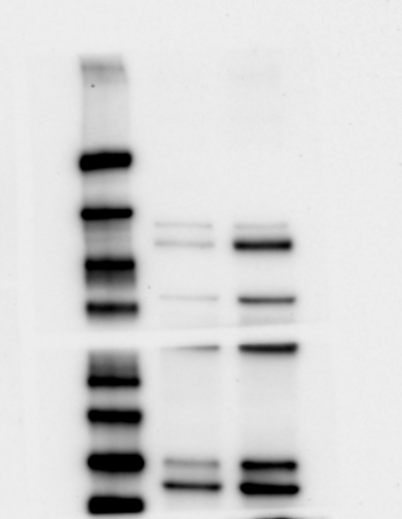

Supplement: Supplementary file 8 — Source data Fig. 6 [file 44318_2024_180_MOESM8_ESM.zip › 6C/pTBK1-S172 and STING western.tif]

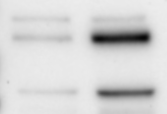

Supplement: Supplementary file 8 — Source data Fig. 6 [file 44318_2024_180_MOESM8_ESM.zip › 6C/pTBK1-S172 western cropped.tif]

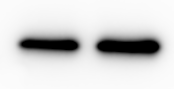

Supplement: Supplementary file 8 — Source data Fig. 6 [file 44318_2024_180_MOESM8_ESM.zip › 6C/Rab7 western cropped.tif]

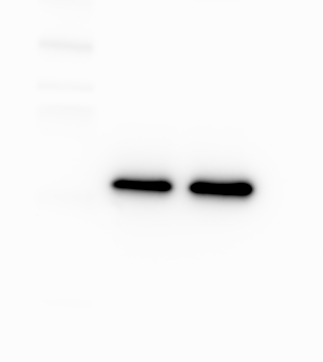

Supplement: Supplementary file 8 — Source data Fig. 6 [file 44318_2024_180_MOESM8_ESM.zip › 6C/Rab7 western.tif]

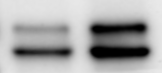

Supplement: Supplementary file 8 — Source data Fig. 6 [file 44318_2024_180_MOESM8_ESM.zip › 6C/STING western cropped.tif]

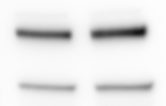

Supplement: Supplementary file 8 — Source data Fig. 6 [file 44318_2024_180_MOESM8_ESM.zip › 6C/TBK1 western cropped.tif]

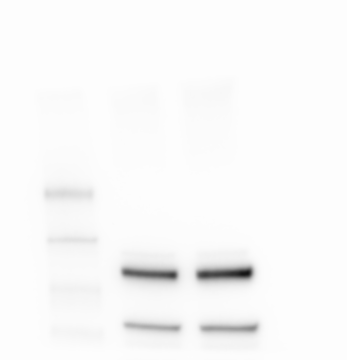

Supplement: Supplementary file 8 — Source data Fig. 6 [file 44318_2024_180_MOESM8_ESM.zip › 6C/TBK1 western.tif]
