# Supplementary figures and images for: Lysosomal TBK1 responds to amino acid availability to relieve Rab7-dependent mTORC1 inhibition (part 3 of 3)
Source: EMBO J. 2024 Aug 5;43(18):7. doi: 10.1038/s44318-024-00180-8 (PMC11405869; doi:10.1038/s44318-024-00180-8)

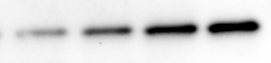

Supplement: Supplementary file 8 — Source data Fig. 6 [file 44318_2024_180_MOESM8_ESM.zip › 6D/pRab7-S72 western cropped.tif]

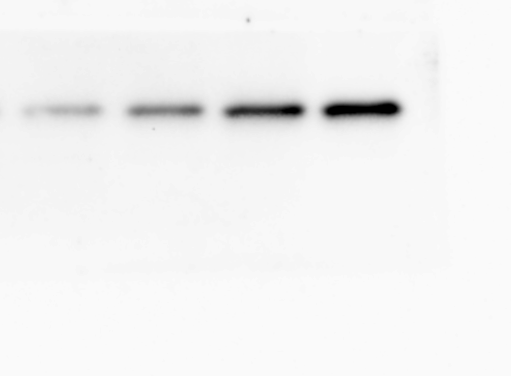

Supplement: Supplementary file 8 — Source data Fig. 6 [file 44318_2024_180_MOESM8_ESM.zip › 6D/pRab7-S72 western.tif]

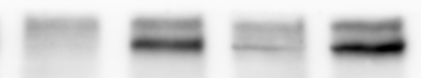

Supplement: Supplementary file 8 — Source data Fig. 6 [file 44318_2024_180_MOESM8_ESM.zip › 6D/pS6K1-T389 western cropped.tif]

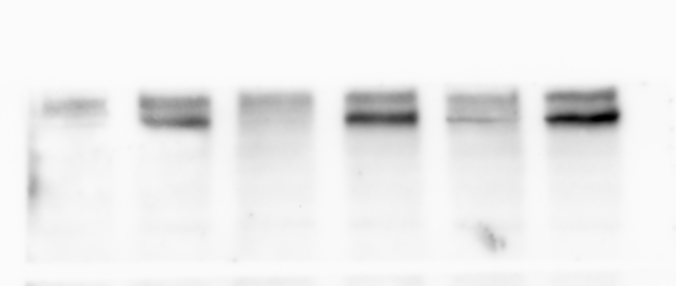

Supplement: Supplementary file 8 — Source data Fig. 6 [file 44318_2024_180_MOESM8_ESM.zip › 6D/pS6K1-T389 western.tif]

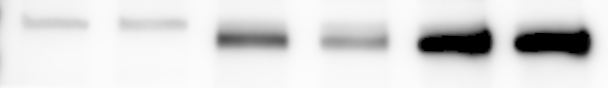

Supplement: Supplementary file 8 — Source data Fig. 6 [file 44318_2024_180_MOESM8_ESM.zip › 6D/pTBK1-S172 western cropped.tif]

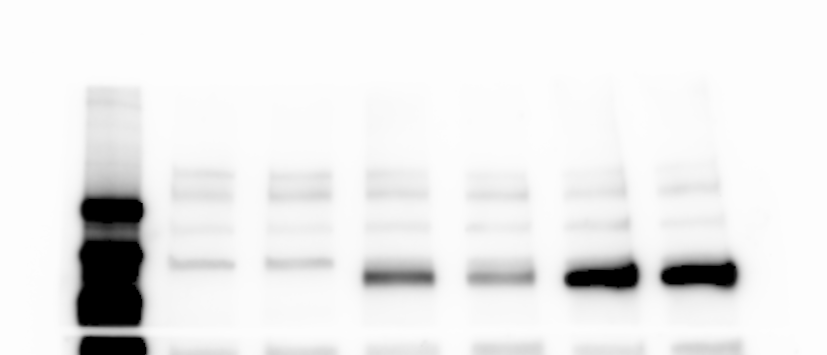

Supplement: Supplementary file 8 — Source data Fig. 6 [file 44318_2024_180_MOESM8_ESM.zip › 6D/pTBK1-S172 western.tif]

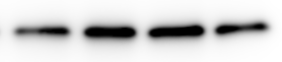

Supplement: Supplementary file 8 — Source data Fig. 6 [file 44318_2024_180_MOESM8_ESM.zip › 6D/Rab7 western cropped.tif]

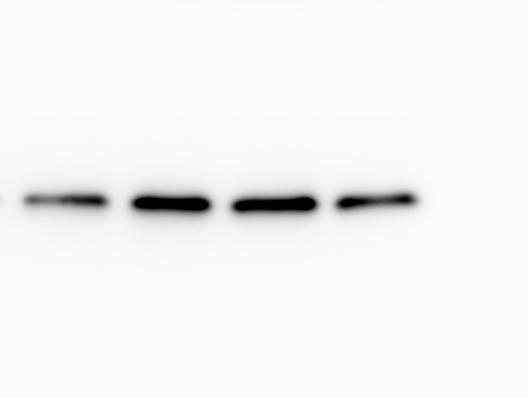

Supplement: Supplementary file 8 — Source data Fig. 6 [file 44318_2024_180_MOESM8_ESM.zip › 6D/Rab7 western.tif]

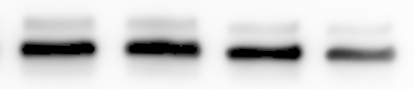

Supplement: Supplementary file 8 — Source data Fig. 6 [file 44318_2024_180_MOESM8_ESM.zip › 6D/S6K1 western cropped.tif]

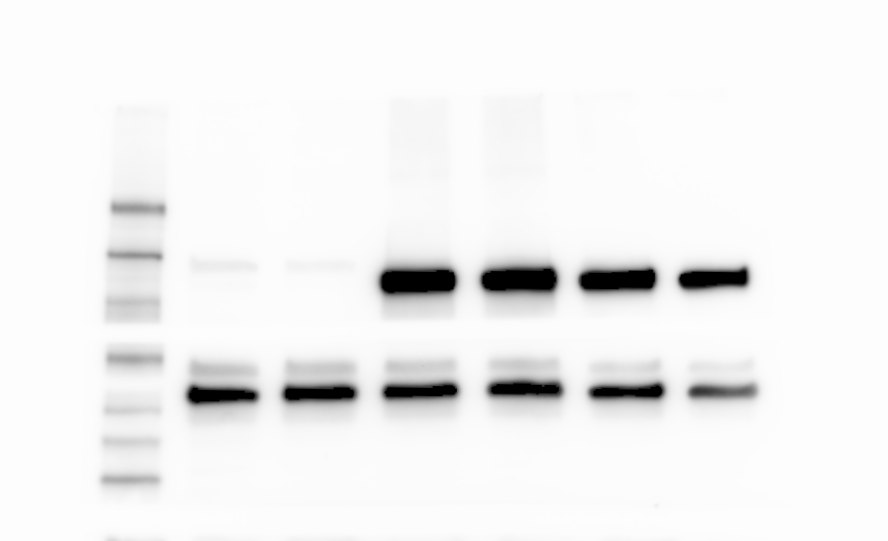

Supplement: Supplementary file 8 — Source data Fig. 6 [file 44318_2024_180_MOESM8_ESM.zip › 6D/TBK1 and S6K1 western.tif]

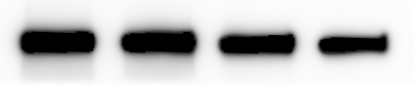

Supplement: Supplementary file 8 — Source data Fig. 6 [file 44318_2024_180_MOESM8_ESM.zip › 6D/TBK1 western cropped.tif]
