# Supplementary material for: Lysosomal TBK1 responds to amino acid availability to relieve Rab7-dependent mTORC1 inhibition
Source: EMBO J. 2024 Aug 5;43(18):7. doi: 10.1038/s44318-024-00180-8 (PMC11405869; doi:10.1038/s44318-024-00180-8)
Supplement: Supplementary file 9 — Expanded View Figures [file 44318_2024_180_MOESM9_ESM.pdf]

Expanded View Figures

Figure EV1. TBK1 is required for efficient amino acid-dependent mTORC1 and cGAMP-dependent STING activation.

(**A**) Immunoblot analysis of phospho-ULK1 at S757, total ULK1, phospho-S6 (S235/S236), and TBK1 of WT and TBK1 KO HeLa cells starved for 60 min (−) and re-fed with amino acids for 60 min (+). (**B**) Quantification of phospho-ULK1 (S757) normalized to total ULK1 and expressed as a fold change compared to the WT cells under re-fed conditions. Data reflets three biological replicates and statistical significance was determined by ordinary one-way analysis of variance (ANOVA) with Šidák *post hoc* test ($n = 3$; mean ± SD; ***$p = 0.0003$). (**C**) Immunoblot analysis of S6K1 phosphorylated at Thr389 [pS6K1 (T389)], total S6K1 and TBK1 in whole-cell lysates from WT versus TBK1 KO RAW 246.7 cells that were starved of amino acids for 60 min (−) and then re-fed with amino acids for 60 min (+). The TGX stain-free method was used to visualize total protein. Asterisk indicates a non-specific band in the phospho-S6K1 blots. (**D**) Quantification of phospho-S6K1 (T389) normalized to total S6K1 and expressed as a fold change compared to the WT cells under re-fed conditions. Data reflets three biological replicates and statistical significance was determined by ordinary one-way ANOVA with Šidák *post hoc* test ($n = 3$; mean ± SD; ***$p = 0.0004$). (**E**) Immunoblot analysis of STING, phospho-TBK1-GFP, and total TBK1-GFP in the lysates (Inputs) and immunoprecipitated TBK1-GFP (IP: GFP) of TBK1 KO HeLa cells stably expressing TBK1-GFP untreated (−) or treated with cGAMP (70 μM) for 120 min (+). (**F**) Immunoblot analysis of pRab7 (S72), total Rab7, and total TBK1-GFP (TBK1) in cell lysates of TBK1 KO + TBK1-GFP HeLa cells under basal fed conditions (+), starved (−) and amino acid re-fed (−/+). (**G**) Phospho-Rab7 levels were quantified and normalized to total Rab7 (basal conditions were considered 1). Statistical significance was determined by ordinary one-way ANOVA with Šidák post-test ($n = 3$; mean ± SD; ****$p < 0.0001$).

 © The Author(s)

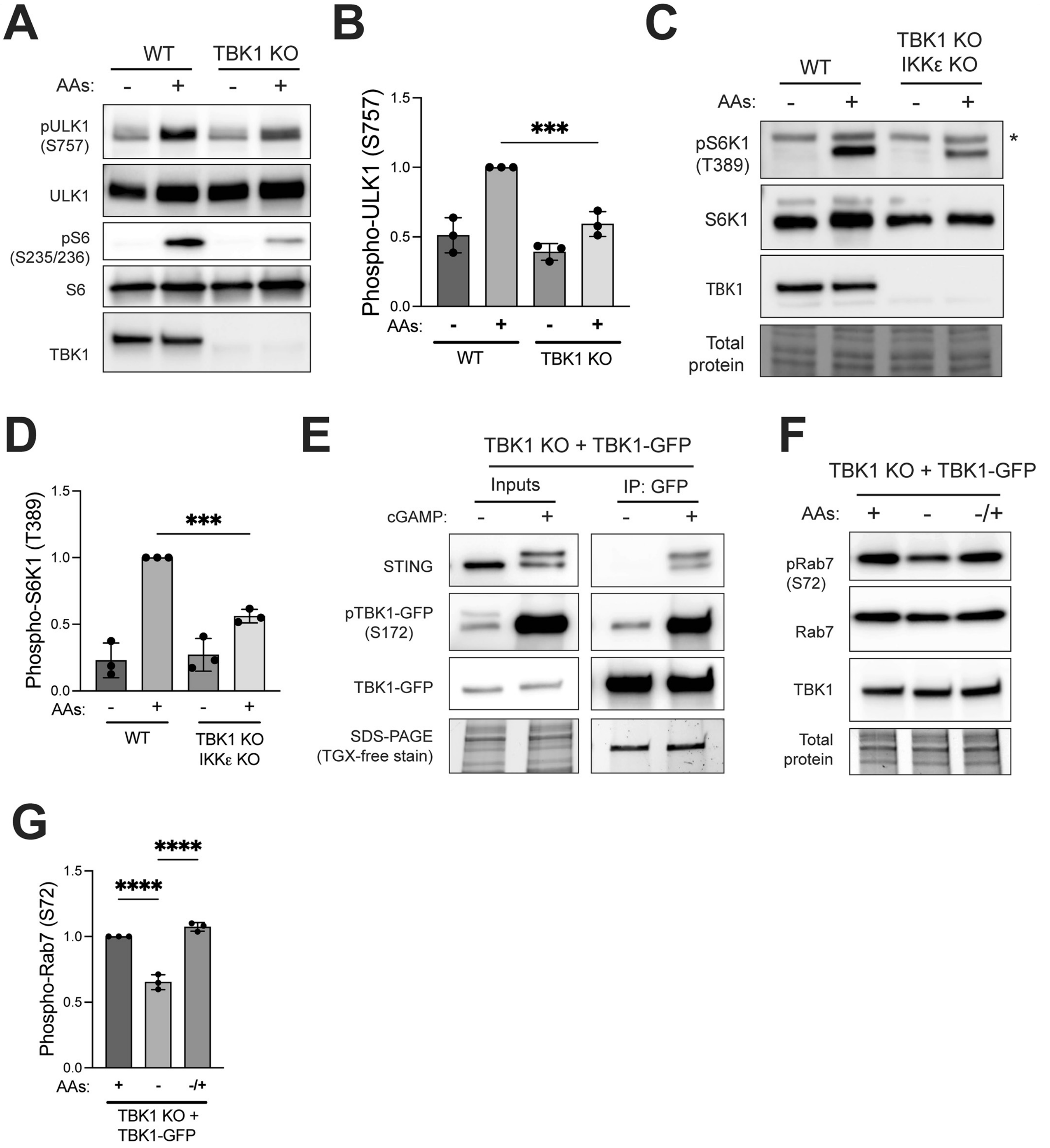
A
WT
TBK1 KO
AAs: - + - +
pULK1 (S757)
ULK1
pS6 (S235/236)
S6
TBK1
B
Phospho-ULK1 (S757)
***
AAs: - + - +
WT
TBK1 KO
C
WT
TBK1 KO IKKε KO
AAs: - + - +
pS6K1 (T389)
*
S6K1
TBK1
Total protein
D
Phospho-S6K1 (T389)
***
AAs: - + - +
WT
TBK1 KO IKKε KO
E
TBK1 KO + TBK1-GFP
Inputs
IP: GFP
cGAMP: - + - +
STING
pTBK1-GFP (S172)
TBK1-GFP
SDS-PAGE (TGX-free stain)
F
TBK1 KO + TBK1-GFP
AAs: + - -/+
pRab7 (S72)
Rab7
TBK1
Total protein
G
Phospho-Rab7 (S72)
****
****
AAs: + - -/+
TBK1 KO + TBK1-GFP

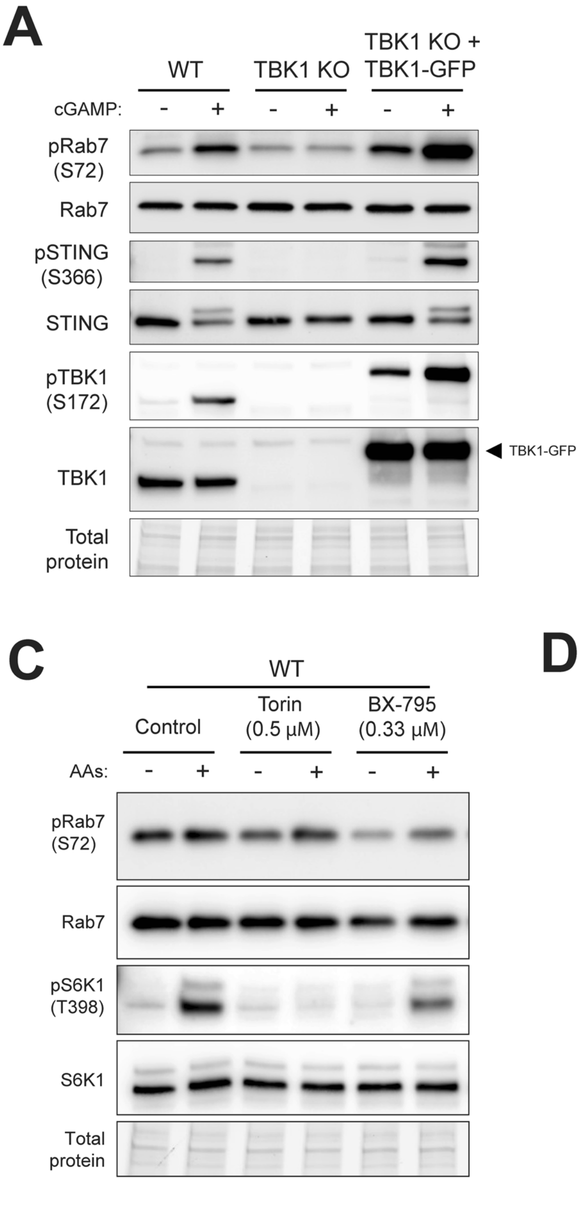

Figure EV2. STING activation triggers TBK1-mediated Rab7 phosphorylation but inactivation of STING or mTORC1 do not prevent amino acid-dependent TBK1 activity.

(**A**) Immunoblot analysis of the indicated proteins in WT, TBK1 KO, and TBK1-GFP HeLa cells untreated (−) or treated with cGAMP (70 µM) for 120 min. (**B**) Immunoblot analysis of the indicated proteins of WT and STING KO RAW 246.7 cells starved for 60 min (−) and then refed with amino acids for 60 min (+). (**C**) Immunoblot analysis of the indicated proteins in WT HeLa cells starved for 60 min (−) and re-fed with amino acids for 60 min (+), untreated (Control), DMSO 0.05% (v/v), treated with Torin (0.5 µM), or treated with BX-795 (0.33 µM). (**D**) Immunoblot analysis of the indicated proteins in WT HeLa cells, transiently transfected with siRNA targeting RAGC (siRagC) or non-targeting/scrambled siRNA (Control), starved for 60 min (−) and re-fed with amino acids for 60 min (+).

 © The Author(s)

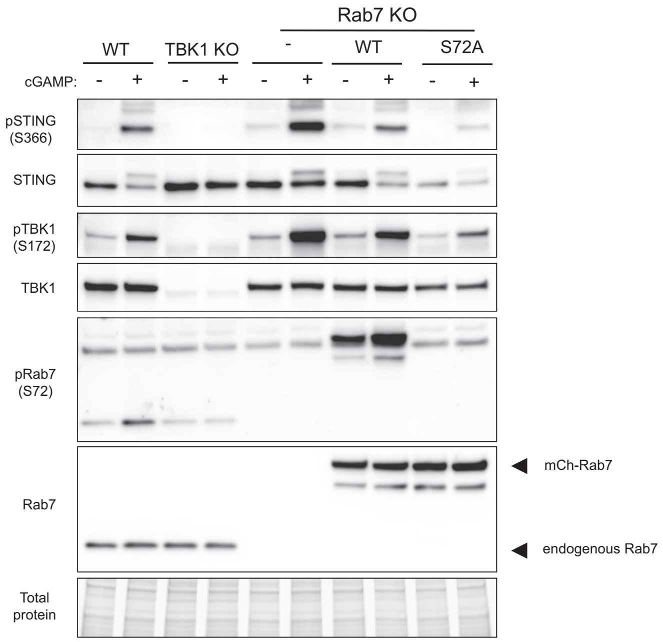

Figure EV3. TBK1-mediated Rab7 phosphorylation regulates STING signaling at lysosomes.

Immunoblot analysis of the indicated proteins of WT, TBK1 KO, Rab7 KO, and Rab7 KO HeLa cells stably expressing mCherry-tagged wild-type or S72A versions of Rab7. These cells were left untreated (−) or were treated with cGAMP (70 μM) for 120 min.

© The Author(s)

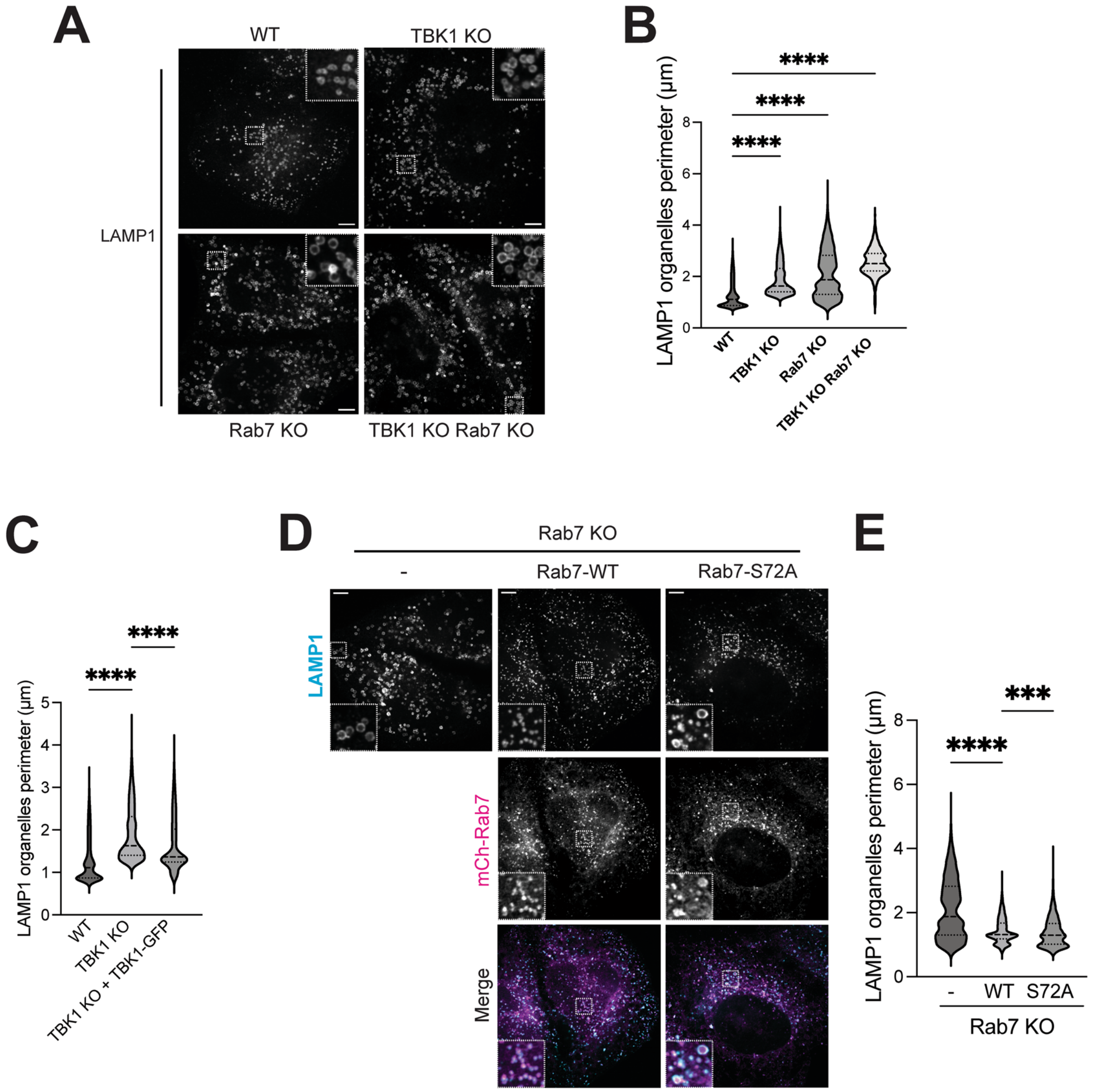

Figure EV4. TBK1 regulates lysosome size independent of Rab7.

(**A**) Immunofluorescence super-resolution spinning disk confocal microscopy analysis of LAMP1 in basal conditions of WT, TBK1 KO, RAB7 KO, TBK1 KO RAB7 KO HeLa cells. (**B**) Quantification of LAMP1-positive organelle (lysosomes) perimeter (µm) in cells of the indicated genotypes. Scale bar: 5 µm. (**C**) Quantification of LAMP1-positive organelle (lysosomes) perimeter from WT, TBK1 KO, and TBK1-GFP rescue data presented in Fig. 1G. (**D**) Immunofluorescence super-resolution microscopy analysis of LAMP1 and mCh-Rab7 in basal conditions for Rab7 KO HeLa cells versus Rab7 KO stably expressing RAB7-WT or RAB7-S72A. (**E**) Quantification of LAMP1-positive organelle (lysosomes) perimeter (µm) in cells of the indicated genotypes. Data plotted in panels (**B**), (**C**) and (**E**) represents results from 2–4 biological replicates; 11–29 regions of interest; 1–2 cells per region of interest. Statistical significance was determined by the Kruskal–Wallis's test followed by Dunn's post-test (Violin Plot; ****$p < 0.0001$; ***$p = 0.0004$). Scale bar: 5 µm.

 © The Author(s)
